# Synthesis and structure elucidation of the human tRNA nucleoside mannosyl-queuosine

**DOI:** 10.1038/s41467-021-27371-9

**Published:** 2021-12-08

**Authors:** Markus Hillmeier, Mirko Wagner, Timm Ensfelder, Eva Korytiakova, Peter Thumbs, Markus Müller, Thomas Carell

**Affiliations:** Department Chemie, Ludwig-Maximilians-Universität München, Butenandtstraße 5-13, 81377 München, Germany

**Keywords:** RNA, Natural product synthesis, Mass spectrometry

## Abstract

Queuosine (Q) is a structurally complex, non‐canonical RNA nucleoside. It is present in many eukaryotic and bacterial species, where it is part of the anticodon loop of certain tRNAs. In higher vertebrates, including humans, two further modified queuosine-derivatives exist ‐ galactosyl‐ (galQ) and mannosyl-queuosine (manQ). The function of these low abundant hypermodified RNA nucleosides remains unknown. While the structure of galQ was elucidated and confirmed by total synthesis, the reported structure of manQ still awaits confirmation. By combining total synthesis and LC-MS-co-injection experiments, together with a metabolic feeding study of labelled hexoses, we show here that the natural compound manQ isolated from mouse liver deviates from the literature-reported structure. Our data show that manQ features an α‐allyl connectivity of its sugar moiety. The yet unidentified glycosylases that attach galactose and mannose to the Q‐base therefore have a maximally different constitutional connectivity preference. Knowing the correct structure of manQ will now pave the way towards further elucidation of its biological function.

## Abstract (web summary)

Mannosyl-queuosine (manQ) is a non-canonical RNA nucleoside present in the anticodon loop of certain tRNAs. Here, the authors use a combination of total synthesis and mass spectrometry to contradict the literature-reported structure and show that manQ features an alpha-allyl connectivity of its mannose moiety.

## Introduction

**Fig. 1:**
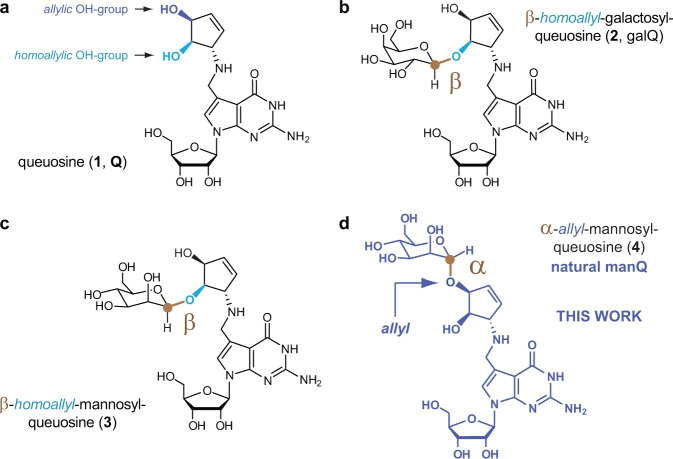
Nucleosides of the queuosine family present in higher vertebrates. **a** Queuosine 1 is derived from a 7-deazaguanosine base with a 1(S)-amino-2(R),3(S)-dihydroxycyclopent-4-ene unit attached via a methylene linker to the C7-position of the 7-deazaguanosine. Possible glycosylation sites are depicted in dark blue (allylic hydroxyl group) and light blue (homoallylic hydroxyl group); **b** Structure of the naturally occurring nucleoside β-homoallyl-galQ 2; **c** Structure 3 shows the originally proposed structure of manQ, which we revise in this work; **d** Corrected structure of natural manQ 4 featuring an α-allylic connectivity.

Ribonucleic acid (RNA) is a central molecule of life, linking the genotype to the phenotype by integrating both catalytic and coding properties in the synthesis of proteins^1,2^. To fulfil the plethora of functions known for RNA today, a huge chemical diversity has developed regarding the nucleobases through the evolution of life^3,4^. The most densely modified RNA molecules are the tRNAs^5^. Those small adaptor molecules feed the required amino acids to the growing peptide chain in the ribosome. Of particular interest are the non-canonical nucleosides that are found in the anticodon loop of tRNAs, in particular at position 34, known as the Wobble position of the anticodon^6^. Here, any chemical modification has a direct impact on the coding potential of the tRNA^6–9^. Queuosine (1, Q, Fig. 1a) is a particularly complex non-canonical nucleoside^10–12^. It is found in many bacterial and eukaryotic species, where it is located at position 34 of the anticodon loop of tRNA^Tyr^, tRNA^Asp^, tRNA^His^ and tRNA^Asn^^13,14^.

In eukaryotes, queuosine 1 is found in both cytosolic and mitochondrial tRNA^15^. It is biochemically derived from guanosine (G) and characterised by the exchange of the N7‐nitrogen for a C7‐carbon atom and the addition of a 1(S)‐amino‐2(R),3(S)‐dihydroxycyclopent‐4‐ene unit to the C7‐position via a methylene linker^12,16^. The presence of the Q-base in position 34 of the anticodon equips the corresponding tRNAs with the possibility to decode synonymous codons by wobble base pairing^17,18^. The Q‐base is furthermore affecting translational speed^19^, decoding fidelity^20^, and it is essential for survival particularly in the absence of sufficient tyrosine^21,22^. Eukaryotes are unable to biosynthesize Q, which forces them to acquire it from prokaryotic sources^21,23–25^. This establishes a link between the central process of translation and the gut microbiome^26^. Defective RNA modification processes are increasingly recognised as drivers for severe diseases^27,28^.

In higher vertebrates, including humans, two glycosylated queuosine derivatives exist, which have yet unidentified functions. The first of these hypermodified Q derivatives is found in cytosolic tRNA^Tyr^, where the Q-base is modified with a galactose residue (galQ, 2, Fig. 1b)^10,29^. The cytosolic tRNA^Asp^, in contrast, contains a Q-derivative, which is suggested to be mannosylated (manQ, Fig. 1c, d). A first effort to elucidate the structure of these nucleosides was reported in 1976 by Kasai et al. They derived the structure of galQ and proposed a structure for manQ based on NMR- and MS-data that was obtained from isolated material from rabbit liver. This data lead to the proposal of structure 3 for natural manQ^29^. Here, we report the total synthesis of the human natural product manQ and show that the originally proposed structure 3 (Fig. 1c) needs to be revised in two important aspects: in contrast to the original structure suggestion, we show that the mannose glycosidic bond is not β- but α-configured. Furthermore, and in contrast to β-galQ, the mannose is attached not to the homoallylic, but to the allylic hydroxyl group at C3. manQ has consequently the structure 4 shown in Fig. 1d. This result reveals that the yet unknown galactosyl and mannosyl transferases that attach the respective hexose to the Q-nucleobase in tRNA^Tyr^ and tRNA^Asp^, are able to differentiate the two hydroxyl groups (allyl vs. homoallyl) at the cyclopentene substructure for yet unknown reasons.

## Results and discussion

**Fig. 2:**
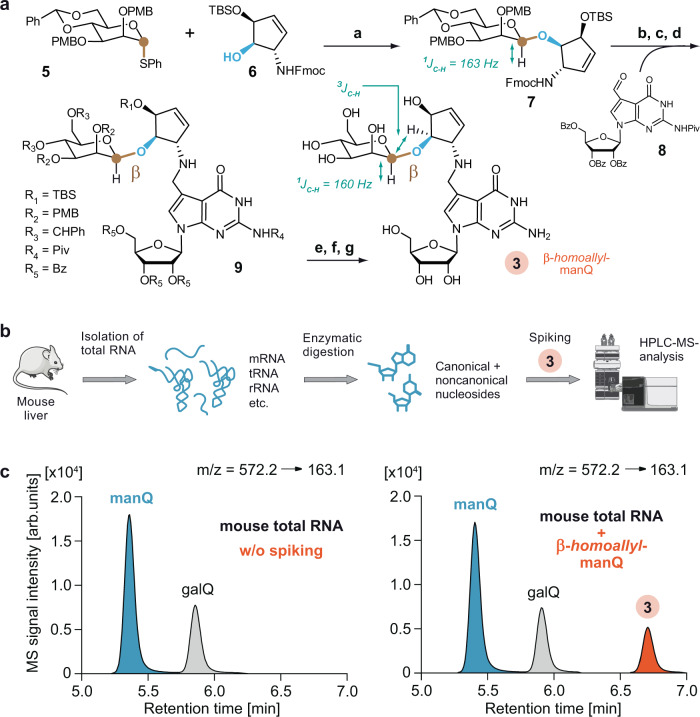
Attempt to confirm the originally proposed structure of manQ. **a** Synthetic route towards β-homoallyl-manQ featuring a β-selective mannosylation key step; a: AgOTf, PhSCl, DTBMP, DCM, −78 °C, 2 h, 62%; b: HNMe_2_, THF, rt, 1 h; c: 8, MeOH, rt, 5 h; d: NaBH_4_, MeOH, 0 °C, 1 h; e: NaOMe, MeOH, rt, 5 h; f: TFA, DCM, 0 °C, 20 min; g: HF ⋅ pyridine, pyridine, rt, 18 h, 22% (5 steps); **b** Workflow for the comparison of the synthetic β-homoallyl-manQ standard with natural manQ via isolation of total RNA from mouse liver, digestion to the nucleoside level and LC-MS-co-injection experiments; **c** Chromatograms resulting from the co-injection experiments analysed by UHPLC-MS/MS; left: digested total RNA from mouse liver shows two peaks, natural manQ and galQ_._; right: digested total RNA from mouse liver spiked with the synthetic standard β-homoallyl-manQ 3 shows the appearance of an additional peak, thereby disproving the originally proposed structure for manQ. m/z = 572.2 is both the mass of the protonated manQ or galQ nucleoside, while m/z = 163.1 is the mass of a specific fragment ion formed during the fragmentation of these molecules in the mass spectrometer (see Supplementary Fig. 1). This mass transition was used by us for manQ and galQ detection via MS/MS.

Synthesis of literature-reported manQ. The compounds galQ and manQ are found in the anticodon loop of one cytosolic tRNA each^30^. Consequently, the amounts that can be isolated from nature (e.g. porcine liver) are very small, so that a structure elucidation from isolated material only is very difficult. tRNA^Asp^, which contains one molecule of manQ per tRNA, can be isolated^30^, but in our hands it was impossible to obtain sufficient amounts for a full structure elucidation. Therefore, synthesis of the proposed manQ compound and comparison of the synthetic standard with natural material by LC-MS was the method of choice for us to investigate the manQ structure. The synthesis of compound 3, proposed to be natural manQ, was performed as depicted in Fig. 2a.

For the synthesis of the difficult-to-access β-mannosyl connectivity, we employed the Crich-method^31^, which required the preparation of the mannosyl donor 5. This was achieved along the literature-reported synthesis^31,32^. The mannosylation reaction of the Fmoc- and TBS- protected 1(S)-amino-2(R),3(S)-dihydroxycyclopent-4-ene unit 6 with the mannosyl donor 5 required an activation with Ag-triflate in the presence of phenylthiochloride and 2,6-di-tert-butyl-4-methylpyridine (DTBMP) at −78 °C in dry dichloromethane. The reaction gave the mannoside 7 in 62% yield, and the β-configuration of its anomeric centre was confirmed by NMR-spectroscopy based on the coupling constant of ^1^J_C1-H1_ = 163 Hz, which is typical for β-mannosides^33^. We next cleaved the Fmoc group of the mannoside 7 with dimethylamine (10% DMA in THF, rt, 1 h) and performed the reductive amination with the benzoyl- and pivaloyl-protected 7-formyl-7-deazaguanosine compound 8 (MeOH, rt, 5 h, then NaBH_4_, 0 °C, 1 h) that was prepared as recently described by us^10^. This afforded the fully protected manQ compound 9. Cleavage of the benzoyl-protecting groups at the ribose was performed under Zemplén-conditions (0.5 m NaOMe, MeOH, rt, 5 h). The PMB-ethers were deprotected with trifluoro acetic acid in dichloromethane (10%, 0 °C, 20 min). Finally, we removed the TBS-protecting groups with HF in pyridine (rt, 18 h). This furnished the final manQ compound 3 with mannose being β-configured and attached to the homoallylic hydroxyl group. As expected, compound 3 has a ^1^J_C-H_-coupling constant of 160 Hz at the anomeric centre, thereby proving its β-configuration. Furthermore, the observed ^3^J_C-H_-HMBC coupling in compound 3 between the mannosyl C1-carbon and the homoallylic hydrogen C4H of the cyclopentene ring (see Supplementary Information 4) unambiguously proved that the mannosyl residue is indeed connected to the homoallylic hydroxyl group.

In order to compare the synthetic manQ compound 3 with the natural material, we performed an LC-MS co-injection study. To this end, we isolated the total RNA from mouse liver and enzymatically digested this RNA down to the individual nucleosides as recently described by us^10^. This procedure afforded a mixture of all nucleosides present in the total RNA pool. Subsequent analysis of the resulting nucleoside mixture was performed via UHPLC-MS/MS with a triple quadrupole mass spectrometer (QQQ) set to monitor a specific molecular fragmentation reaction of the glycosylated Q-derivatives. Using collision-induced dissociation, these nucleosides undergo two heterolytic bond dissociations, leading to a loss of both the ribose sugar and the glycosylated cyclopentene unit. Monitoring the corresponding mass transition of m/z = 572.2 → 163.1 allows a sensitive detection of these compounds (see Fig. 2b and Supplementary Fig. 1). With an UHPLC separation gradient of 0 → 2% (v/v) MeCN/H_2_O in 0 → 8 min on a Poroshell 120 SB-C8 column, all hexose-modified queuosine derivatives can be separated chromatographically (see Fig. 2c). Indeed, when analysing the mouse liver sample, we detected two clearly separated signals in the RNA nucleoside pool with galQ 2 eluting at 5.9 min, and the natural manQ compound eluting at 5.4 min, respectively. The compound eluting at 5.9 min was unambiguously identified as galQ 2 with the help of a synthetic reference compound (see Supplementary Fig. 2). Upon co-injection of the synthetic material 3, we were expecting to see again two signals with the manQ signal having gained in intensity. To our surprise, however, we noted that this is not the case. Instead, the co-injection experiment provided a third signal with a retention time of 6.8 min, clearly separated from both natural manQ and galQ 2. This result unequivocally shows that manQ 3 is NOT identical to the natural manQ compound. Therefore, the structure proposal for manQ reported in literature must be incorrect.

**Fig. 3:**
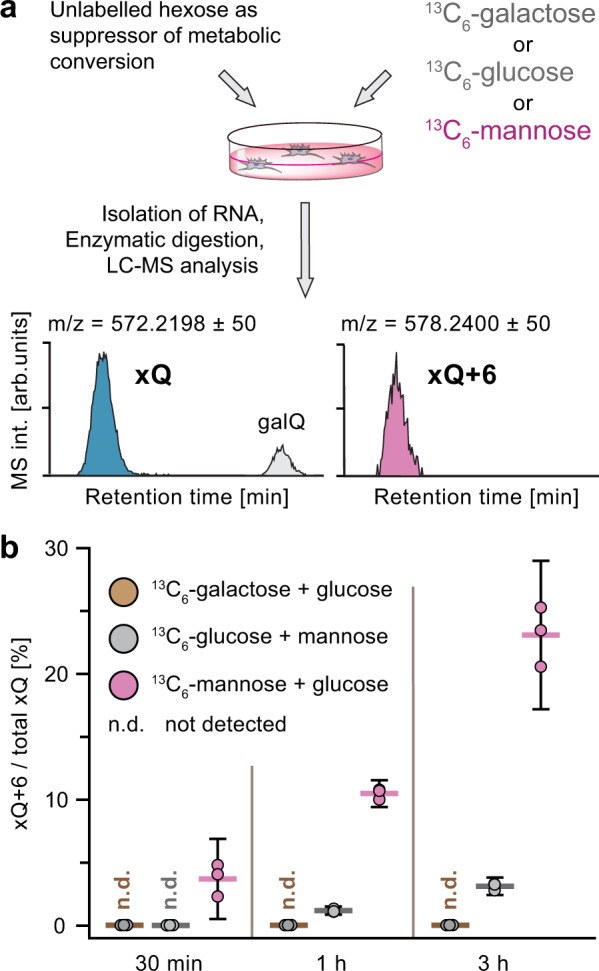
Confirmation of the incorporated hexose sugar as being mannose. **a** Workflow of the metabolic labelling study providing ^13^C_6_-galactose, ^13^C_6_-glucose, or ^13^C_6_-mannose in combination with an unlabelled carbon source (glucose or mannose) that suppresses metabolic interconversion of the carbohydrates and hence isotope scrambling; **b** Results of the LC-MS experiments showing quick incorporation of heavy sugar when feeding ^13^C_6_-mannose in presence of glucose, thereby confirming the identity of xQ as manQ; three independent experimental replicates were performed for each condition tested (coloured dots). Mean values are indicated by coloured horizontal lines, while whiskers enclose the 95%-confidence interval.

Puzzled by this result, we initially reasoned that the compound originally identified as manQ may contain a different sugar than mannose, even though a previous study showed that a mannosyl moiety can enzymatically be transferred to the Q-base from GDP-mannose^34^. To verify that manQ contains indeed a mannose sugar, we performed a metabolic labelling study (Fig. 3a): We added different isotope-labelled sugars to a HEK293T cell culture and subsequently isolated the total RNA of the cells to see if the natural manQ had incorporated the isotope labels. During these experiments, the cell culture medium was additionally supplemented with high concentrations of unlabelled glucose or mannose as carbon source in order to suppress the metabolic conversion of the labelled sugar into other carbohydrates^35^. Without these metabolic suppressors, we observed in our experiments an unwanted isotope scrambling process that jeopardised the experiments. The results of our study are depicted in Fig. 3b. It is clearly evident that feeding of ^13^C_6_-galactose and ^13^C_6_-glucose in the presence of the metabolic suppressors glucose or mannose, respectively, gave little or no incorporation of ^13^C into the isolated natural manQ compound. In contrast, feeding of ^13^C_6_-mannose in combination with the metabolic suppressor glucose quickly led to the time-dependent formation of ^13^C_6_-manQ, thereby confirming that the sugar connected to the Q-base in manQ is indeed mannose.

**Fig. 4:**
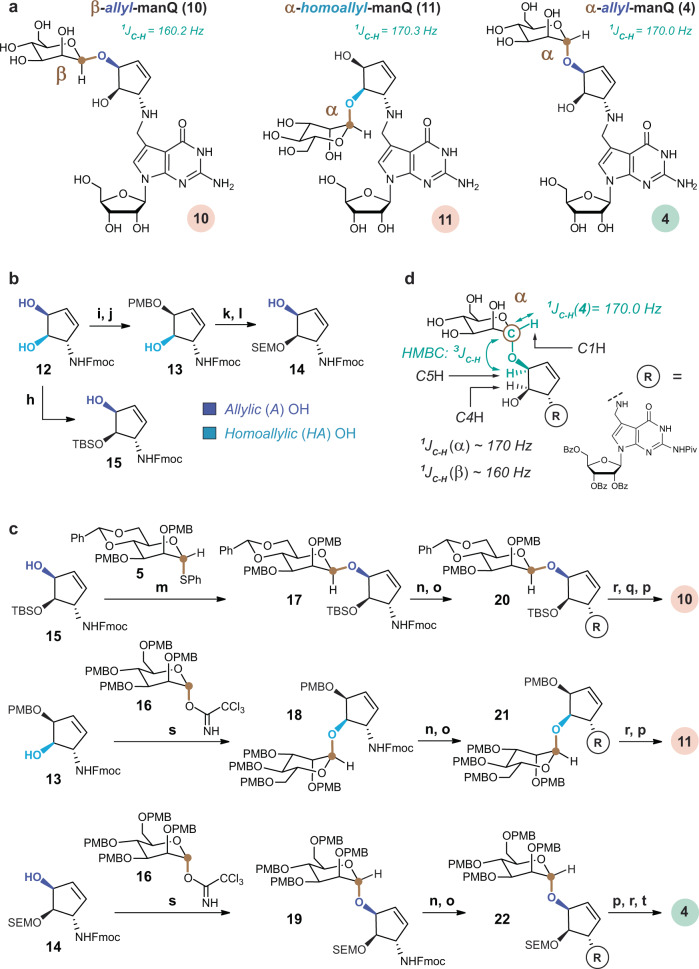
Alternative manQ structures and their syntheses. **a** manQ target compounds: β-allyl-manQ 10, α-homoallyl-manQ 11 and the correct structure 4 of natural manQ featuring an α-allyl-connectivity; **b** Regioselective protection of cyclopentene 12 for the synthesis of precursors 13–15; h: TBSOTf, DMF, −10 °C, 2 h, 41%; i: anisaldehyde dimethyl acetal, CSA, DMF, rt, 2 h, 98%; j: DIBAL-H, DCM, −78 °C, 3 h, 85%; k: SEM-Cl, NBu_4_I, pyridine, DMF, 70 °C, 18 h, 75%; l: TFA, DCM, 0 °C, 5 min, 83%; **c** Synthesis of the manQ compounds 10, 11 and 4 from 13, 14 and 15 via an appropriate stereoselective glycosylation step and subsequent reductive amination; m: AgOTf, PhSCl, DTBMP, DCM, −78 °C, 2 h, 52%; n: DBU, MeCN, rt, 1 h (10) or HNMe_2_, THF, rt, 1 h (4, 11); o: 8, MeOH, 2–5 h, then NaBH_4_, 0 °C, 15–30 min; p: NaOMe, MeOH, rt; q: HF ⋅ pyridine, EtOAc, rt, 18 h; r: TFA, DCM, 0 °C; s: TMSOTf, THF, 0 °C; t: HF ⋅ pyridine, MeCN, rt, 1 week; **d** Depiction of the ^1^J_C-H_-coupling at the anomeric centre of α-allyl-manQ 4, and of the ^3^J_C-H_-HMBC-coupling between its anomeric mannose carbon C1 and the C5-hydrogen atom of its cyclopentene moiety. Both couplings were used to confirm the structure of this compound via NMR, and similar couplings were used to confirm the structures of 3, 10 and 11.

We therefore reasoned that natural manQ differs from the reported structure 3 likely regarding the configuration of the anomeric centre and/or the connectivity at the cyclopentene moiety (allyl versus homoallyl mannoside). With 3 proven not to be the natural compound, we were left with the three remaining structures 4 (α-allyl-connectivity), 10 (β-allyl-connectivity) and 11 (α-homoallyl-connectivity, see Fig. 4a). For their synthesis, it was necessary to first develop syntheses for the properly protected 1-amino-2,3-dihydroxycyclopent-4-enes 13–15 (Fig. 4b). To access these compounds, we started with the Fmoc-protected 1-amino-2,3-dihydroxypent-4-ene 12. For the synthesis of the 1-Fmoc-3-PMB-protected cyclopent-4-ene 13, needed for the synthesis of 11, we first protected in 12 both OH groups as an anisaldehyde acetal (anisaldehyde dimethyl acetal, CSA, rt, 2 h, 98%), followed by a selective reductive opening of the homoallylic OH group with DIBAL-H. This gave the 1-Fmoc-3-PMB-protected-dihydroxycyclopent-4-ene 13 (DCM, −78 °C, 3 h, 85%). For 14, we used 13 as the starting material. Protection of the homoallylic hydroxyl group in 13 with SEM-Cl (NBu_4_I, pyridine, DMF, 70 °C, 18 h, 75%), followed by cleavage of the p-methoxybenzylether (10% TFA in DCM, 0 °C, 5 min, 83%) furnished compound 14. Reaction of 12 with TBSOTf at −55 °C provided after 15 min predominantly the 1-Fmoc-3-TBS-protected compound 6 as the kinetic product, which we had used before for the synthesis of the literature-reported structure of manQ 3. If this reaction was, however, performed at −10 °C for a longer period of time (2 h), we observed formation of the thermodynamically more stable 1-Fmoc-2-TBS-protected compound 15.

With the differently protected cyclopentenes 13, 14 and 15 in hand, the manQ compounds 10, 11 and 4 were synthesised via the glycosylation and reductive amination approach (Fig. 4c). The first target compound, β-allyl-manQ 10, was prepared from cyclopentene precursor 15 starting with a stereoselective β-mannosylation reaction (AgOTf, PhSCl, 2,6-di-tert-butyl-4-methylpyridine, DCM, −78 °C, 2 h, 52%) to give the glycosylated product 17. Fmoc-deprotection (DBU, MeCN, rt, 1 h) and subsequent reductive amination with deazaguanosine precursor 8 gave the fully protected β-allyl-manQ 20. A three-step final deprotection procedure (1: 10% TFA in DCM; 2: HF pyridine, EtOAc, rt, 18 h; 3: NaOMe, MeOH, 2 d) gave β-allyl-manQ 10. For the second target compound, α-homoallyl-manQ 11, we started from cyclopentene precursor 13 and employed Schmidt-Sinaӳ-glycosylation conditions with the literature-known glycosyl donor 16 (TMSOTf, THF, 0 °C, 55%) to obtain glycosylated product 18^36,37^. The synthesis of α-homoallyl-manQ 11 was completed from there by Fmoc-deprotection, reductive amination with 8, and a two-step final deprotection (1: 10% TFA, DCM, 30 min; 2: NaOMe, MeOH, rt, 2 d). The third target compound, α-allyl-manQ 4, was synthesised again via Schmidt-Sinaӳ-glycosylation conditions with donor 16^36,37^. In this case, the SEM-protected cyclopentene precursor 14 was used, because the TBS-protected precursor 15 proved to be sterically too demanding for the glycosylation reaction. Reaction of 14 with 16 however was not fully stereoselective. But after full completion of the synthesis by Fmoc-deprotection, reductive amination and subsequent three-step deprotection (1: NaOMe, MeOH, rt, 18 h; 2: 10% TFA, DCM, 0 °C, 15 min; 3: HF ∙ pyridine, MeCN, rt, 1 week), we obtained a mixture of 10 and 4, which was separated by reversed-phase HPLC-chromatography (see Supplementary Information 3.5). This allowed us to obtain α-allyl-manQ 4 in excellent purity.

We next confirmed the structure of the manQ compounds 4, 10 and 11 using NMR spectroscopy (Fig. 4d). For β-allyl-manQ 10, we observed a ^1^J_C-H_-coupling at the mannose anomeric centre of 160.2 Hz, indicative of the β-configuration. The allylic connectivity of 10 was confirmed by the ^3^J_C-H_-HMBC-coupling between the anomeric mannose C1 carbon and the allylic hydrogen C5H of the cyclopentene ring. For α-homoallyl-manQ 11, a ^1^J_C-H_-coupling of 170.3 Hz was observed at the anomeric centre, proving its α-configuration. In addition, with *δ* = 5.04 ppm, the chemical shift of the anomeric proton C1H of 11 was as expected higher than the shift observed for the corresponding β-anomer 3 (*δ* = 4.72 ppm). Homoallylic connectivity of 11 was confirmed by observation of an ^3^J_C-H_-HMBC-coupling of the anomeric carbon C1 and the homoallylic hydrogen C4H of the cyclopentene moiety. The structure of α-allyl-manQ 4 was proven based on an anomeric ^1^J_C-H_ -coupling of 170.0 Hz, indicating its α-configuration. Here, the chemical shift of the anomeric proton C1H (*δ* = 4.97 ppm) was again higher than that of the corresponding β-anomer 10 (*δ* = 4.70 ppm), thereby also confirming the α-configuration of 4. The allylic connectivity of 4 was confirmed by an ^3^J_C-H_-HMBC signal resulting from a coupling of the anomeric mannose carbon C1 with the allylic hydrogen C5H of the cyclopentene ring.

**Fig. 5:**
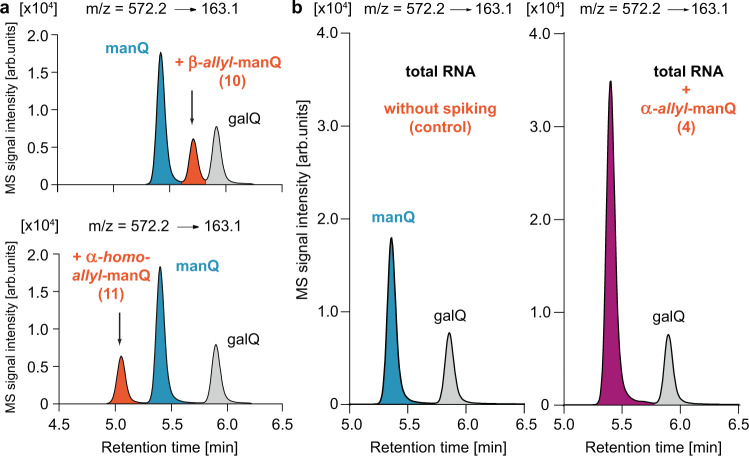
Chromatograms resulting from the co-injection of the synthetic manQ compounds 10, 11 and 4 with enzymatically digested total RNA from mouse liver analysed by UHPLC-MS/MS. **a** Co-injection of β-allyl-manQ 10 and α-homoallyl-manQ 11 show the appearance of a third peak, these compounds are therefore different from natural manQ; **b** Co-injection of α-allyl-manQ 4 with digested total RNA from mouse liver shows a complete signal overlap of 4 and natural manQ, thereby leading to an increased signal intensity of manQ (right) in comparison to the control sample without synthetic standard (left). This result shows that our synthetic compound 4 is identical to the naturally occurring manQ nucleoside and establishes its α-allyl-connectivity.

With all four possible manQ isomers now available (α/β-allyl-manQ 4 and 10, and α/β-homoallyl-manQ 3 and 11), we next performed individual co-injection studies using again a digest of total RNA from mouse liver as reference material, and the UHPLC-MS/MS-method described above for analysis (Fig. 5). While for β-allyl-manQ 10 and α-homoallyl-manQ 11 LC-MS signals were obtained that were well-separated both from galQ 2 and natural manQ (Fig. 5a), we discovered to our delight a full signal overlap of our synthetic α-allyl-manQ 4 with the natural manQ compound (Fig. 5b) We finally confirmed the overlap of the two compound signals with a second HPLC-MS-method using a different HPLC separation column and gradient (see Supplementary Fig. 3). Here, too, the synthetic material co-eluted with the natural manQ compound. These co-injection experiments therefore show that the natural manQ compound present in the anticodon loop of tRNA^Asp^ has the chemical structure 4 (see Figs. 1d and 4a). The configuration of its anomeric centre is in fact α, not β. In addition, the mannosyl moiety of natural manQ is connected to the allylic hydroxyl group of the cyclopentene unit, and not to the homoallylic hydroxyl group as in galQ.

To prove that our RNA isolation and digestion conditions do not cause isomerisation of the manQ compounds 3, 4, 10 and 11, we performed stability tests under these conditions with all four isomers (see Supplementary Figs. 4 and 5). In these experiments we observed no isomerisation processes. Finally, we exposed compounds 3 and 4 to neutral (pH 7), basic (0.1 m KOH, pH 13) and acidic (0.1 m HCl, pH 1) conditions for 24 h at 37 °C (see Supplementary Figs. 6 and 7). Even under these enforced settings we did not observe any pH-dependent isomerization processes, indicating that the manQ isomers 3 and 4 are fairly stable compounds.

Based on these experiments, we showed that the widely accepted β-homoallyl structure reported in literature for the non-canonical RNA nucleoside manQ is not correct. Instead, natural manQ is an α-allyl-mannoside (4, Fig. 1d). Its connectivity is therefore maximally different from the β-homoallyl-compound galQ (2, Fig. 1b). The elucidation of the correct manQ structure and the availability of synthetic material will be the basis for illuminating the biological functions of this hypermodified RNA nucleoside.

## Methods

### Isolation of total RNA from mouse livers

The murine livers for these experiments were kindly provided by Prof. Dr. Stylianos Michalakis. The frozen (−80 °C) livers were pooled and crushed into smaller pieces. The still-frozen pieces were divided into several samples for isolation of their total RNA. To this reason, 1 mL of TriReagent (Sigma Aldrich) was added per 50 mg of still-frozen liver tissue. Then, samples were homogenised with a tissue lyser (Schwingmühle MM400 from Retsch), first at 20 Hz for 4 min, then at 30 Hz for 2 min. Next, 200 µL chloroform per 50 mg of liver tissue were added, and the mixture was centrifugated for full phase separation (12,000 × *g*; 15 min; 4 °C). Of each sample, the aqueous (clear) upper phase was transferred to a new tube and mixed with 500 µL isopropanol per 50 mg of liver tissue. After overnight incubation at −20 °C, samples were centrifugated (21,130 × *g*; 30 min; 4 °C), and the supernatant was carefully removed from the pellets. 1 mL of ice-cold 75% ethanol was added to the pellets, and the samples were vortexed shortly. Again, samples were centrifugated (21,130 × *g*; 20 min; 4 °C), and the supernatant was carefully removed from the pellets. This ethanol washing step was repeated two more times. The resulting total RNA-pellets were first dried at room temperature and then dissolved in nuclease-free water for subsequent enzymatic digestion.

### Enzymatic digestion of total RNA to the nucleoside level

3 µg of mouse (or HEK 293T) total RNA were digested to the nucleoside level using the Nucleoside Digestion Mix (New England BioLabs). To this reason, a solution of 3 µg total RNA in 42.5 µL of nuclease-free water was prepared. 5 µL of the Nucleoside Digestion Mix Reaction Buffer (10x), and 2.5 µL of the Nucleoside Digestion Mix were added, and the mixture was incubated for 2 h at 37 °C. Samples were subsequently supplemented (spiked) with an appropriate amount (see below) of a synthetic manQ compound dissolved in nuclease-free water, or an equal volume of nuclease-free water (control samples). Finally, nuclease-free water was added to give a total sample volume of 100 µL, which therefore was independent of a particular compound concentration. Of note, samples can be stored after digestion and/or spiking for several days at −80 °C without altering the results of subsequent LC/MS analyses.

### UHPLC-MS/MS-based co-injection experiments

Co-injection experiments were performed by spiking equimolar (with respect to the natural manQ nucleoside present in the digested total RNA) amounts of heavy-atom-labelled standard to the sample. UHPLC-MS/MS analyses of digested RNA samples were performed using an Agilent 1290 UHPLC system equipped with an UV detector and an Agilent 6490 triple quadrupole mass spectrometer. All samples were filtrated directly before measurement using an AcroPrep Advance 96 filter plate 0.2 μm Supor from Pall Life Sciences. The UHPLC autosampler was cooled to 4 °C. The source-dependent parameters of the MS instrument were as follows: gas temperature 230 °C; gas flow 12 L/min (N2); nebuliser 40 psi; sheath gas heater 300 °C; sheath gas flow 6 L/min (N2); capillary voltage 2.500 V in the positive ion mode; capillary voltage −2.250 V in the negative ion mode; nozzle voltage 0 V. The fragmentor voltage was 380 V. Fragmentation was performed with a collision energy of 35 eV and a cell accelerator voltage of 5 V. A specific fragmentation pattern of m/z = 572.2 → 163.1, as depicted in Supplementary Fig. 1, was observed. Delta EMV was set to 500 (positive mode) and 800 (negative mode). Chromatography was performed using a Poroshell 120 SB-C8 column (Agilent, 2.7 μm, 2.1 mm × 150 mm) at 35 °C with a gradient of water and MeCN, each containing 0.0085% (v/v) formic acid, at a flow rate of 0.35 mL/min. The gradient was as follows: 0 → 8 min, 0 → 2% MeCN (v/v); 8 → 10.9 min, 2 → 3.85% MeCN; 10.9 → 11.3 min, 3.85 → 80% MeCN; 11.3 → 12.0 min, 80% MeCN; 12.0 → 12.3 min, 80 → 0% MeCN; and 12.3 → 14.0 min, 0% MeCN. No other peaks besides the expected ones of manQ, galQ and the spiked standard were detected when using the parameters given above. The identity of galQ was identified by spiking with synthetic galQ-standard (see Supplementary Fig. 2).

### Cell culture metabolic feeding experiments

To determine which hexose is incorporated into the natural xQ/manQ base as its sugar moiety, HEK 293T cells were cultured in different heavy RPMI media (see Supplementary Table 1). Each heavy RPMI medium was supplemented with two monosaccharides: One of the two monosaccharides was fully ^13^C-isotope-labelled, while the other hexose was non-labelled and used in these experiments as a suppressor of metabolic interconversion. Heavy RPMI media with the following hexose combinations were used:

- ^13^C_6_-d-mannose, d-glucose
- d-mannose, ^13^C_6_-d-glucose
- ^13^C_6_-d-galactose, d-Glucose
- d-galactose, ^13^C_6_-d-glucose

1.3 million HEK 293T cells were seeded on a p60 cell culture dish. Cultivation was performed for 18–21 h in RPMI medium at 37 °C and 5% CO_2_. Subsequently, the medium was removed and replaced with heavy RPMI medium. Incubation continued under the same conditions for 30, 60 and 180 min.

### Isolation of total RNA from HEK 293T cells

After incubation, the culture medium was removed. The cells were carefully detached from the cell culture dish using PBS and transferred to a 2 mL reaction tube. This was followed by pelleting for 1 min at 500 × *g* and 4 °C. The PBS was removed and the cell pellet immediately resuspended in 1 ml TriReagent (Sigma Aldrich). Samples taken after 30 min in labelled medium were incubated for 40 min, the 60 min- and 180 min-samples were incubated for 5 min. Following incubation, 200 μL of chloroform were added to the sample, which then was heavily vortexed and subsequently centrifuged for 15 min at 12,000 × *g* and 4 °C. The upper clear phase was transferred to a new 2 mL reaction tube. 500 μL of isopropanol were added to the transferred phase and mixed. Precipitation of the RNA was performed at −20 °C overnight. Following this, samples were directly pelleted for 30 min at 21,130 × *g* and 4 °C. The supernatant was carefully removed. Thereafter, 1 ml of 75% (v/v) cold ethanol (−20 °C) was added and the sample was centrifuged for 20 min at 21,130 × *g* and 4 °C. The ethanol washing step including the centrifugation was repeated two more times. The supernatant was removed and the pellet was dried at room temperature. Thereafter, the pellet was dissolved in nuclease-free water.

### LC-MS-analysis of total RNA from metabolically labelled HEK cells

HPLC-HESI-MS analysis of the enzymatically digested total RNA of HEK cells (see above) was performed on a Dionex Ultimate 3000 HPLC system coupled to a Thermo Fisher LTQ Orbitrap XL mass spectrometer. Samples were filtrated before the measurement using an AcroPrep Advance 96 filter plate 0.2 μm Supor from Pall Life Sciences. Nucleosides were separated with an Interchim Uptisphere120-3HDO C18 column whose temperature was maintained at 30 °C. Elution buffers were buffer X (2 mM NH_4_HCOO in H_2_O; pH 5.5) and buffer Y (2 mM NH_4_HCOO in H_2_O/MeCN 20/80 v/v; pH 5.5) with a flow rate of 0.15 mL/min. The gradient was as follows: 0 → 3.5 min, 0% Y; 3.5 → 4 min, 0 → 0.2% Y; 4 → 10 min, 0.2% Y; 10 → 50 min, 0.2 → 4.7% Y; 50 → 55 min, 4.7 → 60% Y; 55 → 57 min, 60 → 100% Y; 57 → 62 min, 100% Y. The chromatogram was recorded at 260 nm with a Dionex Ultimate 3000 Diode Array Detector, and the chromatographic eluent was directly injected into the ion source of the mass spectrometer without prior splitting. Ions were scanned in the positive polarity mode over a full-scan range of m/z = 210–800 with a resolution of 60,000. Parameters of the mass spectrometer were tuned with a freshly mixed solution of inosine (5 µM) in buffer X and set as follows: Capillary temperature 275 °C; APCI vaporizer temperature 100 °C; sheath gas flow 5.00; aux gas flow 21.0; sweep gas flow 1.00; source voltage 4.80 kV; capillary voltage 0 V; tube lens voltage 45.0 V; skimmer offset 0 V. The ion chromatograms of the compounds of interest were extracted from the total ion current (TIC) chromatogram with a mass range set to ±0.0050 u around the exact mass [M + H]^+^ of a compound. The peak areas in the extracted ion chromatograms of the heavy and corresponding light compound were integrated and the percentual ^13^C_6_-labelling of a compound within a sample was calculated.

### Synthetic procedures

Synthetic procedures and analytical characterisation of the compounds used in this study are provided in the supporting information.

### Reporting summary

Further information on research design is available in the Nature Research Reporting Summary linked to this article.

## Supplementary information

Supplementary Information

Reporting Summary

## Data Availability

The HPLC/MS and NMR data generated in this study are provided in the Supplementary Information. Raw data files are available from the corresponding author upon request.

## Code availability

For data collection with the TripleQuad LC-MS/MS system, MassHunter Workstation Software Version B.07.01/Build 7.1.524.0 was used. For data collection with the Orbitrap LC-MS-system, the programmes Xcalibur Version 2.1.0 SP1.1160, Chromeleon Version 6.80 SR13 Build 3818 (226197) and LTQ Tune Plus Version 2.5.5 SP2 were used. Analysis of raw data from the TripleQuad LC-MS/MS system was performed with the programme MassHunter Workstation Software Version B.07.01/Build 7.1.524.0. Analysis of raw data from the Orbitrap LC-MS-system was performed with Xcalibur Qual Browser Version 2.1.0 SP1.1160. For general data analysis, Origin 2018 64 bit version was used.
