# Supplementary material for: Synthesis and structure elucidation of the human tRNA nucleoside mannosyl-queuosine
Source: Nat Commun. 2021 Dec 8;12:7123. doi: 10.1038/s41467-021-27371-9 (PMC8654956; doi:10.1038/s41467-021-27371-9)
Supplement: Supplementary file 1 — Supplementary Information [file 41467_2021_27371_MOESM1_ESM.pdf]

Synthesis and Structure Elucidation of the Human tRNA Nucleoside Mannosyl-Queuosine

Supplementary Information

Markus Hillmeier[a], Mirko Wagner[a], Timm Ensfelder[a], Eva Korytiakova[a], Peter Thumbs[a], Markus Müller[a], and Thomas Carell[a,#]

a) Department Chemie, Ludwig-Maximilians-Universität München, Butenandtstraße 5-13, 81377 München

Correspondence should be addressed to:

Thomas Carell

E-Mail: thomas.carell@lmu.de

Telefone: +49 (0)89 218077755

Fax: +49 (0)89 218077756

Webpage: www.carellgroup.de

Supplementary Information - Table of Contents

1. LC-MS-based comparison of natural manQ from mouse liver with our synthetic manQ-compounds for structure elucidation 3

1.1 UHPLC-MS/MS (QQQ) based co-injection experiments for manQ structure elucidation 3

1.2 HPLC-MS (Orbitrap)-based repetition of co-injection experiments for manQ structure confirmation 4

1.3 Investigation of manQ stability towards isomerization 6

2. Metabolic feeding study to confirm mannose as the hexose-part of natural manQ 10

3. Chemical synthesis 11

3.1. Synthesis of glycosyl donor 5 11

3.2 Synthesis of ß-*homoallyl*-manQ (3) 17

3.3 Regioselective protection of 12 towards cyclopentene precursors 13-15 21

3.4 Synthesis of glycosyl donor 16 26

3.5 Synthesis of β-*allyl*-manQ (10), α-*homoallyl*-manQ (11), and of α-*allyl*-manQ (4) 31

4. NMR-spectra of important compounds 40

5. UHPLC-MS/MS chromatograms of co-injection experiments 74

6. Supplementary References 75

1. LC-MS-based comparison of natural manQ from mouse liver with our synthetic manQ-compounds for structure elucidation

1.1 UHPLC-MS/MS (QQQ) based co-injection experiments for manQ structure elucidation

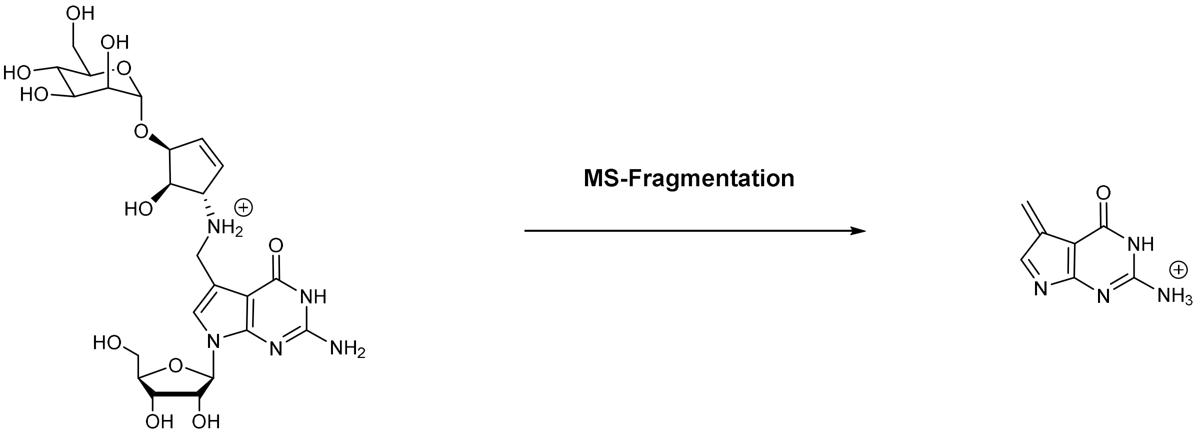

4, m/z = 572.2 **23**, m/z = 163.1

Supplementary Figure 1. Fragmentation pattern used for specific detection of manQ and galQ on the QQQ mass spectrometer. The fragmentation is depicted for natural manQ **4**, but occurs in a similar way for the precursor ions **2**, **3**, **10,** and **11**, giving the same daughter ion **23** with m/z = 163.1 and the likely structure depicted here.

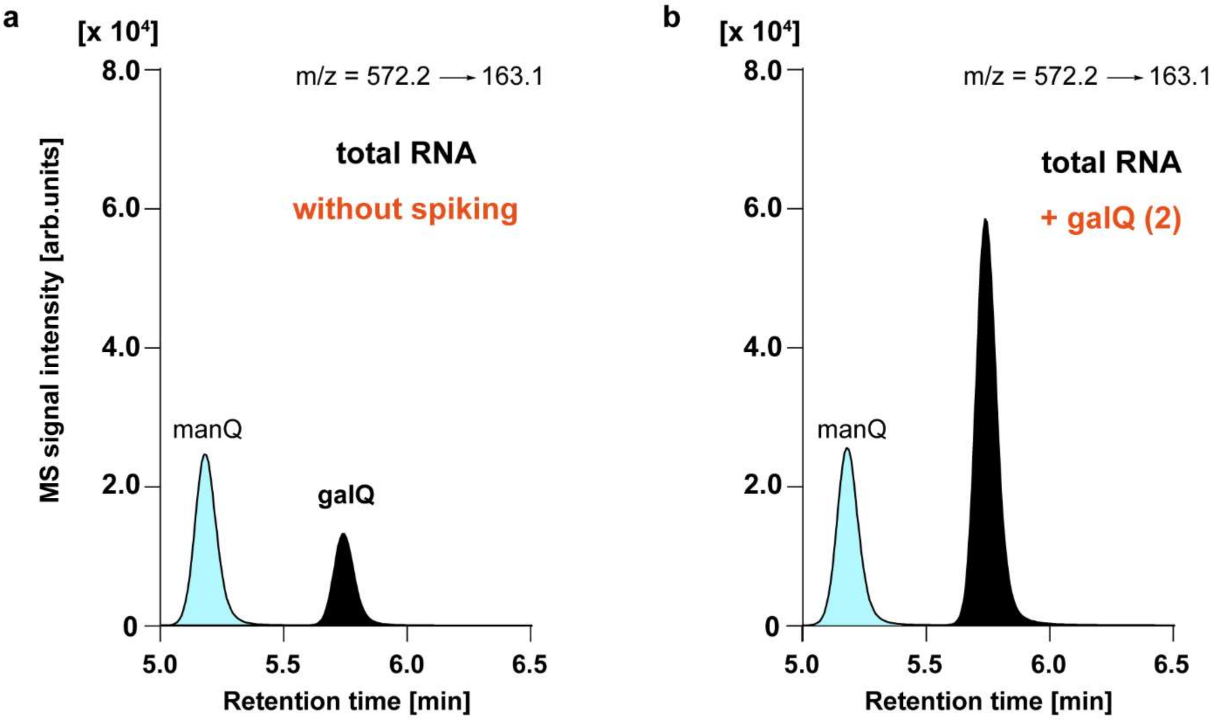

Supplementary Figure 2. UHPLC-MS/MS-based identification of the galQ-peak by co-injection. **a,** Digested total RNA from mouse tissue only (control); **b,** Digested total RNA from mouse tissue spiked with synthetic galQ as recently prepared by us[1] showed a complete signal overlap with the peak appearing at higher retention time, therefore identifying this peak as natural galQ.

1.2 HPLC-MS (Orbitrap)-based repetition of co-injection experiments for manQ structure confirmation

To double-check our results from the UHPLC-MS/MS experiments, we repeated the co-injection experiments on a second LC-MS system using a different HPLC separation column. To take into account the lower sensitivity of the Orbitrap system in comparison to the QQQ-system, the samples, containing the digested RNA (see Supplementary Information **1.2**) and the spiked synthetic material, were scaled accordingly. The ratios of digested RNA vs. amount of spiked material, however, were again about equimolar and therefore the same as for the UHPLC-MS/MS analyses. All samples were filtrated before measurement using an *AcroPrep Advance 96 filter plate 0.2 µm Supor* from *Pall Life Sciences*. The HPLC-HESI-MS analyses were performed on a *Dionex Ultimate 3000* HPLC system coupled to a *Thermo Fisher LTQ Orbitrap XL* mass spectrometer. Nucleosides were separated with an *Interchim Uptisphere120-3HDO C18* column whose temperature was maintained at 30 °C. Elution buffers were buffer X (2 mM NH_4HCOO in H_2O; pH 5.5) and buffer Y (2 mM NH_4HCOO in H_2O/MeCN 20/80 v/v; pH 5.5) and the gradient was as follows: 0→10 min, 0 % Y, 0.15 mL/min; 10→50 min, 0→5 % Y, 0.15 mL/min; 50→52 min, 5→75 % Y, 0.15 mL/min→0.20 mL/min; 52→55 min, 75 % Y, 0.20 mL/min. The chromatogram was recorded at 260 nm with a *Dionex Ultimate 3000 Diode Array Detector,* and the chromatographic eluent was directly injected into the ion source of the mass spectrometer without prior splitting. Ions were scanned in the positive polarity mode over a full-scan range of m/z = 225-2000 with a resolution of 100,000. Parameters of the mass spectrometer were tuned with a freshly mixed solution of inosine (5 µM) in buffer X and set as follows: Capillary temperature 275 °C; APCI vaporizer temperature 100 °C; sheath gas flow 5.00; aux gas flow 21.0; sweep gas flow 1.00; source voltage 4.80 kV; capillary voltage 0 V; tube lens voltage 45.0 V; skimmer offset 0 V. The ion chromatograms of the compounds of interest were extracted from the total ion current (TIC) chromatogram with a mass range set to +/- 0.0020 u around the exact mass $[M+H]^+$ of the compounds of interest.

As shown in **Supplementary Fig. 3**, these experiments again did NOT result in the appearance of a new peak, but resulted in a full signal overlap of the natural manQ compound from mouse liver and our synthetic α-*allyl*-manQ **4**. As expected from our UHPLC-MS/MS co-injection experiments, these results thereby confirm the identity of the natural manQ compound as α-*allyl*-manQ **4**.

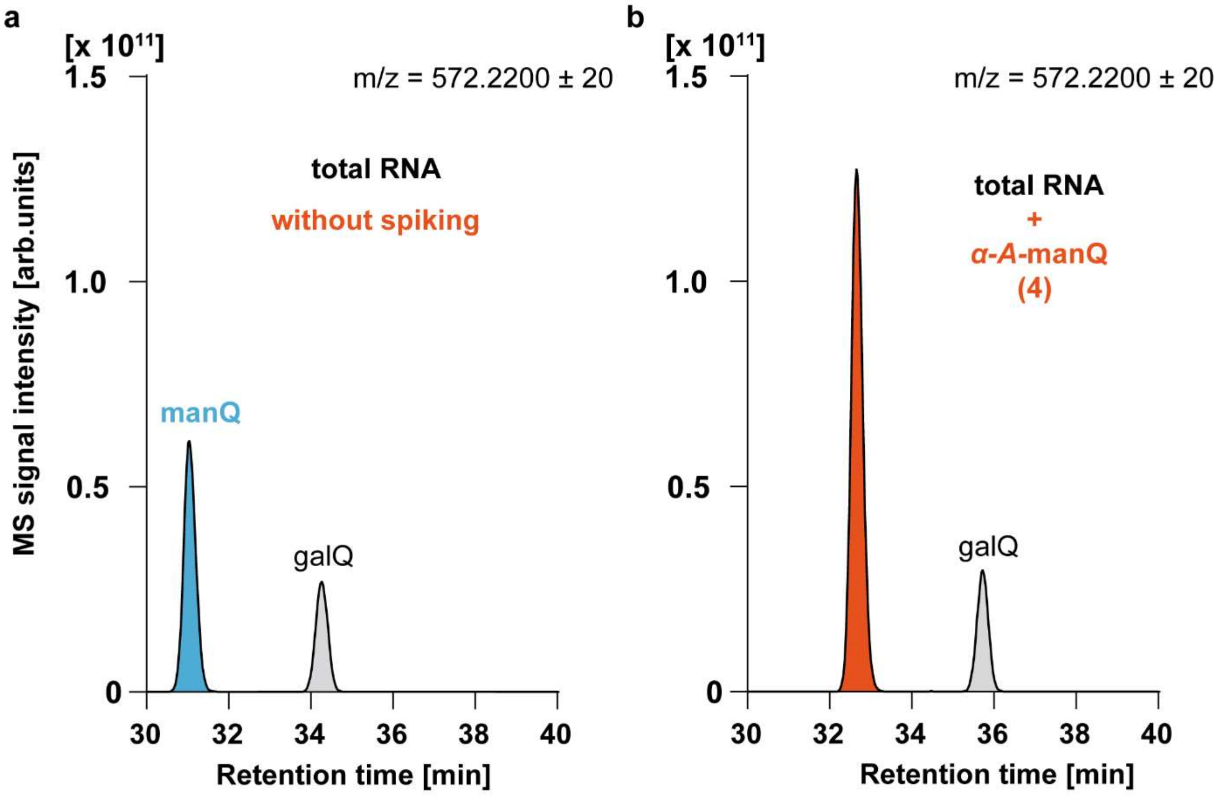

Supplementary Figure 3. Extracted ion chromatograms resulting from the co-injection of the synthetic manQ compound **4** with enzymatically digested total RNA from mouse liver analyzed by HPLC-MS: Co-injection of α-*allyl*-manQ **4** with digested total RNA from mouse liver shows a complete signal overlap of **4** and natural manQ, thereby leading to an increased signal intensity of manQ (**b**) in comparison to the control sample (**a**) without synthetic standard. This result confirms the findings from the UHPLC-MS/MS-experiments and shows again that our synthetic α-*allyl*-manQ **4** is identical to the naturally occurring manQ compound. Of note, the calculated mass of α-*allyl*-manQ **4** and of galQ **2** is 572.2199 for $[xQ + H]^+$. Shown here are extracted ion chromatograms with m/z = 572.2200±20, allowing for a maximal mass deviation of δ = 3.7 ppm only.

Of note, the proper HPLC-separation of the manQ-compounds on this system was more sensitive towards changes of gradient and column age than in case of the UHPLC-based separation with a *Poroshell 120 SB-C8* column. In rare cases, when the column had been used for a prolonged period of time, the peaks of synthetic α-*homoallyl*-manQ **11** and of the natural manQ compound α-*allyl*-manQ **4** were not properly separable any more.

1.3 Investigation of manQ stability towards isomerization

To investigate whether our RNA isolation conditions could lead to isomerization of the manQ-isomers, we subjected compounds **3**, **4**, **10,** and **11** to the *TriReagent*-isolation conditions described above. For this, *TriReagent* (1 mL, *Sigma Aldrich*) was added to a solution of each standard and vortexed for 5 min. Chloroform (200 µL) was added, the mixture was vortexed for 5 min and then kept at room temperature for 10 min. After centrifugation (12,000 g, 15 min, 4 °C), the aqueous phase was separated and isopropanol (500 µL) was added. The solution was stored at -20 °C overnight. The solvent was evaporated and the residue was dissolved in H_2O (500 µL). The aqueous phase was extracted with chloroform (3 x 200 µL) and the solvent was removed by lyophilization. The residue was taken up in H_2O and analyzed by UHPLC-MS/MS. The resulting chromatograms are shown in **Supplementary Figure 4**. None of the isomers did undergo isomerization under these conditions.

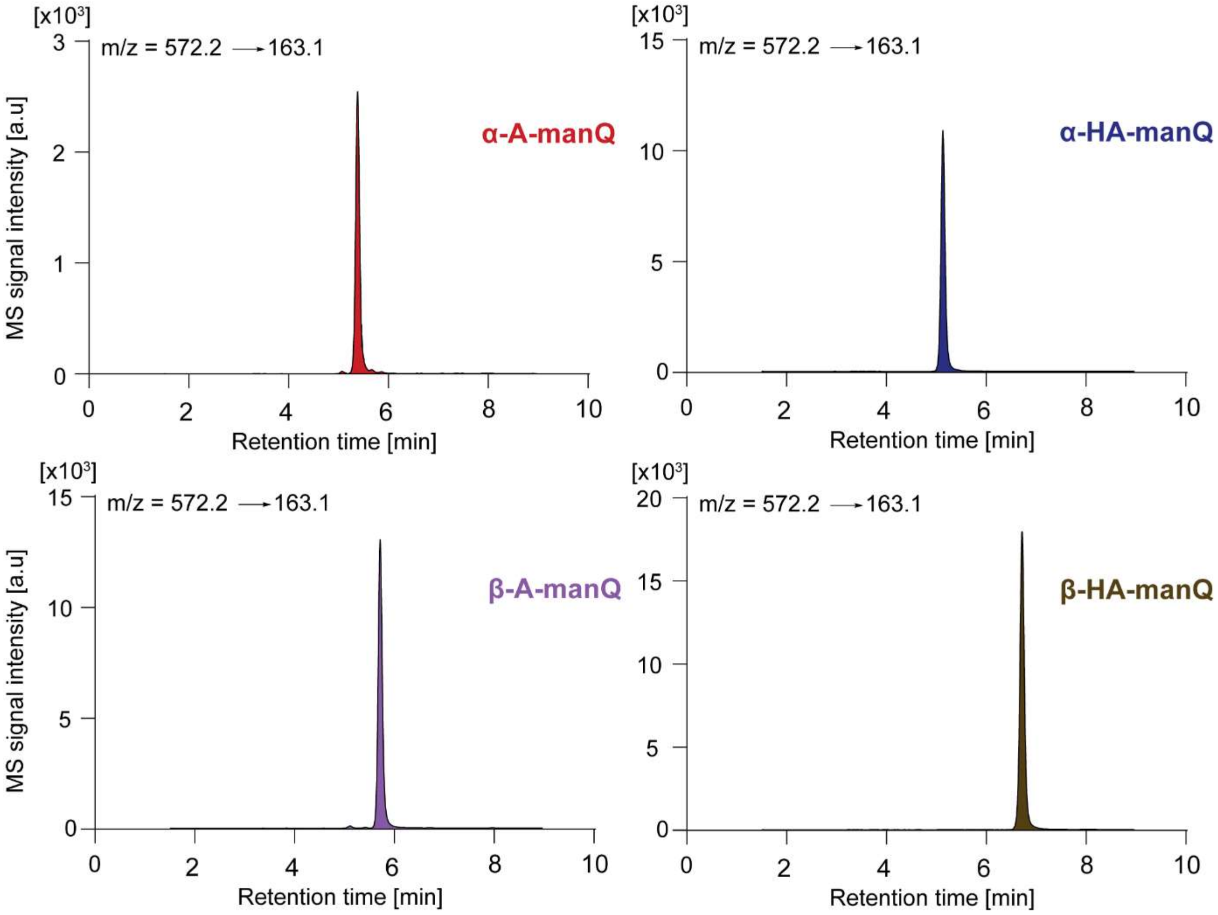

Supplementary Figure 4. Investigation of manQ stability towards isomerization under our RNA isolation conditions. Depicted are the UHPLC-MS/MS chromatograms of the stability tests. After *TriReagent*-treatment, all four isomers still give the expected signal at their characteristic retention time with no additional peaks of an isomerization product appearing.

Next, we checked whether our RNA digestion conditions could lead to isomerization of the manQ compounds. To this reason, a solution of the manQ nucleoside standard **3**, **4**, **10**, or **11** in nuclease-free water (42.5 µL) was prepared. 5 µL of *Nucleoside Digestion Mix Reaction Buffer (10x, New England BioLabs)* and 2.5 µL of *Nucleoside Digestion Mix* (*New England BioLabs)* were added, and the mixture was incubated for 2 h at 37 °C. The sample was then analyzed by UHPLC-MS/MS. The results for all manQ isomers are shown in **Supplementary Figure 5**. Under the conditions tested, no isomerization of any of the compounds **3**, **4**, **10** or **11** occurred.

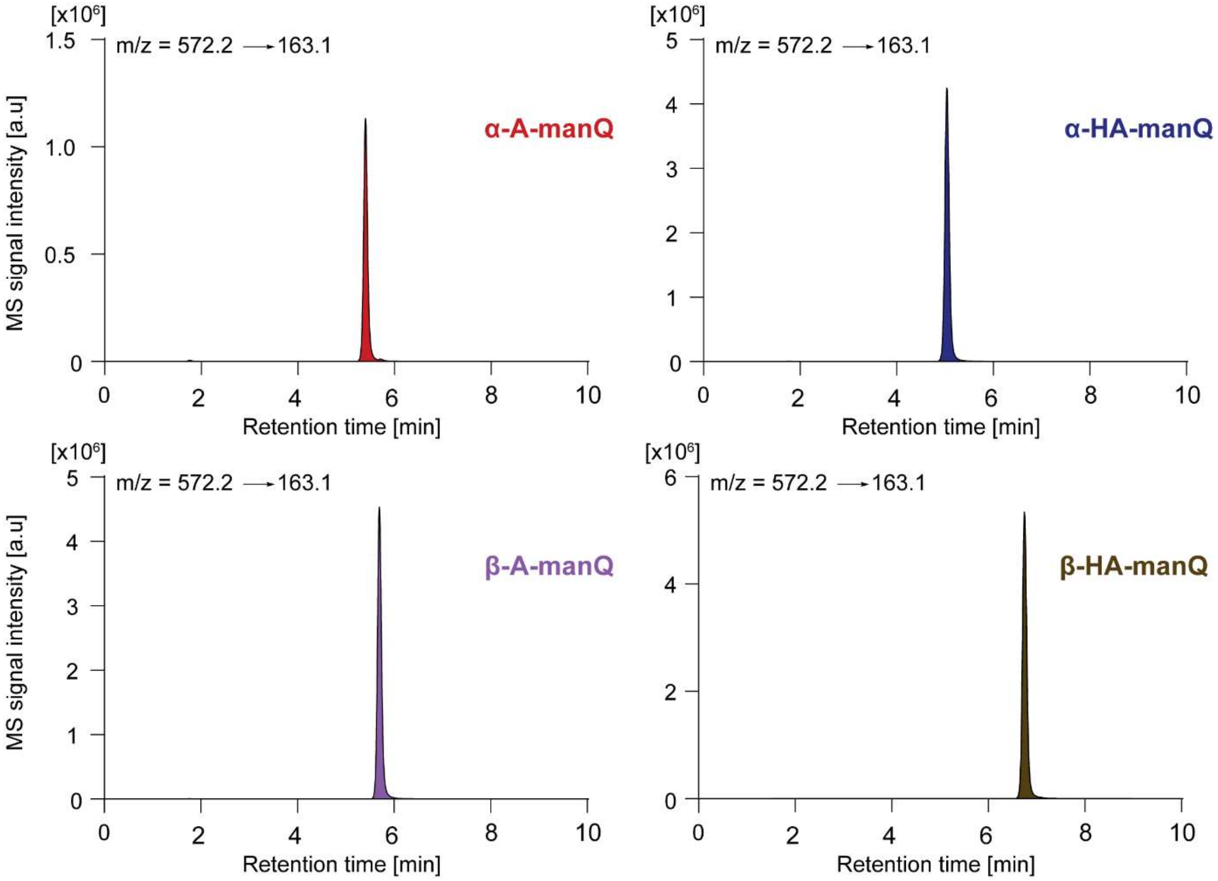

Supplementary Figure 5. Investigation of manQ stability towards isomerization under our RNA digestion conditions. Depicted are the UHPLC-MS/MS chromatograms of the stability tests. After treatment, all four isomers still give the expected signal at their characteristic retention time with no additional peaks of an isomerization product appearing.

We additionally tested whether α-*allyl*-manQ **4** and β-*homoallyl*-manQ **3** and could isomerize into each other under certain pH-conditions. Theoretically, this isomerization might occur by an attack of the neighboring hydroxyl group at the cyclopentene unit, leading to a S_N2-type rearrangement reaction. To investigate this possibility, we subjected compounds **3** and **4** to neutral (H_2O, pH 7), acidic (0.1 M HCl, pH 1) and basic conditions (0.1 M KOH, pH 13) and kept the solutions for 24 h at 37 °C. We then neutralized the solutions with KOH or HCl,

respectively, and analyzed the samples by UHPLC-MS/MS. To ensure similar conditions, 0.1 M KCl was added to the neutral samples. The results are depicted in **Supplementary Figures 6** (α-*allyl*-manQ) and **7** (β-*homoallyl*-manQ). In no case any formation of an isomerization product was detectable.

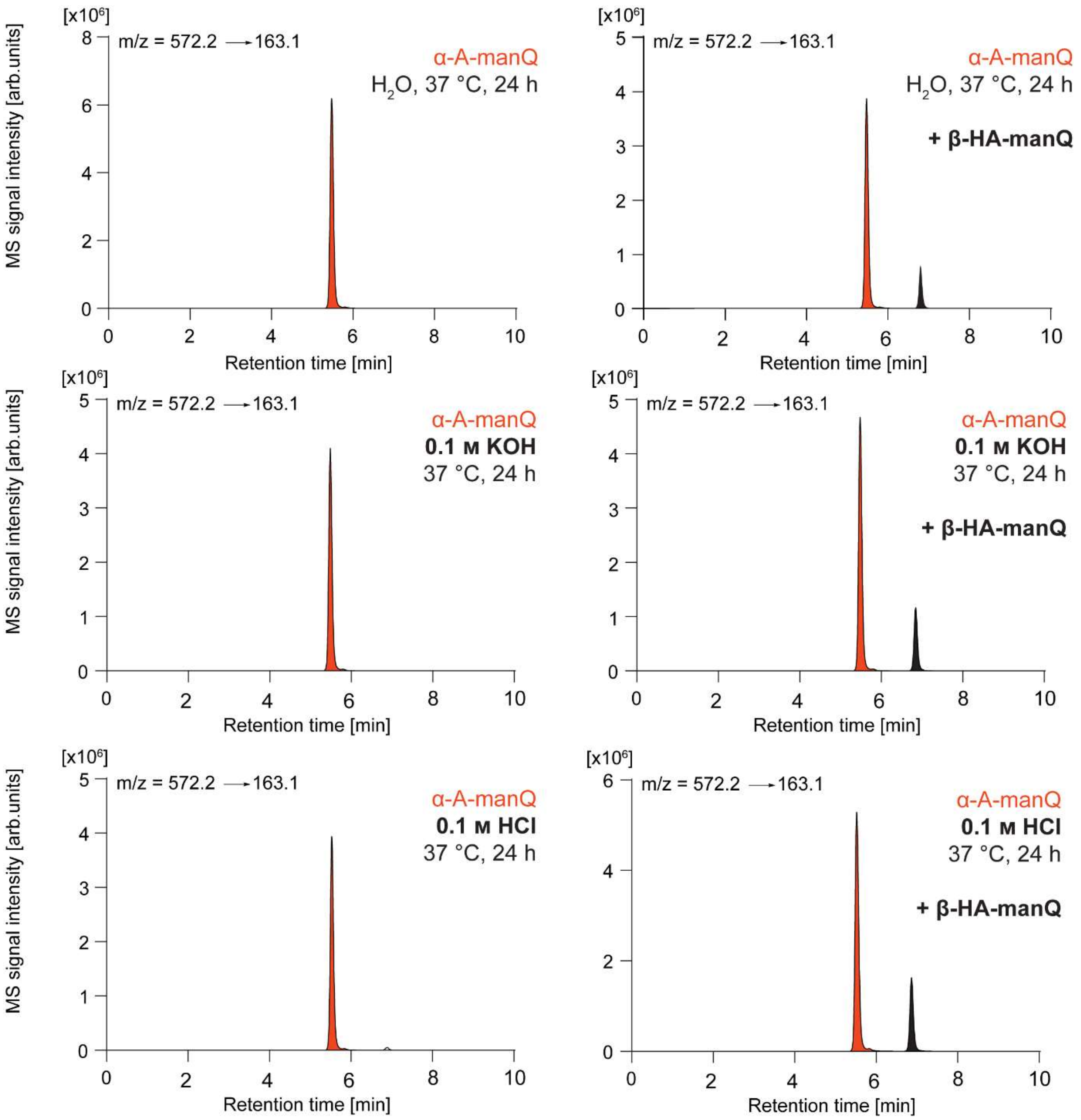

Supplementary Figure 6. Stability of α-*allyl*-manQ **4** (α-A-manQ) towards isomerization into β-*homoallyl*-manQ **3** (β-HA-manQ) under neutral, basic, and acidic conditions. Left side: UHPLC-MS/MS chromatograms obtained after the pH-treatment show no additional signals next to the expected one of manQ **4**; right side: chromatograms of treated samples spiked with compound **3** to indicate the retention time of this putative isomerization product. The data show no sign of an isomerization of compound **4** towards **3**.

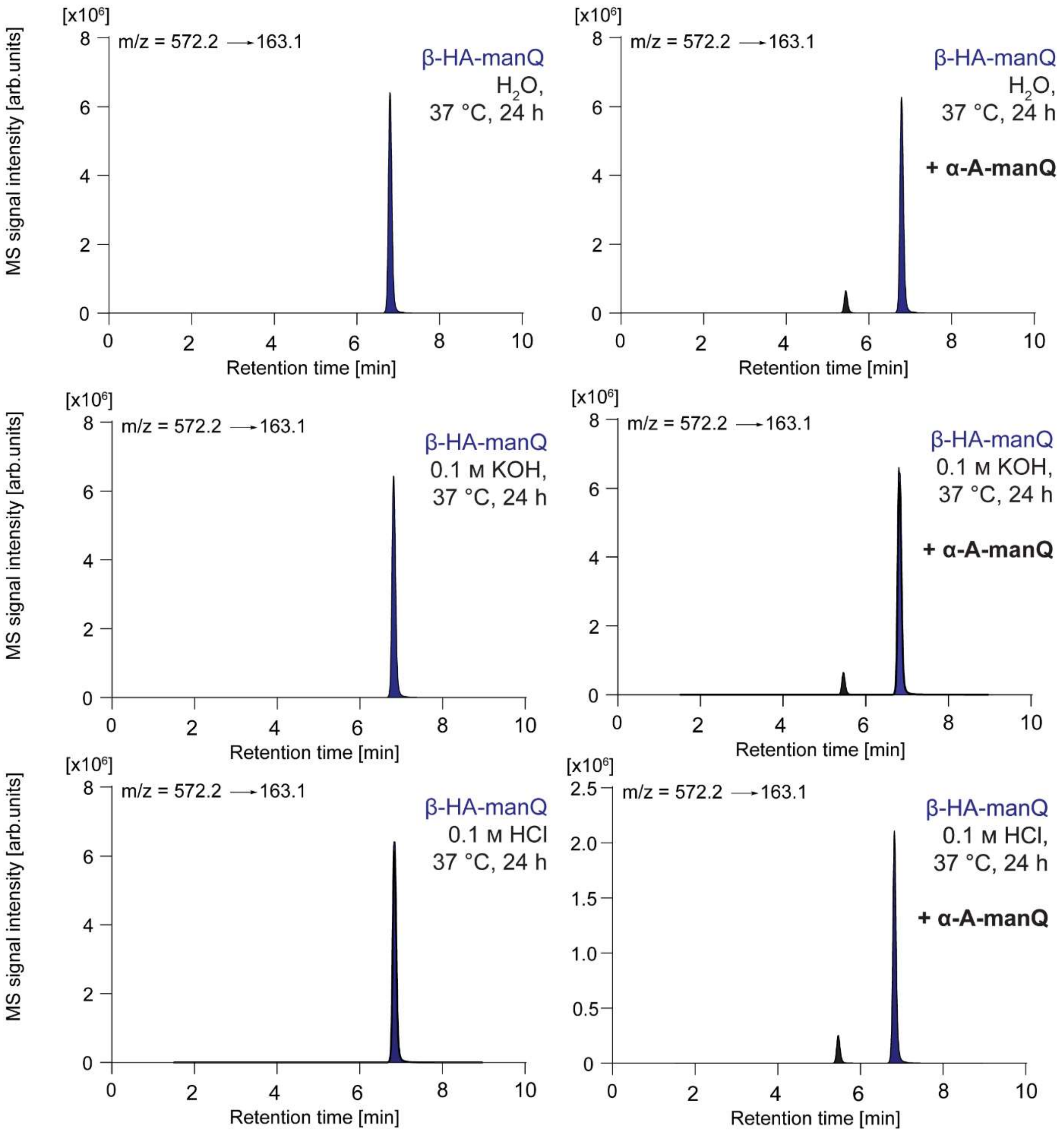

Supplementary Figure 7. Stability of β-*homoallyl*-manQ **3** (β-HA-manQ) towards isomerization into α-*allyl*-manQ **4** (α-A-manQ) under neutral, basic and acidic conditions. Left side: UHPLC-MS/MS chromatograms obtained after the pH-treatment show no additional signals next to the expected one of manQ **3**; right side: chromatograms of treated samples spiked with compound **4** to indicate the retention time of this putative isomerization product. The data show no sign of an isomerization of compound **3** towards **4**.

2. Metabolic feeding study to confirm mannose as the hexose-part of natural manQ

Supplementary Table 1: Composition of the used media. Light RPMI medium corresponds to regular RPMI medium. For Heavy RPMI medium, a glucose free RPMI mixture was used (w/o g.: without glucose). A combination of either mannose and glucose or galactose and glucose was added to 11 mM final concentration each. One of the two hexoses was fully ^{13}C-labelled in each mixture.

**Medium**	**Composition**
Light RPMI medium	88 % (v/v) RPMI; 10 % (v/v) FBS; 1 % (v/v) Pen-Strep; 1 % Ala-Gln
Heavy RPMI medium gal/glc	88 % (v/v) RPMI (w/o g.); 10 % (v/v) FBS; 1 % (v/v) Pen-Strep; 1 % Ala-Gln; 11 mM D-galactose(-$^{13}C_6$); 11 mM D-glucose(-$^{13}C_6$)
Heavy RPMI medium man/glc	88 % (v/v) RPMI (w/o g.); 10 % (v/v) FBS; 1 % (v/v) Pen-Strep; 1 % Ala-Gln; 11 mM D-mannose(-$^{13}C_6$); 11 mM D-glucose(-$^{13}C_6$)

3. Chemical synthesis

3.1. Synthesis of glycosyl donor 5

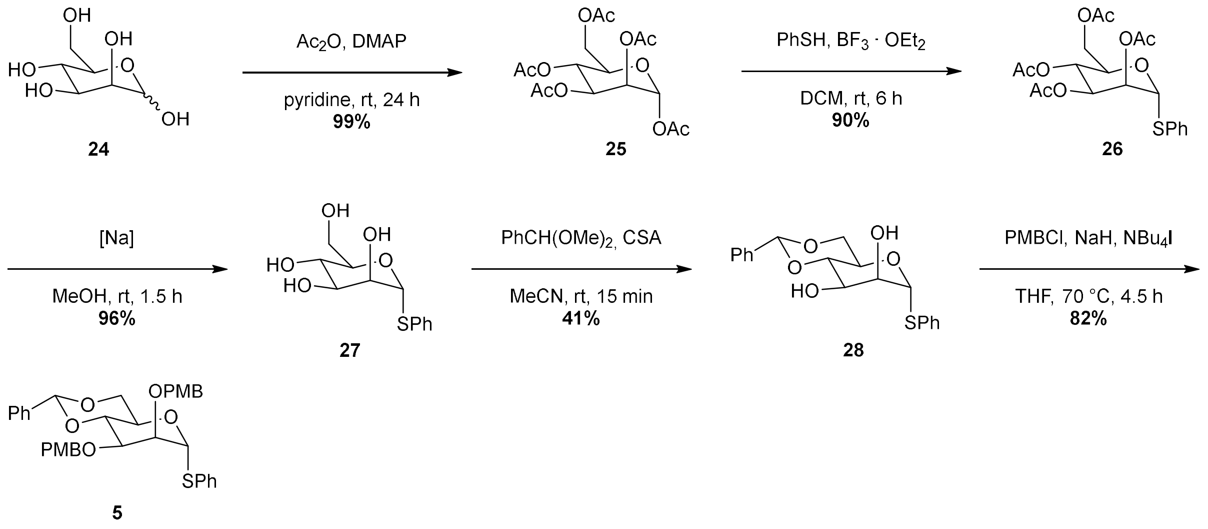

Supplementary Figure 8: Synthesis of glycosyl donor **5** in 5 steps starting from D-mannose (29 % overall yield)

1,2,3,4,6-penta-*O*-acetyl-α-D-mannopyranose (25)[2]

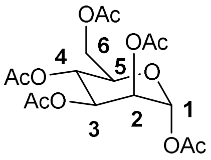

D-Mannose (5.00 g, 27.8 mmol, 1.00 eq) was dissolved in pyridine (104 mL). Ac_2O (78.0 mL, 416 mmol, 30.0 eq) and DMAP (339 mg, 2.78 mmol, 0.10 eq) were added at 0 °C. The reaction was stirred at room temperature for 24 h, which resulted in an orange solution. After evaporation of the solvent *in vacuo* the residue was taken up in EtOAc (100 mL) and washed with aqueous HCl (1 M, 100 mL), H_2O (2 x 100 mL) and brine (100 mL). After evaporation of the solvent *in vacuo* the product (10.8 g, 27.7 mmol, 99%) was obtained as yellow viscous syrup. (only α-anomer).

R_f (EtOAc/*iso*-hexane 1:1) = 0.50.

^{1}H-NMR ($CDCl_3$, 400 MHz): δ = 6.08 (d, *J* = 1.9 Hz, 1H, C1H), 5.35 (m, 2H, C3H, C4H), 5.26 (td, J_1 = 2.2 Hz, J_2 = 0.7 Hz, 1H, C2H), 4.28 (dd, J_1 = 12.4, J_2 = 4.9 Hz, 1H, C6Ha), 4.13-4.02 (m, 2H, C5H, C6Hb 2.18 (s, 3 H, CO_2CH_3), 2.17 (s, 3 H, CO_2CH_3), 2.09 (s, 3 H, CO_2CH_3), 2.05 (s, 3 H, CO_2CH_3), 2.01 (s, 3 H, CO_2CH_3) ppm.

^{13}C-NMR ($CDCl_3$, 100 MHz): δ = 170.8 (CO_2CH_3), 170.2 (CO_2CH_3), 169.9 (CO_2CH_3), 169.7 (CO_2CH_3), 168.2 (CO_2CH_3), 90.7 (C1), 70.7 (C5), 68.8 (C3), 68.4 (C2), 65.6 (C4), 62.2 (C6), 21.0 (CO_2CH_3), 20.9 (CO_2CH_3), 20.9 (CO_2CH_3), 20.8 (CO_2CH_3), 20.8 (CO_2CH_3) ppm.

HRMS (ESI^+) m/z calc. for $C_{16}H_{22}O_{11}Na$, $[M+Na]^+$: 413.1060; found: 413.1056.

NMR-spectra in accordance with literature.

2,3,4,6-penta-*O*-acetyl-1-thiophenyl-α-D-mannopyranoside (26)[3]

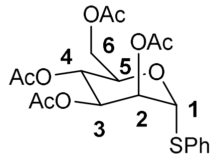

25 (5.00 g, 12.8 mmol, 1.00 eq) was dissolved in DCM (25 mL). Thiophenol (2.65 mL, 2.82 g, 25.6 mmol, 2.00 eq) and $BF_3 \cdot OEt_2$ (4.80 mL, 5.45 g 34.8 mmol, 3.00 eq) were added at 0 °C. The reaction was stirred at room temperature for 6 h. Saturated aqueous $NaHCO_3$-solution (20 mL) was added and the reaction mixture was stirred for 15 min (until no more gas evolution was observable). The phases were separated and the aqueous phase was re-extracted with DCM (2 x 30 mL). The combined organic phases were washed with brine (20 mL), dried over Na_2SO_4, filtered and the solvent was removed *in vacuo*. Column chromatographic purification (EtOAc/*iso*-hexane 1:3) afforded the product (5.09 g, 11.6 mmol, 90%) as yellow syrup.

R_f (EtOAc/*iso*-hexane 1:1) = 0.76.

^{1}H-NMR ($CDCl_3$, 600 MHz): δ = 7.44 (m, 2H, arom. CH), 7.26 (m with solvent peak, 3H, arom. CH), 5.46 (m, 2H, C1H, C2H), 5.28 (m, 2H, C3H, C4H), 4.50 (m, 1H, C5H), 4.26 (dd, J_1 = 12.2, J_2 = 5.9 Hz, 1H, C6Ha), 4.07 (m, 1H, C6Hb), 2.11 (s, 3H, CO_2CH_3), 2.03 (s, 3H, CO_2CH_3), 2.01 (s, 3H, CO_2CH_3), 1.97 (s, 3H, CO_2CH_3) ppm.

^{13}C-NMR ($CDCl_3$, 125 MHz): δ = 170.7 (CO_2CH_3), 170.0 (CO_2CH_3), 169.9 (CO_2CH_3), 169.9 (CO_2CH_3), 132.7 (arom. C), 132.2 (arom. C), 129.3 (arom. C), 128.2 (arom. C), 85.5 (C1), 71.0 (C2), 69.6 (C5), 69.5 (C3/C4), 66.5 (C3/C4), 62.6 (C6), 21.0 (CO_2CH_3), 20.8 (CO_2CH_3), 20.8 (CO_2CH_3), 20.8 (CO_2CH_3) ppm.

HRMS (ESI^+) m/z calc. for $C_{20}H_{24}NaO_9S$, $[M+Na]^+$: 463.1039; found: 463.1037.

Analytical data in accordance with literature.

1-thiophenyl-α-D-mannopyranoside (27)[3]

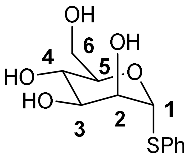

26 (5.09 g, 11.6 mmol) was dissolved in MeOH (50 mL). A catalytic amount of sodium was added and the reaction mixture was stirred for 1.5 h at room temperature resulting in a suspension. The reaction was neutralized with AcOH, upon which the precipitate redissolved. Evaporation of the solvent gave the product (2.69 g, 11.1 mmol, 96%) as colorless foam.

R_f (EtOAc/*iso*-hexane 1:1) = 0.03.

^{1}H-NMR (methanol-D_4, 600 MHz): δ = 7.55-7.52 (m, 2H, aromatic CH), 7.34-7.26 (m, 3H, aromatic CH), 5.43 (d, J = 1.6 Hz, 1H, C1H), 4.09 (dd, J_1 = 3.2 Hz, J_2 = 1.6 Hz, 1H, C2H), 4.04 (ddd, J = 9.4, 5.4, 2.5 Hz, 1H, C5H), 3.83 (dd, J_1 = 12.0, J_2 = 2.5 Hz, 1H, C6Ha), 3.77 (dd, J = 12.0, 5.5 Hz, 1H, C6Hb), 3.73 (t, J_1 = J_2 = 9.4 Hz, 1H, C4H), 3.69 (dd, J_1 = 9.4 Hz, J_2 = 3.1 Hz, 1H, C3H) ppm.

^{13}C-NMR (methanol-D_4, 125 MHz): δ = 135.9 (aromatic C), 133.0 (aromatic C), 130.0 (aromatic C), 128.5 (aromatic C), 90.5 (C1), 75.7 (C5), 73.8 (C2), 73.2 (C3), 68.7 (C4), 62.6 (C6) ppm.

HRMS (ESI^-) m/z calc. for $C_{12}H_{15}O_5S$, $[M-H]^-$: 271.0646; found: 271.0644.

Analytical data in accordance with literature.

4,6-O-benzylidene-1-thiophenyl-α-D-mannopyranoside (28)[4]

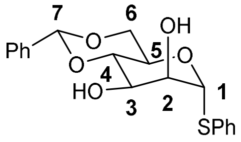

27 (1.00 g, 3.67 mmol, 1.00 eq) was dissolved in MeCN (15 mL). CSA (213 mg, 0.918 mmol, 0.25 eq) and $PhCH(OMe)_2$ (0.61 mL, 615 mg, 4.04 mmol, 1.10 eq) were added. The reaction was stirred at room temperature for 15 min, after which a solid precipitated. DCM (50 mL) and H_2O (50 mL) were added and the phases were separated. The aqueous phase was reextracted with DCM (2 x 30 mL) and the combined organic phases were dried over Na_2SO_4, filtered and the solvent was evaporated. Column chromatographic purification (MeOH/DCM 1:200) gave the product (540 mg, 1.50 mm0l, 41%) as colorless solid.

$\mathbf{R_f}$ (MeOH/DCM 1:100) = 0.24.

^{1}H-NMR (methanol-D_4, 600 MHz): δ = 7.53 (m, 4H, arom. CH), 7.34 (m, 6H, arom. CH), 5.64 (s, 1H, C7H), 5.50 (d, J = 1.2 Hz, 1H, C1H), 4.25 (td, , J_1 = 9.7 Hz, J_2 = 4.9 Hz, 1H, C5H), 4.19 (dd, J_1 = 3.3 Hz, J_2 = 1.4 Hz, 1H, C2H), 4.14 (dd, , J_1 = 10.2 Hz, J_2 = 4.9 Hz, 1H, C6Ha), 4.04 (t, J = 9.5 Hz, 1H, C4H), 3.96 (dd, J_1 = 9.9 Hz, J_2 = 3.3 Hz, 1H, C3Hw), 3.85 (t, J = 10.4 Hz, 1H, C6Hb) ppm.

^{13}C-NMR (methanol-D_4, 600 MHz): δ = 137.4, 132.9, 130.2, 130.1, 129.1, 128.7, 127.5, 125.8 (all aromatic C), 103.4 (C7), 89.4 (C1), 72.9 (C2), 72.8 (C4), 68.6 (C3), 68.2 (C6), 65.1 (C5) ppm.

HRMS (ESI^+) m/z calc. for $C_{19}H_{21}O_5S$, $[M+H]^+$: 361.1104 ; found: 361.1106.

Analytical data in accordance with literature.

4,6-O-Benzylidene-2,3-di-*O*-(*para*-methoxybenzyl)-1-thiophenyl-α-D-mannopyranoside (5)[5]

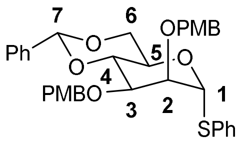

28 (540 mg, 1.50 mmol, 1.00 eq) was dissolved in THF (11 mL). NaH (240 mg, 6.00 mmol, 4.00 eq) was added at 0 °C. Next, PMB-Cl (0.81 mL, 940 mg, 6.00 mmol, 4.00 eq) and NBu_4I (28.0 mg, 0.075 mmol, 0.05 eq) were added. The reaction was stirred at 70 °C for 4.5 h before being cooled to room temperature. Saturated aqueous $NaHCO_3$-solution (20 mL) was added and the phases were separated. The aqueous phase was re-extracted with EtOAc (3 x 50 mL). The combined organic phases were dried over Na_2SO_4, filtered and the solvent was removed *in vacuo*. Column chromatographic purification (EtOAc/*iso*-hexane 1:2) afforded the product (739 mg, 1.23 mmol, 82%) as colorless syrup.

R_f (MeOH/DCM 1:100) = 0.24.

^{1}H-NMR ($CDCl_3$, 400 MHz): δ = 7.33 (m, 14H, arom. CH), 6.85 (m, 4H, arom. CH), 5.62 (s, 1H, C7H), 5.43 (d, *J* = 1.4 Hz, 1H, C1H), 5.62 (s, 1H, C1H), 5.43 (d, *J* = 1.4 Hz, 1H), 4.72 (d, *J* = 11.8 Hz, 1H, CH_2), 4.63 (s, 2H, CH_2), 4.56 (d, *J* = 11.8 Hz, 1H, CH_2), 4.23 (m, 3H, C3H, C5H, C6Ha), 3.97 (dd, J_1 = 3.3 Hz, J_2 = 1.4 Hz, 1H, C2H), 3.89 (m, 2H, C4H, C6Hb), 3.80 (OCH_3), 3.79 (OCH_3) ppm.

^{13}C-NMR ($CDCl_3$, 100 MHz): δ = 159.4, 159.3, 137.7, 134.0, 131.7, 130.6, 130.0, 129.9, 129.5, 129.2, 129.0, 128.3, 127.7, 126.2, 114.0, 113.9 (all aromatic C), 101.6 (C7), 87.3 (C1), 79.1 (C3), 77.6 (C2), 75.9 (C4), 72.8 (CH_2), 72.8 (CH_2), 68.6 (C6), 65.6 (C5), 55.4 (2C, OCH_3) ppm.

HRMS (ESI^+) m/z calc. for $C_{35}H_{36}O_7SNa$, $[M+Na]^+$: 623.2079; found: 623.2076.

Analytical data in accordance with literature.

3.2 Synthesis of ß-*homoallyl*-manQ (3)

(9H-fluoren-9-yl)methyl((1S,4S,5R)-5-(((2R,4aR,6R,7S,8S,8aR)-7,8-bis((4-methoxy-benzyl)oxy)-2-phenylhexahydro-pyrano[3,2-d][1,3]dioxin-6-yl)oxy)-4-((tert-butyl-dimethylsilyl)oxy)cyclopent-2-en-1-yl)carbamate (7)

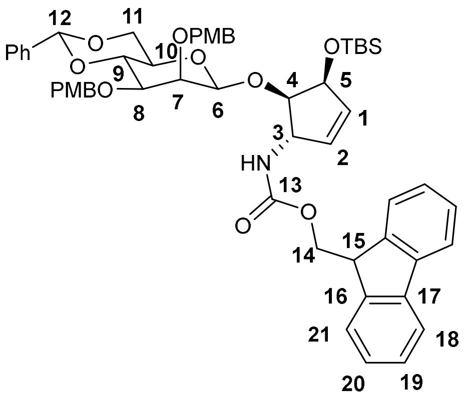

Cyclopentene **6** (100 mg, 219 µmol, 1.00 eq) was dissolved in DCM (2.5 mL) and stirred with 3 Å molsieves for 15 min (solution **A**). Glycosyl donor **5** (131 mg, 219 µmol, 1.00 eq) and DTBMP (135 mg, 656 µmol, 3.00 eq) were dissolved in DCM (2.5 mL) and stirred with 3 Å molsieves for 15 min (solution **B**). AgOTf (168 mg, 656 µmol, 3.00 eq) was dissolved in DCM (2.5 mL) and 3 Å molsieves were added. The reaction was cooled to -78 °C and stirred at that temperature for 15 min. A solution of PhSCl (546 µmol, 2.50 eq) in DCM (2.7 mL) was added and the reaction mixture was stirred at -78 °C for 5 min. Next, solution **B** was added and the reaction was stirred at -78 °C for 10 min. Then solution **A** was added and the reaction was stirred at -78 °C for 2 h. Saturated aqueous $NaHCO_3$-solution (20 mL) was added and the mixture was warmed to room temperature. After filtration, the phases were separated and the aqueous phase was re-extracted with DCM (3 x 20 mL). The combined organic phases were dried over Na_2SO_4, filtered and the solvent was removed *in vacuo*. Column chromatographic purification (EtOAc/*iso*-hexane 1:5 → 1:3) gave the product (136 mg, 142 µmol, 62%) as colorless solid.

$\mathbf{R_f}$ (EtOAc/*iso*-hexane 1:3) = 0.12.

^{1}H-NMR (600 MHz, $CDCl_3$, depicted in section 4 below): δ = 7.75 (d, *J* = 7.7 Hz, 2H, C21H), 7.56 (d, *J* = 7.4 Hz, 1H, C18H), 7.49 (m, 2H, C120H), 7.36 (m, 7H, arom. CH), 7.29 (m, 2H, C19H), 7.14 (d, *J* = 8.2 Hz, 2H, arom. CH), 6.83 (m, 2H, arom. CH), 6.79 (d, *J* = 8.4 Hz, 2H, arom. CH), 5.93 (m, 1H, C1H), 5.82 (d, *J* = 6.2 Hz, 1H, C2H), 5.59 (s, 1H, C12H), 4.83 (m, 4H, C3H, C6H (**$^1J_{C\text{-}H}$ = 158 Hz**), CH_2), 4.51 (m, 6H, C13H, C5H, 2 x CH_2), 4.25 (dd, , J_1 = 10.3 Hz, J_2 = 4.8 Hz, 1H, C11Ha), 4.20 (t, , *J* = 6.7 Hz, 1H, C14H), 4.11 (m, 1H, C9H), 3.96 (m, 2H, C4H, C7H), 3.86 (t, *J* = 10.3 Hz, 1H, C11Hb), 3.78 (s, 3H, OCH_3), 3.77 (s, 3H, OCH_3), 3.53 (d, , *J* = 9.8 Hz, 1H, C8H), 3.24 (m, 1H, C10H), 0.87 (s, 9H, $SiC(CH_3)_3$), 0.07 (s, 6H, $SiCH_3$) ppm.

^{13}C-NMR (150 MHz, $CDCl_3$, depicted in section 4 below): δ = 159.2 (arom. C), 155.8 (C13), 143.9 (C17), 141.5 (C16), 134.6 (C2), 134.3 (C1), 130.41 (arom. C), 129.2 (arom. C), 128.9 (arom. C), 128.3 (arom. C), 127.9 (arom. C), 127.2 (C19), 126.2 (C20), 125.0 (C18), 120.1 (C21), 113.8

(arom. C), 113.6 (arom. C), 101.5 (C12), 101.1 (C6), 82.8 (C4), 78.7 (C9), 77.6 (C8), 75.3 (C7), 74.4 (OCH_2), 74.3 (C5), 71.9 (OCH_2), 68.8 (C11), 67.8 (C10), 66.7 (C14), 59.0 (C3), 55.4 (OCH_3), 55.4 (OCH_3), 47.4 (C15), 26.1 ($SiC(CH_3)_3$), -4.2 ($SiCH_3$), -4.4 ($SiCH_3$) ppm.

HRMS (ESI^+) m/z calc. for $C_{55}H_{67}N_2O_{11}Si$, $[M+NH_4]^+$: 959.4509; found: 959.4524.

β-*homoallyl*-mannosyl-queuosine (3)

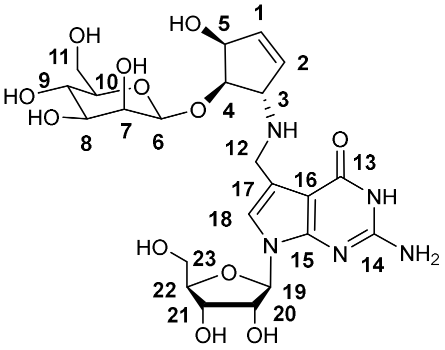

12 (23 mg, 24.0 µmol was dissolved in 10% $HNMe_2$ in THF (1 mL). The reaction was stirred for 1 h at room temperature before the solvent was removed *in vacuo*. The resulting yellow solid was washed with hexane to afford the product as colorless solid. The resulting Fmoc-deprotected derivative (14.9 mg, 20.6 µmol, 1.00 eq) was dissolved in MeOH (0.5 mL) and **13** (14.6 mg, 20.6 µmol, 1.00 eq) and AcOH (1 µL) were added. The reaction was stirred at rt for 5 h before it was cooled to 0 °C and $NaBH_4$ (2.02 mg, 53.6 µmol, 2.60 eq) was added. The reaction was stirred at 0 °C for 1 h. Then H_2O was added and the solvent was evaporated. The residue was dissolved in MeOH (1 mL) and NaOMe (27.0 mg, 0.5 mmol) was added. The reaction mixture was stirred at room temperature for 5 h, after which LC-MS indicated deprotection of all ester type protecting groups. The solution was neutralized with DOWEX-H^+-resin, then filtered and the solvent was evaporated. The residue was taken up in DCM (0.9 mL) and TFA (0.1 mL) was added at 0 °C. The reaction was stirred at 0 °C for 20 min, then saturated aqueous $NaHCO_3$-solution was added (1 mL). The mixture was evaporated and the residue was taken up in pyridine (1.5 mL) and HF · pyridine (7.5 eq) was added in a plastic falcon. The reaction was stirred for 18 h at room temperature before TMSOMe (15.0 eq) was added at 0 °C. After stirring the reaction for 1 h at 0 °C, the solvent was evaporated *in vacuo*. The residue was dissolved in H_2O (5 mL), filtered and subjected to preparative HPLC-purification (0-8% buffer B, 45 min, R_t = 29.2 min) to afford β-*homoallyl*-manQ as a colorless solid (2.99 mg, 5.24 µmol, 22% over 5 steps).

RP-HPLC: R_t (C18-column, 0-8% buffer B, 45 min) = 29.2 min.

^{1}H-NMR (methanol-D_4, 600 MHz, depicted in section 4 below): δ = 7.04 (s, 1H, C18H), 6.11 (ddd, J_1 = 6.3 Hz, J_2 = 2.6 Hz, , J_3 = 1.9 Hz, 1H, C1H), 6.07 (dd, , J_1 = 6.3 Hz, , J_2 = 1.9 Hz, 1H, C2H), 5.93 (d, J = 5.9 Hz, 1H, C19H), 4.72 (d, J = 0.7 Hz, **$^1J_{C\text{-}H}$ = 160 Hz**, 1H, C6H), 4.68 (ddd, , J_1 = 5.1 Hz, , J_2 = 2.6 Hz, , J_3 = 1.5 Hz, 1H, C5H), 4.43 (t, J = 5.5 Hz, 1H, C20H), 4.23 (dd, , J_1 = 5.4 Hz, J_2 = 3.6 Hz, 1H, C21H), 4.16 (m, 4H, C3H, C4H, C12H), 4.02 (q, , J = 3.4 Hz, 1H, C22H), 3.98 (dd, , J_1 = 3.2 Hz, J_2 = 0.4 Hz, 1H, C7H), 3.87 (dd, , J_1 = 11.8 Hz, J_2 = 2.3 Hz, 1H, C11Ha), 3.81 (dd, , J_1 = 12.2 Hz, J_2 = 3.1 Hz, 1H, C23Ha), 3.75 (dd, J_1 = 12.0 Hz, , J_2 = 5.6 Hz, C11Hb), 3.72 (dd, J_1 = 12.2 Hz, , J_2 = 3.6 Hz, 1H, C23Hb), 3.62 (t, J = 9.6 Hz, 1H, C9H), 3.49 (dd, J_1 = 9.4 Hz, J_2 = 3.2 Hz, 1H, C8H), 3.28 (ddd, J_1 = 9.8 Hz, J_2 = 5.5 Hz, J_3 = 2.3 Hz, C10H), 1.93 (s, 3H, OAc^-) ppm.

^{13}C-NMR (methanol-D_4, 150 MHz, depicted in section 4 below): δ = 162.3 (C12), 154.4 (C14), 153.6 (C15), 136.3 (C1), 132.9 (C2), 130.9 (arom. C), 130.6 (arom C), 119.4 (C18), 113.9 (C17),

102.5 (C6), 100.8 (C16), 89.7 (C19), 86.5 (C22), 84.9 (C4), 78.5 (C10), 75.7 (C20), 75.1 (C8), 75.0 (C5), 72.3 (C7), 72.2 (C21), 68.2 (C9), 65.9 (C3), 63.3 (C23), 62.6 (C11), 43.9 (C12), 22.0 ($CH_3CO_2^-$) ppm.

Note: A ^{1}H-^{13}C- -HMBC spectrum of this compound is shown in section 4 below.

HRMS (ESI^+) m/z calc. for $C_{23}H_{33}N_5O_{12}$, $[M+H]^+$: 572.2199; found: 572.2200.

3.3 Regioselective protection of 12 towards cyclopentene precursors 13-15

(9H-fluoren-9-yl)methyl((1S,4S,5R)-4,5-*para*-methoxybenzylidene-2-en-1-yl)carbamate (29)

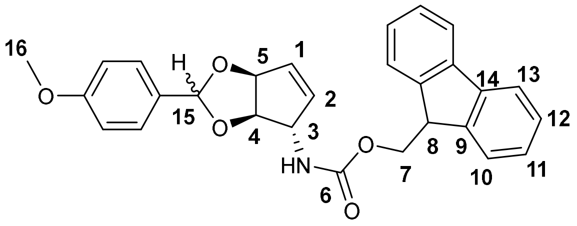

12 (1.50 g, 4.50 mmol, 1.00 eq) was dissolved in DMF (75 mL) and anisaldehyde dimethyl acetal (0.92 mL, 984 mg, 5.40 mmol, 1.20 eq) and racemic camphor-10-sulfonic acid (105 mg, 0.45 mmol, 0.10 eq) were added subsequently. The reaction was stirred at room temperature for 2 h. Saturated aqueous $NaHCO_3$-solution was added and the phases were separated. After extracting the aqueous phase with DCM (3 x 200 mL), the organic phases were dried over Na_2SO_4, filtered and the solvent was evaporated *in vacuo*. Column chromatographic purification (EtOAc/*iso*-hexane 1:5 → EtOAc) gave the product (2.00 g, 4.39 mmol, 98%, 2 Isomers, A:B = 1:0.35) as colorless solid.

R_f (EtOAc/*iso*-hexane 1:3) = 0.30 (A+B, copolar).

^{1}H-NMR (600 MHz, $CDCl_3$, depicted in section 4 below): δ = 7.77 (d, *J* = 7.6 Hz, 2H, C13H), 7.58 (d, *J* = 7.4 Hz, 2H, C10H), 7.39 (m, 4H, C12H, arom. CH), 7.32 (m, 2H, C11H), 6.88 (m, 2H, arom. CH), 6.07 (m, 1 H, C1H (Isomer A + Isomer B)), 5.99 (m, 1H, C2H (Isomer B), 5.81 (m, 1H, C2H (Isomer B)), 5.85 (s, 1H, C15H (Isomer A)), 5.65 (s, 1H, C15H (Isomer B)), 5.49 (m, 1H, C5H), 4.76 (m, 2H, C3H), 4.59 (m, 1H, C4H), 4.46 (d, J = 6.8 Hz, 2H, C7H), 4.22 (m, 1H, C8H), 3.81 (s, 3H, OCH_3 (Isomer B)), 3.80 (s, 3H, OCH_3 (Isomer A) ppm.

^{13}C-NMR (150 MHz, $CDCl_3$, depicted in section 4 below): δ = 160.7 (arom. C), 155.8 (C6), 144.0 (C9), 141.5 (C14), 132.6 (C2 (Isomer A), 135.6 (C1, Isomer A + B), 134.8 (C1, Isomer A), 129.0 (arom. C), 128.4 (arom. C), 127.9 (C12), 127.2 (C11), 125.1 (C10), 120.2 (C13), 113.9 (arom. C), 105.6 (C15, Isomer A), 101.8 (C15, Isomer B), 85.4 (C4 Isomer A), 84.8 (C5), 84.0 (C4 Isomer B), 66.8 (C7), 62.4 (C3), 55.5 (OCH_3), 47.4 (C8) ppm.

HRMS (ESI^+) m/z calc. for $C_{28}H_{25}NO_5Na$, $[M+Na]^+$: 478.1630; found: 478.1629.

(9H-fluoren-9-yl)methyl((1S,4S,5R)-5-hydroxy-4-(*para*-methoxybenzyl)oxycyclopent-2-en-1-yl)carbamate (13)

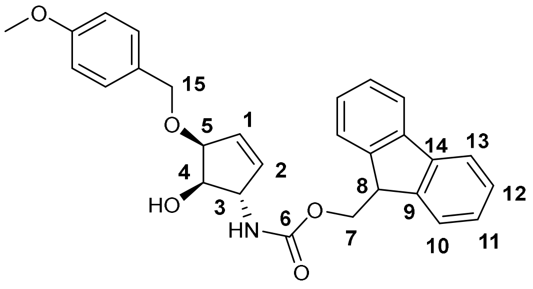

29 (2.00 g, 4.39 mmol, 1.00 eq) was dissolved in DCM (60 mL) and cooled to -78 °C (dry ice/acetone). To ensure temperature equilibration, the mixture was stirred for 30 min. Then DIBAL-H (35.1 mL, 1 M solution in hexane, 35.1 mmol, 8.00 eq) was added slowly over 1.5 h ensuring that the temperature stays constant. After complete addition, the reaction mixture was stirred for another 1.5 h at -78 °C. Next, saturated aqueous Rochelle salt solution (30 mL) was added and the mixture was warmed to room temperature. Then, more DCM (150 mL) and Rochelle salt solution (500 mL) was added and the phases were separated. The aqueous phase was extracted with DCM (3 x 150 mL) and the organic phases were dried over Na_2SO_4, filtered and the solvent was evaporated. Column chromatographic purification (EtOAc/*iso*-hexane 3:1) gave the product (1.71 g, 3.73 mmol, 85%) as colorless solid.

R_f (EtOAc/*iso*-hexane 1:3) = 0.22.

^{1}H-NMR (600 MHz, $CDCl_3$, depicted in section 4 below): δ = 7.76 (d, *J* = 7.7 Hz, 2H, C13H), 7.59 (m, 2H, C10H), 7.40 (t, *J* = 7.4 Hz, 2H, C12H), 7.31 (tt, J_1 = 7.4 Hz, J_2 = 1.1 Hz, 2H, C11H), 7.26 (m, 2H (overlapping with solvent peak), arom. CH), 6.89 (d, *J* = 8.3 Hz, arom. CH), 5.97 (m, 1H, C1H), 5.93 (m, 1H, C2H), 4.81 (d, *J* = 7.7 Hz, 1H, C4OH), 4.61 (m, 1H, C3H), 4.56 (m, 2H, C15H), 4.43 (m, 3H, C5H, C7H), 4.22 (m, 1H, C8H), 3.99 (m, 1H, C4H), 3.81 (s, 3H, OCH_3) ppm.

^{13}C-NMR (150 MHz, $CDCl_3$, depicted in section 4 below): δ = 159.5 (arom. C), 156.2 (C6), 144.0 (C9), 141.5 (C14), 136.0 (C2), 132.0 (C1), 125.2 (C10), 120.1 (C13), 127.8 (C12), 127.2 (C11), 129.6 (arom. C), 129.0 (arom. C), 114.1 (arom. C), 80.02 (C5), 77.3 (C4), 71.8 (C15), 66. 8 (C7), 62.8 (C3), 55.3 (OCH_3), 47.2 (C8) ppm.

HRMS (ESI^+) m/z calc. for $C_{28}H_{27}NO_5Na$, $[M+Na]^+$: 480.1787; found: 480.1785.

(9H-fluoren-9-yl)methyl((1S,4S,5R)-4-(*para*-methoxybenzyl)oxy-5-((2-trimethylsilylethoxy)-methyl)oxy-cyclopent-2-en-1-yl)carbamate (30)

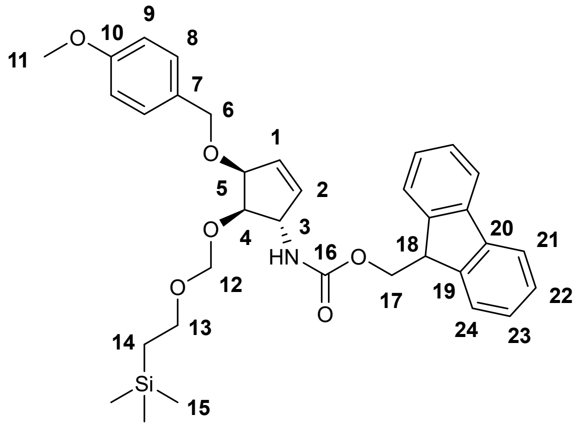

13 (800 mg, 1.75 mmol, 1.00 eq) was dissolved in DMF (16 mL) and SEM-Cl (1.55 mL, 1.46 g, 8.74 mmol, 5.00 eq), pyridine (2.11 mL, 26.2 mmol, 15.0 eq) and NBu_4I (64.6 mg, 0.175 mmol, 0.10 eq) were added subsequently. The reaction was stirred at 70 °C for 18 h before the solvent was evaporated *in vacuo*. Column chromatographic purification (EtOAc/*iso*-hexane 5:1 → 4:1) gave the product (767 mg, 1.30 mmol, 75%) as colorless solid.

R_f (EtOAc/*iso*-hexane 1:4) = 0.15.

^{1}H-NMR (600 MHz, $CDCl_3$, depicted in section 4 below): δ = 7.76 (d, *J* = 7.5 Hz, 1H, C21H), 7.59 (t, *J* = 6.0 Hz, 1H, C24H), 7.40 (t, *J* = 7.5 Hz, 1H, C22H), 7.31 (tt, J_1 = 7.5 Hz, J_2 = 1.3 Hz, 2H, C23H), 7.26 (m, 2H, arom. CH (PMB)), 6.87 (d, *J* = 8.4 Hz, 2H, arom. CH (PMB)), 6.01 (s, 1H, C2H), 5.95 (s, 1H, C1H), 4.88 (d, *J* = 4.8 Hz, 1H, C12Ha), 4.78 (m, 2H, C12Hb, C3H), 4.60 (d, *J* = 11.6 Hz, 1H, C6Ha), 4.51 (d, *J* = 11.6 Hz, 1H, C6Hb), 4.45 (m, 2H, C5H, C17Ha), 4.36 (m, 1H, C17Hb), 4.21 (m, 1H, C18H), 3,98 (m, 1H, C4H), 3.80 (s, 3H, C11H), 3.71 (m, 2H, C13H), 0.94 (t, *J* = 8.6 Hz, 2H, C14H), -0.02 (s, 9H, $-Si(CH_3)_3$) ppm.

^{13}C-NMR (125 MHz, $CDCl_3$, depicted in section 4 below): δ = 159.2 (C10), 155.8 (C16), 143.9 (C19), 141.3 (C20), 135.9 (C2), 131.7 (C1), 130.5 (C7), 129.3 (C8), 127.7 (C22), 127.0 (C23), 125.0 (C24), 120.0 (C21), 113.7 (C9), 94.3 (C12), 81.3 (C4), 78.6 (C5), 70.7 (C6), 66.7 (C17), 65.7 (C13), 60.6 (C3), 55.2 (C11), 47.2 (C18), 18.1 (C14), -1.50 (C15) ppm.

HRMS (ESI^+) m/z calc. for $C_{34}H_{41}NO_6SiNa$, $[M+Na]^+$: 610.2601; found: 610.2599.

(9H-fluoren-9-yl)methyl((1S,4S,5R)-4-hydroxy-5-((2-trimethylsilylethoxy)-methyl)oxy-cyclopent-2-en-1-yl)carbamate (14)

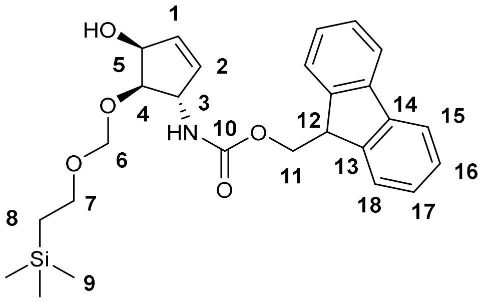

30 (506 mg, 0.86 mmol) was dissolved in DCM (22.5 mL) and cooled to 0 °C. The mixture was stirred at 0 °C for 30 min to ensure temperature equilibration. Next, TFA (2.5 mL) was added and the reaction was stirred at 0 °C for 5 min. The reaction was stopped by first addition of $HNEt_3OAc$ (1 M in DCM, 20 mL) and then saturated aqueous $NaHCO_3$-solution in quick succession. The phases were separated and the aqueous phase was extracted with DCM (3 x 50 mL). The organic phases were dried over $MgSO_4$, filtered and the solvent was evaporated *in vacuo*. Column chromatographic purification (EtOAc/*iso*-hexane 2:1) gave the product (335 mg, 0.71 mmol, 83%) as colorless solid.

R_f (EtOAc/*iso*-hexane 1:2) = 0.20

^{1}H-NMR (600 MHz, $CDCl_3$, depicted in section 4 below): δ = 7.77 (dt, J_1 = 7.5 Hz, J_2 = 0.9 Hz, 2H, C15H), 7.59 (m, 2H, C18H), 7.40 (m, 2H, C16H), 7.32 (td, J_1 = 7.4 Hz, J_2 = 1.1 Hz, 2H, C17H), 6.03 (m, 1H, C1H), 5.92 (m, 1H, C2H), 4.85 (m, 2H, C5H, C6Ha), 4.71 (m, 2H, C3H, C6Hb), 4.43 (m, 2H, C11H), 4.22 (m, 1H, C12H), 3.95 (m, 1H, C4H), 3.68 (m, 2H, C7H), 0.96 (t, J = 8.6 Hz, 2H, C8H), 0.01 (s, 9H, $(SiCH_3)_3$) ppm.

^{13}C-NMR (125 MHz, $CDCl_3$, depicted in section 4 below): δ =144.2 (C13), 141.6 (C14), 135.2 (C2), 134.2 (C1), 128.0 (C16), 127.4 (C17), 125.3 (C18), 120.3 (C15), 95.1 (C6), 82.2 (C4), 73.6 (C5), 67.0 (C11), 66.4 (C7), 60.9 (C3), 47.5 (C12), 18.4 (C8), -1.15 $(SiCH_3)_3$) ppm.

HRMS (ESI$^+$) m/z calc. for $C_{26}H_{33}NO_5SiNa$, $[M+Na]^+$: 490.2026; found: 490.2024.

(9H-fluoren-9-yl)methyl((1S,4S,5R)-5-((tert-butyldimethylsilyl)oxy)-4-hydroxycyclopent-2-en-1-yl)carbamate (15)

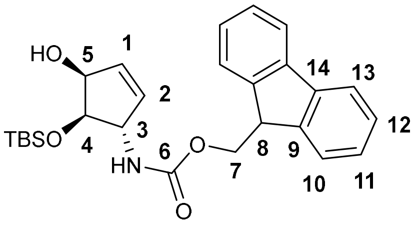

12 (200 mg, 0.59 mmol, 1.00 eq) was dissolved in DMF (6 mL) and cooled to -10 °C (NaCl/ice bath). Next, TBSOTf (163 µL, 188 mg, 0.71 mmol, 1.20 eq) was added and the reaction was stirred for 2 h at -10 °C. Saturated aqueous $NaHCO_3$-solution was added and the phases were separated. After extracting the aqueous phase with DCM (3 x 50 mL), the organic phases were dried over Na_2SO_4, filtered and the solvent was evaporated *in vacuo*. Column chromatographic purification (EtOAc/*iso*-hexane 1:5) gave the product (110 mg, 0.24 mmol, 41%) as colorless solid.

R_f (EtOAc/*iso*-hexane 1:3) = 0.12.

^{1}H-NMR (600 MHz, $CDCl_3$, depicted in section 4 below): δ = 7.77 (d, *J* = 7.5 Hz, 2H, C13H), 7.58 (d, *J* = 7.5 Hz, 2H, C10H), 7.40 (t, *J* = 7.5 Hz, 2H, C12H), 7.31 (t, *J* = 7.5 Hz, 2H, C11H), 6.00 (d, *J* = 5.7 Hz, 1H, C1H), 5.83 (d, *J* = 6.0 Hz, 1H, C2H), 4.56 (m, 2H, C3H, C5H), 4.43 (m, 2H, C6H), 4.21 (t, *J* = 6.8 Hz, 1H, C7H), 4.04 (m, 1H, C4H), 0.92 (s, 9H, $SiC(CH_3)_3$), 0.17 (s, 3H, $SiCH_3$), 0.14 (s, 3H, $SiCH_3$) ppm.

^{13}C-NMR (150 MHz, $CDCl_3$, depicted in section 4 below): δ = 155.8 (C6), 144.0 (C9), 141.5 (C14), 135.8 (C1), 133.5 (C2), 127.8 (C12), 127.2 (C11), 125.1 (C10), 120.1 (C13), 77.5 (C4), 74.2(C5), 66.7 (C7), 62.6 (C3), 47.4 (C8), 25.9 ($SiC(CH_3)_3$)), 18.2 ($SiC(CH_3)_3$)), -4.5 ($SiCH_3$), -5.0 ($SiCH_3$) ppm.

^{29}Si-NMR (80 MHz, $CDCl_3$, depicted in section 4 below): δ = 23.2 ppm.

HRMS (ESI^+) m/z calc. for $C_{26}H_{37}N_2O_4Si$, $[M+NH_4]^+$: 469.2517; found: 469.2520.

3.4 Synthesis of glycosyl donor 16

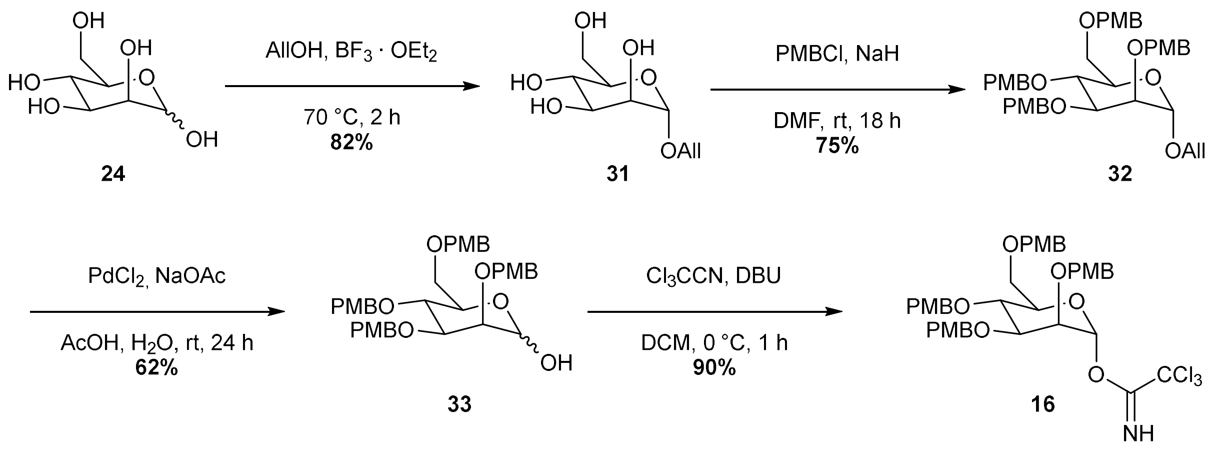

Supplementary Figure 9: Synthesis of glycosyl donor **16** in 4 steps starting from D-mannose (overall yield: 34 %)

1-*O*-allyl-D-mannopyranose (31)[6]

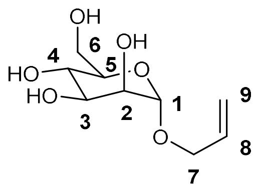

D-Mannose (5.00 g, 27.8 mmol, 1.00 eq) was suspended in AllOH (25 mL) and $BF_3 \cdot OEt_2$ (343 µL, 2.78 mmol, 0.10 eq) was added. The reaction was heated to 70 °C for 2 h. After cooling to room temperature, the mixture was neutralized with NEt_3 and the solvent was evaporated *in vacuo*. Column chromatographic purification (MeOH/DCM 1:10) gave the product (5.02 g, 22.8 mmol, 82%) as colorless gum.

R_f (MeOH/DCM 1:5) = 0.46.

^{1}H-NMR (600 MHz, methanol-D_4): δ = 5.98 (m, 1H, C8H), 5.34 (dq, J_1 = 17.2 Hz, J_2 = 1.7 Hz, 1H, C9Ha), 5.21 (m, 1H, C9Hb), 4.83 (d, J = 1.7 Hz, 1H, C1H), 4.26 (ddt, J_1 = 13.1 Hz, J_2 = 5.1 Hz, J_3 = 1.5 Hz, 1H, C7Ha), 4.05 (ddt, J_1 = 13.1 Hz, J_2 = 6.0 Hz, J_3 = 1.4 Hz, 1H, C7Hb), 3.87 (dd, J_1 = 11.8 Hz, J_2 = 2.4 Hz, 1H, C6Ha), 3.85 (dd, J_1 = 3.4 Hz, J_2 = 1.7 Hz, 1H, C2H), 3.75 (m, 2H, C3H, C6Hb), 3.65 (t, J = 9.5 Hz, 1H, C4H), 3.58 (m, 1H, C5H) ppm.

^{13}C-NMR (125 MHz, methanol-D_4): δ = 135.5 (C8), 117.2 (C9), 100.8 (C1), 74.8 (C5), 72.7 (C3), 72.6 (C2), 68.8 (C7), 68.7 (C4), 63.4 (C6) ppm.

HRMS (ESI^+) m/z calc. for $C_9H_{16}O_6Na$, $[M+Na]^+$: 243.0845; found: 243.0838.

1-*O*-allyl-2,3,4,6-*O*-(*para*-methoxybenzyl)-D-mannopyranose (32)

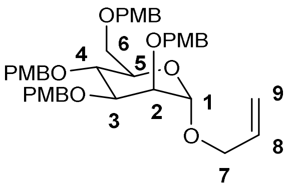

31 (1.84 g, 8.36 mmol, 1.00 eq) was dissolved in DMF (19 mL). NaH (2.00 g, 50.1 mmol, 6.00 eq) was added at 0 °C and the reaction was stirred for 30 min before PMB-Cl (6.80 mL, 7.85 g, 50.1 mmol, 6.00 eq) was added dropwise. The mixture was stirred at room temperature for 18 h and the poured into saturated aqueous $NaHCO_3$-solution. The solution was extracted with DCM (3 x 200 mL), the combined organic phases were dried over Na_2SO_4, filtered, and the solvent was evaporated *in vacuo*. Column chromatographic purification (EtOAc/*iso*-hexane 1:4 → 1:3) yielded the product (4.39 g, 6.27 mmol, 75%) as colorless syrup.

R_f (EtOAc/*iso*-hexane 1:3) = 0.15.

^{1}H-NMR (400 MHz, $CDCl_3$): δ = 7.28 (m, 6H, arom. CH), 7.04 (m, 2H, arom. CH), 6.83 (m, 8H, arom. CH), 5.83 (m, 1H, C8H), 5.20 (dd, J_1 = 10.4 Hz, J_2 = 1.4 Hz, 1H, C9Ha), 5.13 (dd, J_1 = 10.4 Hz, J_2 = 1.4 Hz, 1H, C9Hb), 4.87 (d, J = 1.8 Hz, 1H, C1H), 4.77 (d, J = 10.3 Hz, 1H, CH_2), 4.63 (m, 3H, CH_2), 4.51 (m, 3H, CH_2), 4.38 (d, J = 10.2 Hz, 1H, CH_2), 4.15 (m, 1H, C7Ha), 3.90 (m, 3H, C4H, C5H, C7Hb), 3.81(s, 3H, OCH_3), 3.80(s, 3H, OCH_3), 3.79 (s, 3H, OCH_3), 3.79 (s, 3H, OCH_3), 3.75 (dd, J_1 = 2.7 Hz, J_2 = 1.9 Hz, 1H, C2H), 3.69 (m, 3H, C3H, C6H) ppm.

^{13}C-NMR (125 MHz, $CDCl_3$): δ = 159.3 (2C, arom. C), 159.1 (2C, arom. C), 134.0 (C8), 130.9 (arom. C), 130.8 (arom. C), 130.6 (arom. C), 130.6 (arom. C), 129.8 (arom. C), 129.6 (2C, arom. C), 129.3 (arom. C), 117.2 (C9), 113.8 (arom. C), 113.8 (arom. C), 113.8 (2C, arom. C), 97.2 (C1), 80.1 (C5), 74.9 (CH_2), 74.8 (2C, C4, CH_2), 74.2 (C2), 72.2 (CH_2), 72.0 (CH_2), 71.9 (C3), 68.9 (C6), 67.8 (C7), 55.4 (OCH_3), 55.4 (OCH_3), 55.4 (OCH_3), 55.4 (OCH_3) ppm.

HRMS (ESI^+) m/z calc. for $C_{51}H_{42}NO_{10}$, $[M+NH_4]^+$: 718.3586; found: 718.3584.

2,3,4,6-*O*-(*para*-methoxybenzyl)-D-mannopyranose (33)[7]

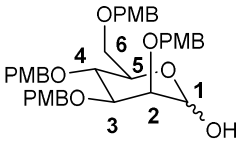

32 (2.70 g, 3.85 mmol, 1.00 eq) was dissolved in AcOH (20 mL) and H_2O (0.65 mL). NaOAc (886 mg, 10.8 mmol, 2.80 eq) and $PdCl_2$ (887 mg, 5.00 mmol, 1.30 eq) were added and the reaction was stirred at room temperature for 24 h. The resulting mixture was diluted with DCM and filtered over celite. The filtrate was washed with saturated aqueous $NaHCO_3$-solution (50 mL) and brine (50 mL), dried over $MgSO_4$, filtered and the solvent was evaporated *in vacuo*. Column chromatographic purification (EtOAc/*iso*-hexane 1:1.5) gave the product (1.57 g, 2.38 mmol, 62%, α:β = 1:0.26) as colorless syrup.

R_f (EtOAc/*iso*-hexane 1:1) = 0.36.

^{1}H-NMR (600 MHz, $CDCl_3$): δ = 7.26 (m, 6H, arom. CH), 7.05 (d, J = 8.3 Hz, 2H, arom. CH), 6.84 (m, 8H, arom. CH), 5.20 (m, 1H, C1Hα), 5.00 (d, 2H, J = 11.5 Hz, CH_2α), 4.88 (s, 1H, C1Hβ), 4.58 (m, 8H, CH_2), 3.97 (ddd, J_1 = 9.8 Hz, J_2 = 6.5 Hz, J_3 = 2.0 Hz, 1H, C5Hα), 3.90 (dd, J_1 = 9.3 Hz, J_2 = 3.0 Hz, 1H, C3Hα), 3.86 (m, 1H, C4Hα), 3.80 (s, 3H, OCH_3), 3.79 (m, 7H, C4Hα, 2 x OCH_3), 3.78 (s, 3H, OCH_3), 3.75 (t, J = 2.5 Hz, 1H, C2Hα), 3.66 (m, C6Hαa, CH_2), 3.61 (dd, J_1 = 10.4 Hz, J_2 = 6.5 Hz, 1H, C6Hαb) ppm.

^{13}C-NMR (600 MHz, $CDCl_3$): δ = 159.3, 159.3, 159.3, 159.3, 159.3, 113.9, 113.9, 113.9, 113.9, 113.8, 130.9, 130.8, 130.6, 130.4, 130.4, 130.3, 130.0, 129.8, 129.8, 129.8, 129.7, 129.6, 129.4, 129.4, 129.4 (arom. C), 97.9 (C1β), 93.0 (C1α), 79.5 (C3α), 75.8, 75.4, 75.0 (C4α), 74.8 (CH_2), 74.8, 74.4, 74.4 (C2α), 73.1, 73.1 (CH_2), 72.5, 72.4, 72.4 (CH_2), 72.0 (CH_2), 71.9, 71.9 (C5α), 71.6, 69.3 (C6α), 68.7, 55.4, 55.4, 55.4 (OCH_3) ppm.

NOTE: Signals for β-Anomer cannot be assigned unambiguously due to the low abundance in the mixture and overlapping signals.

HRMS (ESI^+) m/z calc. for $C_{38}H_{44}O_{10}Na$, $[M+Na]^+$: 683.3832; found: 683.2831.

Analytical data in accordance with literature.

2,3,4,6-*O*-(*para*-methoxybenzyl)-α-D-mannopyranosyltrichloroacetimidate (16)

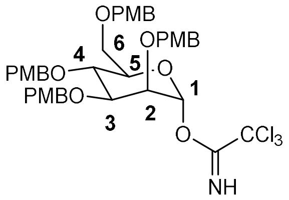

33 (720 mg, 1.09 mmol, 1.00 eq) was dissolved in DCM (12 mL). Cl_3CCN (1.09 mL,1.57 g, 10.9 mmol, 10.0 eq) and DBU (33.0 µL, 33.5 mg, 0.22 mmol, 0.20 eq) were added at 0 °C and the reaction was stirred at the same temperature for 1 h. The solvent was evaporated *in vacuo*. Column chromatographic purification (EtOAc/*iso*-hexane 1:3) gave the product (789 mg, 0.98 mmol, 90%) as colorless syrup. (NOTE: a slight yellow color indicates amine impurities, which hamper glycosylation)

$\mathbf{R_f}$ (EtOAc/*iso*-hexane 1:3) = 0.32.

^{1}H-NMR (600 MHz, $CDCl_3$): δ = 8.51 (s, 1H, NH), 7.33 (d, J = 8.6 Hz, 2H, arom. CH), 7.25 (m with solvent peak, 2H, arom. CH), 7.22 (d, J = 8.5 Hz, 2H, arom. CH), 7.09 (d, J = 8.5 Hz, 2H, arom. CH), 6.83 (m, 8H, arom. CH), 6.33 (d, J = 2.0 Hz, 1H, C1H), 4.79 (d, J = 10.2 Hz, 1H, CH_2), 4.69 (m, 2H, CH_2), 4.61 (d, J = 11.7 Hz, 1H, CH_2), 4.52 (m, 2H, CH_2), 4.44 (m, 2H, CH_2), 4.06 (t, J = 9.7 Hz, 1H, C4H), 3.92 (ddd, J_1 = 9.8 Hz, J_2 = 4.5 Hz, J_3 = 1.7 Hz, 1H, C5H), 3.87 (dd, J_1 = 9.5 Hz, J_2 = 3.2 Hz, 1H, C3H), 3.81 (m, 1H, C2H), 3.80 (s, 3H, OCH_3), 3.79 (s, 3H, OCH_3), 3.79 (s, 3H, OCH_3), 3.78 (s, 3H, OCH_3), 3.76 (m, 1H, C6Ha), 3.67 (dd, J_1 = 11.2 Hz, J_2 = 1.9 Hz, 1H, C6Hb) ppm.

^{13}C-NMR (600 MHz, $CDCl_3$): δ = 160.6 (OCNH), 159.4, 159.4, 159.4, 159.3, 130.6, 130.6, 130.4, 130.2, 130.0 129.8, 129.7, 129.6, 113.9, 113.9, 113.9, 133.8 (all aromatic C), 96.3 (C1), 91.3 (CCl_3), 75.1 (CH_2), 75.0 (C5), 74.0 (C4), 73.1 (CH_2), 73.1 (C2), 78.6 (C3), 72.3 (CH_2), 72.0 (CH_2), 68.5 (C6), 55.4, 55.4, 55.4, 55.4 (all OCH_3) ppm.

HRMS (ESI^+) m/z calc. for $C_{40}H_{44}NO_{10}Cl_3Na$, $[M+Na]^+$: 826.1928; found: 826.1922.

3.5 Synthesis of β-*allyl*-manQ (10), α-*homoallyl*-manQ (11), and of α-*allyl*-manQ (4)

(9H-fluoren-9-yl)methyl((1S,4S,5R)-4-(((2R,4aR,6R,7S,8S,8aR)-7,8-bis((4-methoxybenzyl)oxy)-2-phenylhexahydropyrano[3,2-d][1,3]dioxin-6-yl)oxy)-5-((tert-butyldimethylsilyl)oxy)cyclopent-2-en-1-yl)carbamate (17)

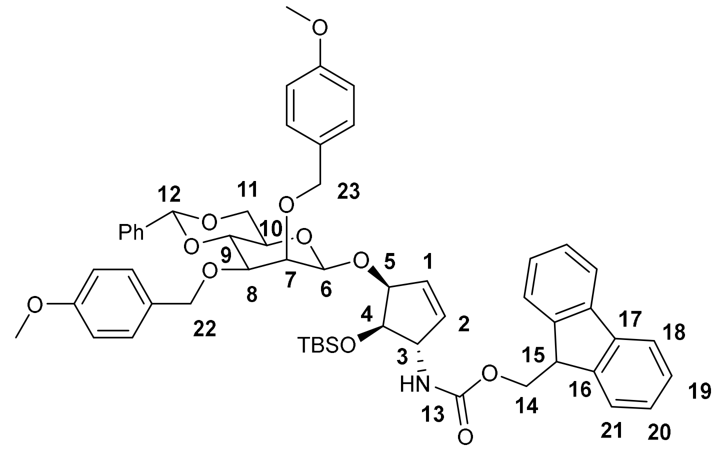

AgOTf (68.3 mg, 266 µmol, 3.00 eq) was dissolved in DCM (1 mL) and pulverized 3 Å molsieves (20 w% of the solvent volume) were added. The mixture was cooled to -78 °C before a solution of PhSCl (32.1 mg, 222 µmol, 2.50 eq) in DCM (1 mL) was added. The reaction was stirred for 5 min at – 78 °C. Next, a solution of sugar donor **5** (53.2 mg, 88.6 µmol, 1.00 eq) and 2,6-di-*tert*-butyl-4-methylpyridine (54.6 mg, 266 µmol, 3.00 eq) in DCM (1 mL) was added dropwise and the reaction was stirred for 15 min at -78 °C. Then, a solution of glycosyl acceptor **15** (40.0 mg, 88.6 µmol, 1.00 eq) in DCM (1 mL) was added and the reaction was kept stirring at – 78 °C for 2 h. A saturated aqueous $NaHCO_3$-solution (5 mL) and DCM (10 mL) were added and the reaction was warmed to room temperature before it was filtered over celite. The phases were separated and the organic phase was washed with brine and dried over Na_2SO_4. After filtration, the solvent was removed *in vacuo* and the residue was purified by column chromatography (EtOAc/*iso*-hexane 1:10 → 1:5 → 1:3) to yield the product (43.4 mg, 46.1 µmol, 52%) as colorless solid.

$\mathbf{R_f}$ (EtOAc/*iso*-hexane 1:3) = 0.12.

^{1}H-NMR (600 MHz, $CDCl_3$, depicted in section 4 below): δ = 7.78 (d, *J* = 7.6 Hz, 2H, C18H), 7.60 (t, *J* = 6.9 Hz, 2H, C21H), 7.51 (d, *J* = 7.0 Hz, 2H, arom. CH), 7.38 (m, xH, C19H, arom. CH), 7.32 (t, *J* = 7.4 Hz, 2H, C20H), 7.15 (d, *J* = 7.2 Hz, 2H, arom. CH), 6.88 (d, *J* = 8.2 Hz, 2H, arom. CH), 6.81 (d, *J* = 8.3 Hz, 2H, arom. CH), 6.00 (m, 1H, C1H), 5.88 (d, *J* = 6.2 Hz, 1H, C2H), 5.61 (s, 1H, C12H), 4.88 (d, *J* = 12.0 Hz, 1H, C23Ha), 4.79 (d, *J* = 12.0 Hz, 1H, C23Hb), 4.71 (m, 3H, C3H, C6H), 4.46 (m, 5H, C5H, C14H, C21H), 4.31 (dd, J_1 = 10.4 Hz J_2 = 4.8 Hz, C11Ha), 4.22 (t, *J* = 6.6 Hz, 1H, C15H), 4.17 (t, *J* = 9.6 Hz, C9H), 3.96 (m, 3H, C4H, C7H, C11Hb), 3.81 (s, 3H, OCH_3), 3.79 (s, 3H, OCH_3), 3.48 (dd, J_1 = 9.9 Hz, J_2 = 3.2 Hz, 1H, C8H), 3.27 (td, J_1 = 9.7 Hz, J_2 = 4.8 Hz, 1H, C10H), 0.81 (s, 9H, $SiC(CH_3)_3$), 0.04 (s, 3H, $SiCH_3$), 0.00 (s, 3H, $SiCH_3$) ppm.

^{13}C-NMR (150 MHz, $CDCl_3$, depicted in section 4 below): δ = 159.2 (2C, arom. C), 155.9 (C13), 144.0 (C15), 141.5 (C16), 137.0 (C1), 131.3 (C2), 130.7 (arom. C), 130.6 (arom. C), 130.5 (arom. C), 130.4 (arom. C), 129.3 (arom. C), 128.9 (arom. C), 128.3 (arom. C), 127.8 (C19), 127.2 (C20), 126.2 (arom C), 125.1 (C21), 120.1 (C18), 113.8 (arom. C), 113.7 (arom. C), 102.1 (C6), 101.4 (C12), 80.1 (C5), 78.7 (C7), 78.5 (C9), 77.5 (C8), 74.6 (C4), 74.4 (C23), 71.9 (C22), 67.8 (C10), 68.7 (C11), 66.8 (C14), 61.0 (C3), 55.4 (OCH_3), 55.3 (OCH_3), 47.4 (C15), 25.8 ($SiC(CH_3)_3$), 18.1 ($SiC(CH_3)_3$), -4.5 ($SiCH_3$), -4.7 ($SiCH_3$) ppm.

HRMS (ESI$^+$) m/z calc. for $C_{55}H_{67}N_2O_{11}Si$, $[M+NH_4]^+$: 959.4509; found: 959.4522.

(9H-fluoren-9-yl)methyl((1S,4S,5R)-4-((4-methoxybenzyl)oxy)-5-(((2R,3R,4R,5S,6S)-3,4,5-tris((4-methoxybenzyl)oxy)-6-(((4-methoxybenzyl)oxy)methyl)tetrahydro-2H-pyran-2-yl)oxy)cyclopent-2-en-1-yl)carbamate (18)

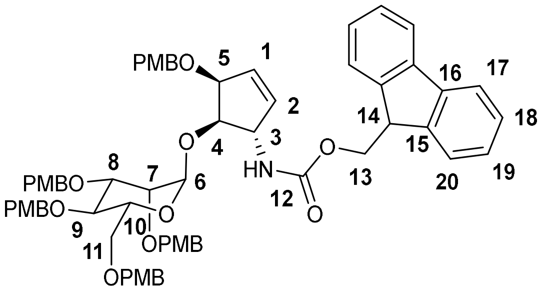

13 (20.0 mg, 43.7 µmol, 1.00 eq) and **16** (42.2 mg, 62.5 µmol, 1.20 eq) were dissolved in THF (3 mL) and activated 3 Å molsieves (20 w%) were added. The solution was stirred at 0 °C for 30 min. TMSOTf (0.1 M in THF, 87.4 µL, 8.74 µmol, 0.20 eq, pre-dried over 3 Å molsieves for 30 min) was added dropwise and the reaction was stirred at 0 °C for 1 h. Saturated aqueous $NaHCO_3$-solution (3 mL) was added and the mixture was warmed to room temperature, diluted with DCM (20 mL) and H_2O (20 mL), filtered and the phases were separated. The aqueous phase was re-extracted with DCM (3 x 20 mL), the combined organic phases were dried over Na_2SO_4, filtered and the solvent was evaporated *in vacuo*. Column Chromatographic purification (EtOAc/Hexane 1:4 → 1:2) gave the product (26.4 mg, 24.0 µmol, 55%) as colorless solid.

R_f (EtOAc/*iso*-hexane 1:3) = 0.12.

^{1}H-NMR (600 MHz, $CDCl_3$, depicted in section 4 below): δ = 7.73 (d, *J* = 7.8 Hz, 2H, C17H), 7.57 (d, *J* = 7.6 Hz, 2H, C20H), 7.35 (t, *J* = 7.4 Hz, 2H, C18H), 7.22 (m, 10H, C19H, arom. CH), 7.04 (m, 2H, arom. CH),6.80 (m, 10H, arom. CH), 6.01 (m, 1H, C1H), 5.90 (m, 1H, C2H), 4.96 (m, 1H), 4.94 (m, **$^1J_{C-H}$ = 170.3 Hz**, 1H, C6H), 4.77 (t, J = 9.9 Hz, 1H), 4.61 (d, J = 3.7 Hz, 1H), 4.47 (m,), 4.16 (m), 3.92 (m), 3.77 (m, OCH_3) ppm.

NOTE: Signals cannot be assigned unambiguously. ^{13}C-NMR-data are not assigned, the ^{13}C-spectrum is depicted in section 4 below.

HRMS (ESI$^+$) m/z calc. for $C_{66}H_{77}N_3O_{14}$, $[M+NH_4]^+$: 1117.5056; found: 1117.5084.

(9H-fluoren-9-yl)methyl((1S,4S,5R)-5-((2-(trimethylsilyl)ethoxy)methoxy)-4-(((2R,3R,4R,5S,6S)-3,4,5-tris((4-methoxybenzyl)oxy)-6-(((4-methoxybenzyl)oxy)methyl)-tetrahydro-2H-pyran-2-yl)oxy)cyclopent-2-en-1-yl)carbamate (19)

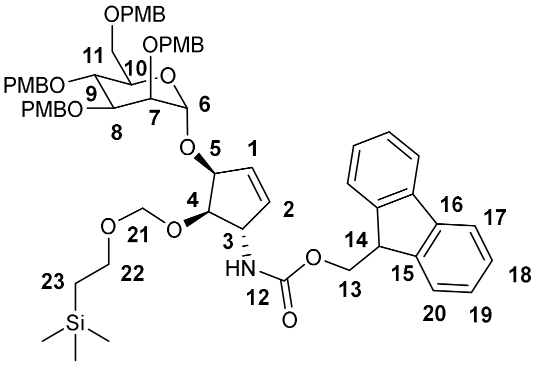

14 and **16** were dissolved in THF (2 mL) and 3 Å molsieves (20 w% of solvent) were added. The mixture was stirred at 0 °C for 20 min before a solution of TMSOTf (231 µL, 0.1 M in THF) was added. The reaction was stirred at 0 °C for 1.5 h before saturated aqueous $NaHCO_3$-solution (5 mL) was added. DCM (20 mL) was added and the phases were separated. The aqueous phase was re-extracted with DCM (3 x 20 mL). The combined organic phases were dried over Na_2SO_4, filtered and the solvent was evaporated *in vacuo*. Column chromatographic purification (EtOAc/*iso*-hexane 1:4 → 1:3) gave an anomeric mixture of the product as colorless solid.

R_f (EtOAc/*iso*-hexane 1:3) = 0.14.

^{1}H-NMR (600 MHz, $CDCl_3$, depicted in section 4 below): δ = 7.79 (dd, J_1 = 7.6 Hz J_2 = 3.8 Hz, 2H, C17H), 7.61 (m, 2H, C20H), 7.42 (m, 2H, C18H), 7.33 (t, J = 7.6 Hz, 1H, C19H), 7.28 (m with solvent peak, arom. CH), 7.07 (m, 2H, arom. CH), 6.85 (m, arom. CH), 6.03 (m, 1H, C1H), 5.99 (m, 1H, C2H), 5.01 (m, 1H), 4.87 (m, 1H, C6H), 4.62 (m, 18H), 4.25 (m, 1H), 3.79 (m, OCH_3), 3.54 (m, 1H), 3.43 (m, 1H), 0.95 (t, J = 7.3 Hz, 1H, C23H), -0.01 (s, 3H, $SiCH_3$), -0.02 (s, 6H, $SiCH_3$) ppm.

NOTE: Due to the inseparable anomeric mixture received, ^{1}H-NMR data refer to the most important features proving the formation of the glycosylation adduct from the starting materials. The anomeric mixture was separated *via* HPLC after completing the synthesis. ^{13}C-NMR-data are not assigned, but the ^{13}C-spectrum is depicted in section 4 below.

HRMS (ESI$^+$) m/z calc. for $C_{64}H_{76}NO_{14}Si$, $[M+H]^+$: 1110.5030; found: 1110.5033.

β-*allyl*-mannosyl-queuosine (10)

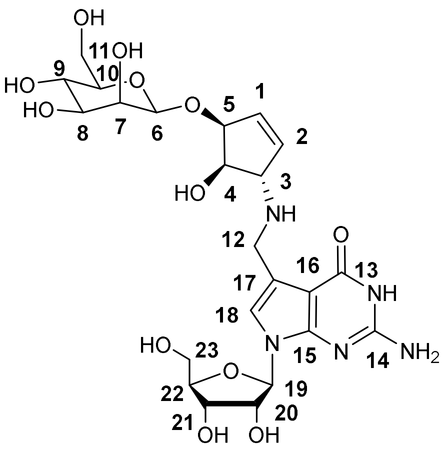

17 (40 mg, 42.4 µmol) was dissolved in MeCN (1.5 mL) and DBU (224 µL) was added. The reaction was stirred for 1 h at room temperature and then neutralized with AcOH. The solvent was evaporated in vacuo and the crude residue purified by column chromatography (MeOH/DCM 1:20). The resulting product (21 mg) was identified by LC-MS and subsequently used for the next step. For that, it was dissolved in MeOH (0.25 mL) and **8** (21 mg, 1.00 eq) was added. AcOH (1.00 µL) was added and the reaction was stirred for 2 h at room temperature. Next the reaction was cooled to 0 °C and $NaBH_4$ (2.87 mg, 75.9 µmol, 2.60 eq) was added. The reaction was stirred at 0 °C for 15 min before the solvent was evaporated. Product formation was confirmed by LC-MS. The residue was dissolved in DCM (0.9 mL) and TFA (0.1 mL) was added at 0 °C. The reaction was stirred at 0 °C for 20 min before it was neutralized with NEt_3. After evaporation of the solvent, the product was detected by LC-MS and then re-dissolved in EtOAc (0.5 mL). HF · pyridine (15 µL) was added and the solution was stirred for 1 d before the reaction was stopped by addition of TMSOMe (46 µL). The solvent was evaporated, the crude product was identified by LC-MS and dissolved in MeOH (1 mL). NaOMe (27.0 mg, 0.5 mmol) was added and the reaction was stirred for 2 d until LC-MS indicated completion. The mixture was neutralized with NEt_3 and the solvent was evaporated *in vacuo*. The residue was dissolved in water, filtered and purified by reversed phase HPLC (0-10 % buffer B, 45 min) to afford the product **10** (1.30 mg, 2.33 µmol, 5% over 6 steps) as colorless solid.

RP-HPLC: R_t (C18-column, 0-10% buffer B, 45 min) = 21.7 min.

^{1}H-NMR (800 MHz, D_2O, depicted in section 4 below): δ = 7.13 (s, 1H, C18H), 6.25 (m, 1H, C1H), 6.10 (dd, J_1 = 6.3 Hz, J_2 = 1.7 Hz, 1H, C2H), 5.97 (d, J = 6.5 Hz, 1H, C19H), 4.70 (m, covered by solvent peak, 2H, C5H, C6H (**$^1J_{C\text{-}H}$ = 160.2 Hz**)), 4.58 (dd, J_1 = 6.5 Hz, J_2 = 5.4 Hz, 1H, C20H), 4.41 (m, 3H, C4H, C12H), 4.32 (dd, J_1 = 5.4 Hz, J_2 = 3.3 Hz, 1H, C21H), 4.27 (m, 1H, C3H), 4.16 (q, J = 3.6 Hz, 1H, C22H), 4.04 (d, J = 3.1 Hz, 1H, C7H), 3.88 (dd, J_1 = 12.3 Hz, J_2 = 2.3 Hz, 1H, C11Ha), 3.82 (dd, J_1 = 12.6 Hz, J_2 = 3.3 Hz, 1H, C23Ha), 3.77 (dd, J_1 = 12.6 Hz, J_2 = 4.3 Hz, 1H, C23Hb), 3.70 (dd, J_1 = 12.3 Hz, J_2 = 6.6 Hz, 1H, C11Hb), 3.62 (dd, J_1 = 9.6 Hz, J_2 = 3.3 Hz, 1H, C8H), 3.54 (t, J = 9.7 Hz, 1H, C9H), 3.36 (ddd, J_1 = 9.3 Hz, J_2 = 6.5 Hz, J_3 = 2.3 Hz, 1H, C10H) ppm.

^{13}C-NMR (HSQC, HMBC, D_2O): δ = 151.7 (C14/C15), 152.7 (C14/C15), 136.1 (C1), 130.0 (C2), 118.9 (C18), 100.2 (C16/17), 98.8 (C16/17), 99.8 (C6), 86.6 (C19), 84.8 (C22), 80.7 (C5), 76.3 (C10), 73.7 (C4), 73.4 (C20), 72.8 (C11), 70.4 (C21), 70.3 (C7), 66.7 (C9), 66.4 (C3), 61.4 (C23), 60.9 (C11), 42.0 (C12) ppm.

Note: ^{1}H-^{13}C-HSQC and -HMBC spectra of this compound are shown in section 4 below.

HRMS (ESI^{+}) m/z calc. for $C_{23}H_{34}N_5O_{12}$, $[M+H]^{+}$: 572.2199; found: 572.2197.

α-*homoallyl*-mannosyl-queuosine (11)

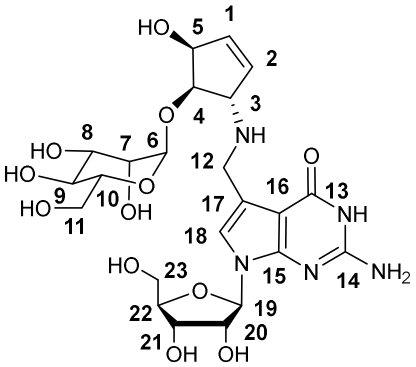

18 (19.0 mg, 17.2 µmol) was dissolved in MeCN (1 mL) and DBU (149 µL, 1.00 mmol) was added. The reaction was stirred at room temperature for 1 h and then neutralized with AcOH. The solvent was evaporated in vacuo to give the free amine, which was detected by LC-MS. The product was dissolved in MeOH and **8** (12.0 mg, 13.7 µmol) was added together with AcOH (1.00 µL). The reaction was stirred for 5 h before it was cooled to 0 °C and $NaBH_4$ (1.34 mg, 35.6 µmol) was added. The reaction was stirred at 0 °c for 20 min, then H_2O (0.5 mL) was added and the reaction was stirred for another 5 min before the solvent was evaporated. The crude product was detected by LC-MS and next dissolved in DCM (0.9 mL) and TFA (0.1 mL) was added at 0 °C. The reaction was stirred for 30 min and then neutralized with NEt_3. After evaporation of the solvent, the crude PMB-deprotected product was detected by LC-MS and then dissolved in MeOH (1 mL). NaOMe (27.0 mg, 0.5 mmol) was added and the reaction was stirred at room temperature for 2 d. After neutralization with HCl (2 M), the solvent was evaporated *in vacuo*. The residue was dissolved in water, filtered and purified by reversed phase HPLC (0-10 % buffer B, 45 min) to afford the product **11** (1.51 mg, 2.64 µmol, 15% over 5 steps) as colorless solid.

RP-HPLC: R_t (C18-column, 0-10% buffer B, 45 min) = 21.3 min.

^{1}H-NMR (600 MHz, methanol-D_4, depicted in section 4 below): δ = 7.04 (s, 1H, C18H), 6.11 (ddd, J_1 = 6.2 Hz, J_2 = 2.4 Hz, J_3 = 1.9 Hz, 1H, C1H), 6.05 (dd, J_1 = 6.2 Hz, J_2 = 1.5 Hz, 1H, C2H), 5.93 (d, *J* = 6.0 Hz, 1H, C19H), 5.04 (d, *J* = 1.8 Hz, **$^1J_{C-H}$ = 170.3 Hz**, 1H, C6H), 4.76 (m, 1H, C5H), 4.43 (t, *J* = 5.7 Hz, 1H, C20H), 4.26 (t, *J* = 5.4 Hz, 1H, C4H), 4.23 (dd, J_1 = 5.3 Hz, J_2 = 3.6 Hz, 1H, C21H), 4.16 (m, 3H, C3H, C12H), 4.02 (q, *J* = 3.5 Hz, 1H, C22H), 3.93 (dd, J_1 = 3.4 Hz, J_2 = 1.8 Hz, 1H, C7H), 3.91 (m, 1H, C11Ha), 3.80 (dd, J_1 = 12.2 Hz, J_2 = 3.2 Hz, C23Ha), 3.78 (dd, J_1 = 9.4 Hz, J_2 = 3.4 Hz, 1H, C8H), 3.73 (m, 3H, C10H, C11Hb, C233Hb), 3.62 (t, *J* = 9.4 Hz, 1H, C9H), 1.92 (AcO^-) ppm.

^{13}C-NMR (150 MHz, methanol-D_4, depicted in section 4 below): δ = 162.3 (C13), 154.3 (C14), 153.7 (C15), 137.2 (C1), 132.2 (C2), 119.4 (C18), 114.0 (C17), 100.8 (C16), 100.5 (C6), 89.5 (C19), 86.5 (C22), 79.0 (C4), 75.6 (C20), 75.6 (C10), 73.3 (C5), 72.4 (C8), 72.2 (C21), 71.8 (C7), 68.9 (C9), 66.4 (C3), 63.3 (C23), 63.1 (C11), 43.9 (C12), 23.1 ($CO_2CH_3^-$) ppm.

Note: ^{1}H-^{13}C-HMBC spectra of this compound are shown in section 4 below.

HRMS (ESI^-) m/z calc. for $C_{23}H_{32}NO_{12}$, $[M - H]^-$: 570.2053; found: 570.2052.

α-*allyl*-mannosyl-queuosine (4)

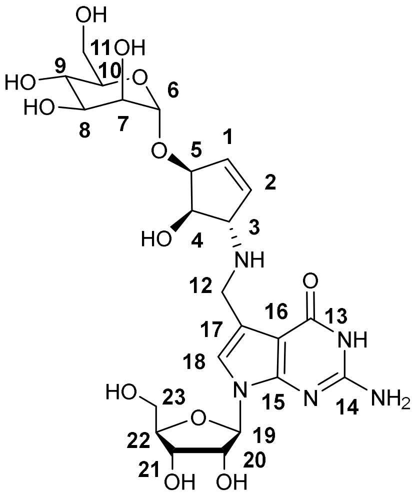

19 (16.0 mg, 14.4 µmol) was dissolved in 10% $HNMe_2$ in THF (1 mL) and the reaction was stirred for 1 h. The solvent was evaporated in vacuo to give the free amine, which was detected by LC-MS. The amine was dissolved in MeOH (0.5 mL) and **8** (10.2 mg, 14.4 µmol) was added together with AcOH (2.00 µL). The reaction was stirred for 5 h before it was cooled to 0 °C and $NaBH_4$ (1.42 mg, 37.4 µmol) was added. The reaction was stirred at 0 °C for 30 min before H_2O (0.3 mL) was added and the reaction was stirred for another 5 min. The solvent was evaporated *in vacuo* and the crude product was detected by LC-MS. The residue was dissolved in MeOH (2 mL) and NaOMe (24.1 mg, 1.00 mmol) was added. The reaction was stirred at room temperature for 18 h and then neutralized with DOWEX-H^+resin. The resin was filtered off and the solvent was evaporated. The residue was dissolved in DCM (1.8 mL) and TFA (0.2 mL) was added at 0 °C. The reaction was stirred at 0 °C for 5 min before the reaction was stopped by addition of a solution of $HNEt_3OAc$ in DCM (1 M, 3 mL). Saturated aqueous $NaHCO_3$-solution was added and the phases were separated. The aqueous phase containing the product was evaporated *in vacuo*. The sample was desalted using a SepPak-C18-cartridge. The sample was loaded with 100% water and the product was eluted from the column with a mixture of MeCN and water (50/50 v/v). The solvent was evaporated by lyophilization. The residue was dissolved in MeCN (1 mL) and HF · pyridine (100 µL) was added. The mixture was stirred for 1 week at room temperature before TMSOMe (1.5 mL) were added. The mixture was stirred for 1 h at room temperature before the solvent was evaporated *in vacuo*. The residue was dissolved in water, filtered and purified by reversed phase HPLC (0-10 % buffer B, 45 min) to afford the product **4** (0.24 mg, 0.42 µmol, 2.9% over 6 steps) as colorless solid.

RP-HPLC: R_t (C18-column, 0-10% buffer B, 45 min) = 21.4 min.

^{1}H-NMR (800 MHz, D_2O, depicted in section 4 below): δ = 6.95 (s, 1H, C18H), 6.16 (d, J = 6.3 Hz, 1H, C1H), 6.10 (dd, J_1 = 6.3 Hz, J_2 = 1.8 Hz, 1H, C2H), 5.96 (d, J = 6.6 Hz, 1H, C19H), 4.96 (d, J = 1.8 Hz, $\mathbf{^1J_{C\text{-}H}}$ **= 170.0 Hz**, 1H, C6H), 4.68 (m, 1H, C5H), 4.58 (dd, J_1 = 5.4 Hz, J_2 = 3.2 Hz, 1H, C20H), 4.32 (dd, J_1 = 5.4 Hz, J_2 = 3.2 Hz, 1H, C21H), 4.15 (q, J = 3.8 Hz, 1H, C22H), 4.13 (t, J = 5.2 Hz, 1H, C4H), 3.97 (d, J = 13.9 Hz, 1H, C12Ha), 3.94 (d, J = 13.9 Hz, 1H, C12Hb), 3.90 (dd, J_1

= 3.4 Hz, J_2 = 1.8 Hz, 1H, C7H), 3.84 (m, 3H, C3H, C8H, C11Ha), 3.81 (dd, J_1 = 12.5 Hz, J_2 = 3.4 Hz, 1H, C23Ha), 3.76 (m, 3H, C10H, C11Hb, C23Hb), 3.65 (t, J = 9.7 Hz, 1H, C9H) ppm.

Note: A ^{1}H-NMR spectrum (800 MHz) of this compound in **methanol-D$_4$** is depicted at the end of section 4 below.

^{13}C-NMR (HSQC, HMBC, D_2O): δ = 153.2 (C14/C15), 151.7 (C14/C15), 136.1 (C2), 130.8 (C1), 116.8 (C18), 115.5 (C17), 100.8 (C16), 97.9 (C6), 86.5 (C19), 84.6 (C22), 77.7 (C5), 75.7 (C4), 73.1 (C20), 72.9 (C10), 70.5 (C21), 70.4 (C8), 70.2 (C7), 66.8 (C9), 66.5 (C3), 61.5 (C23), 60.8 (C11), ppm.

Note: ^{1}H-^{13}C-HSQC and -HMBC spectra of this compound are shown in section 4 below.

HRMS (ESI$^+$) m/z calc. for $C_{23}H_{34}N_5O_{12}$, $[M+H]^+$: 572.2199; found: 572.2195.

4. NMR-spectra of important compounds

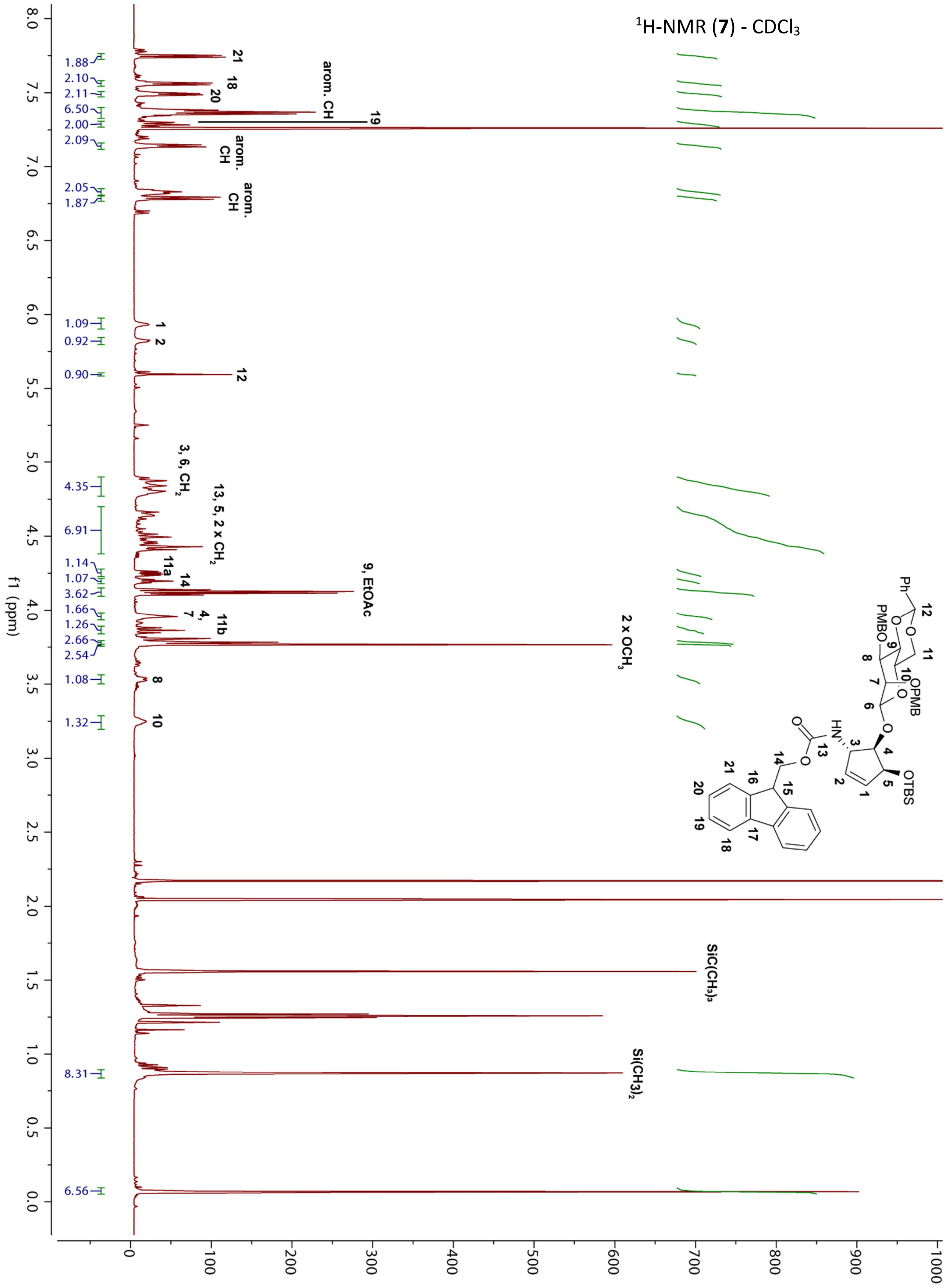

^{13}C-NMR (**7**) - $CDCl_3$

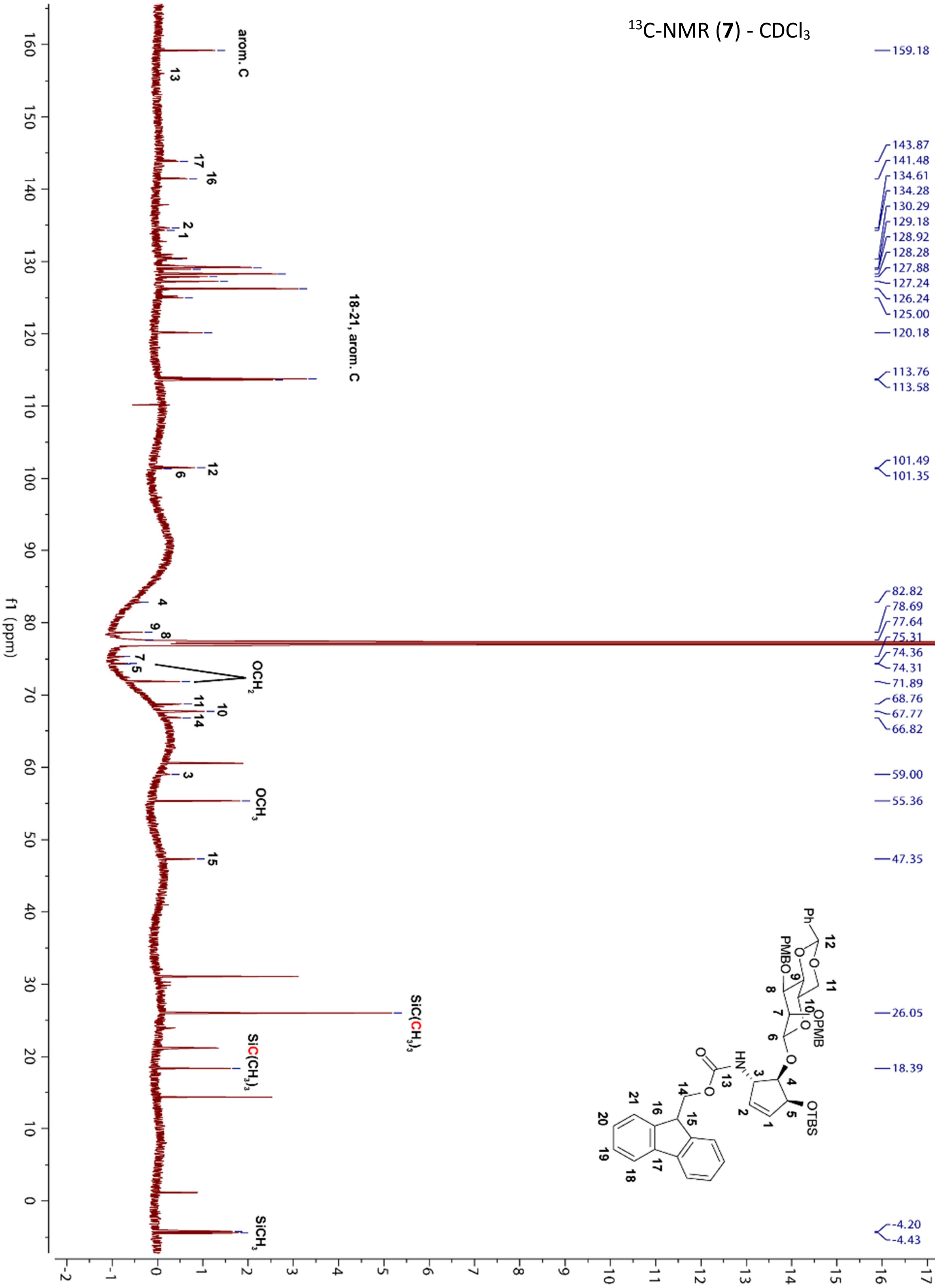

^{1}H-NMR (**3**) – methanol-D$_4$

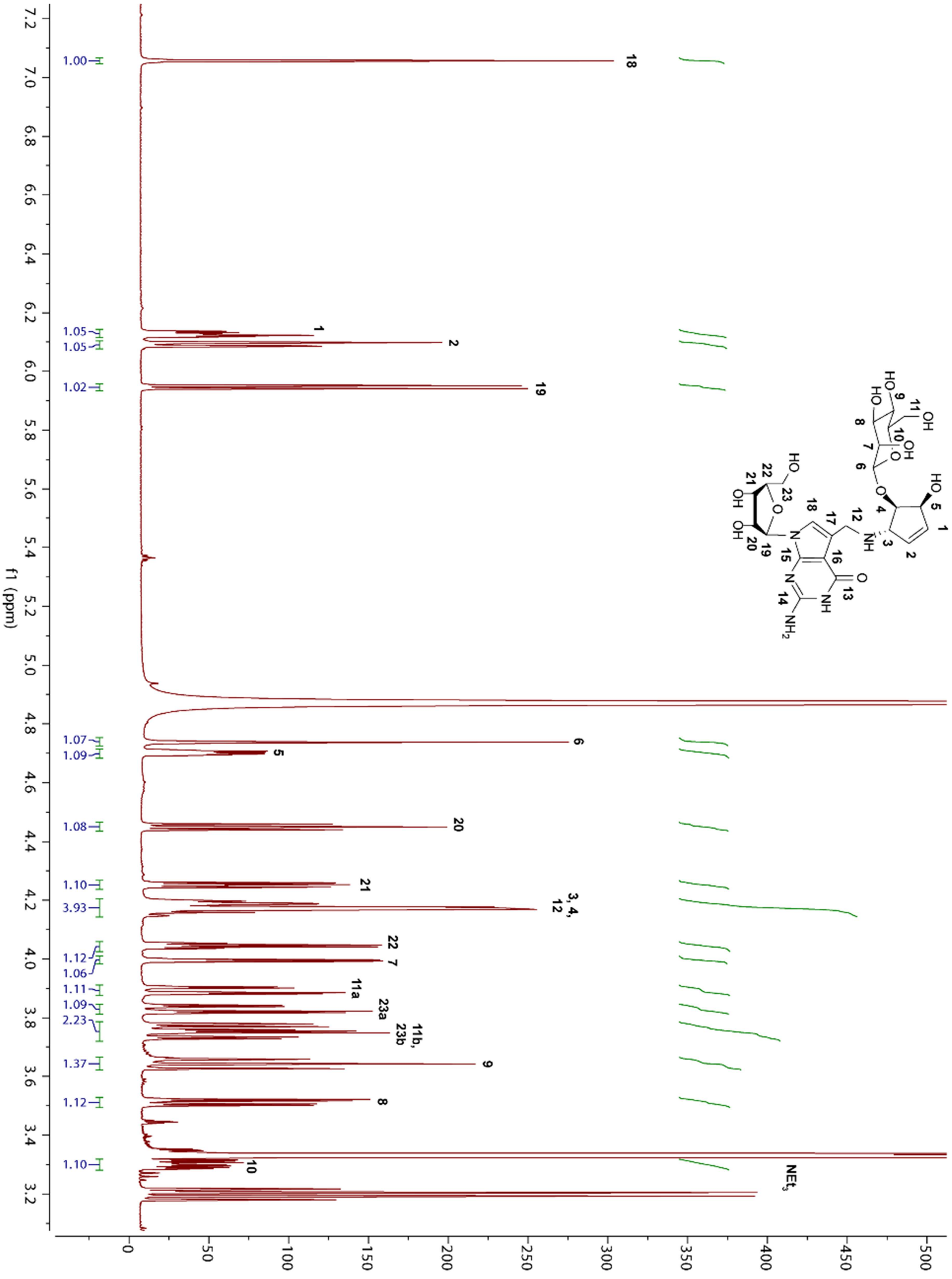

^{13}C-NMR (**3**) - methanol-D$_4$

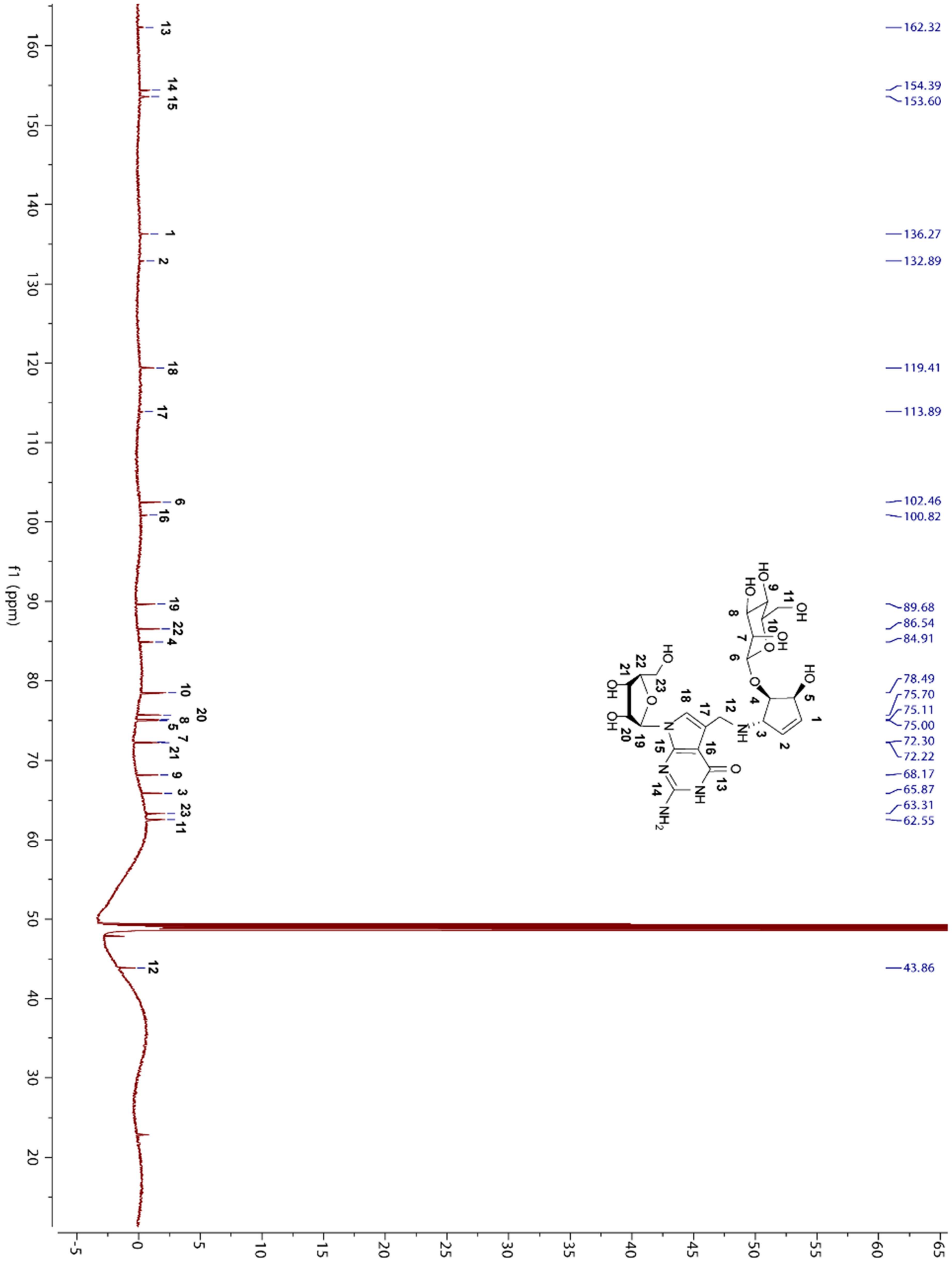

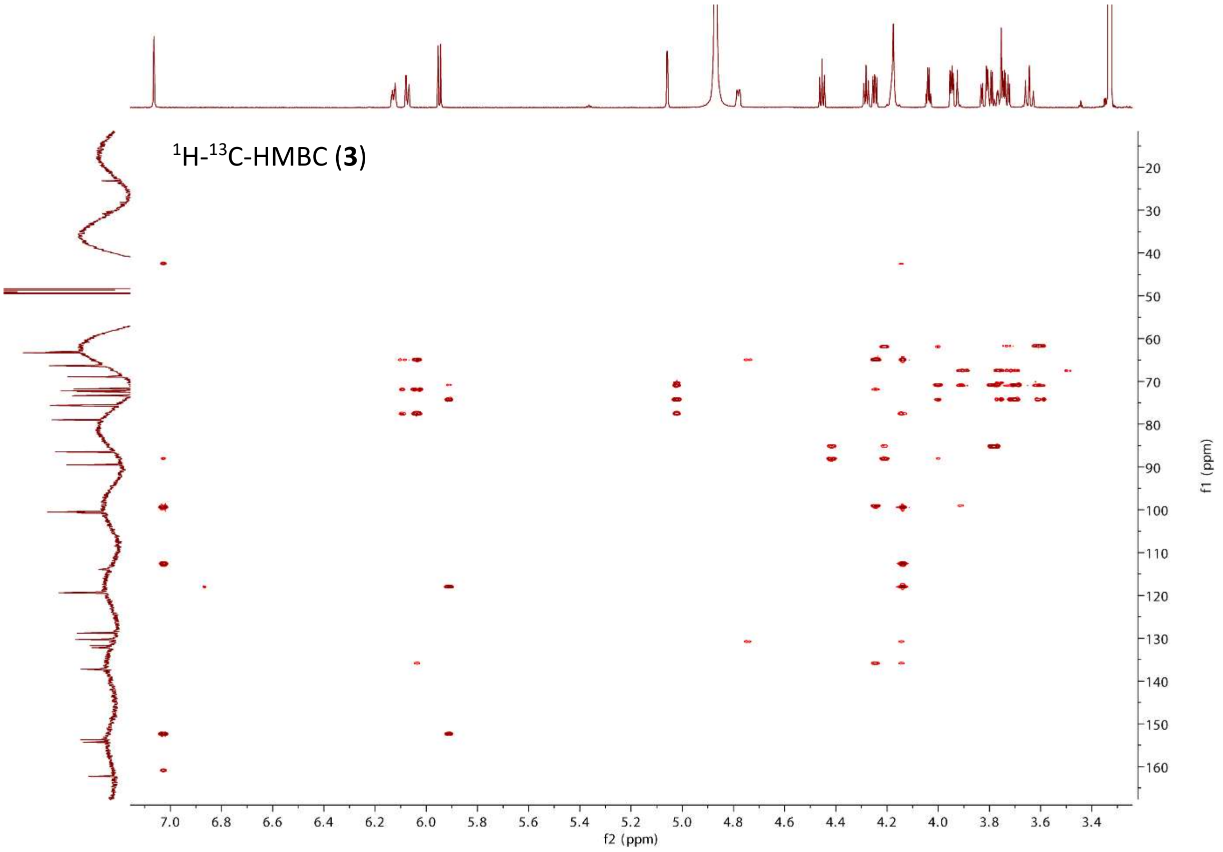
^{1}H-^{13}C-HMBC (**3**)
f1 (ppm)
f2 (ppm)

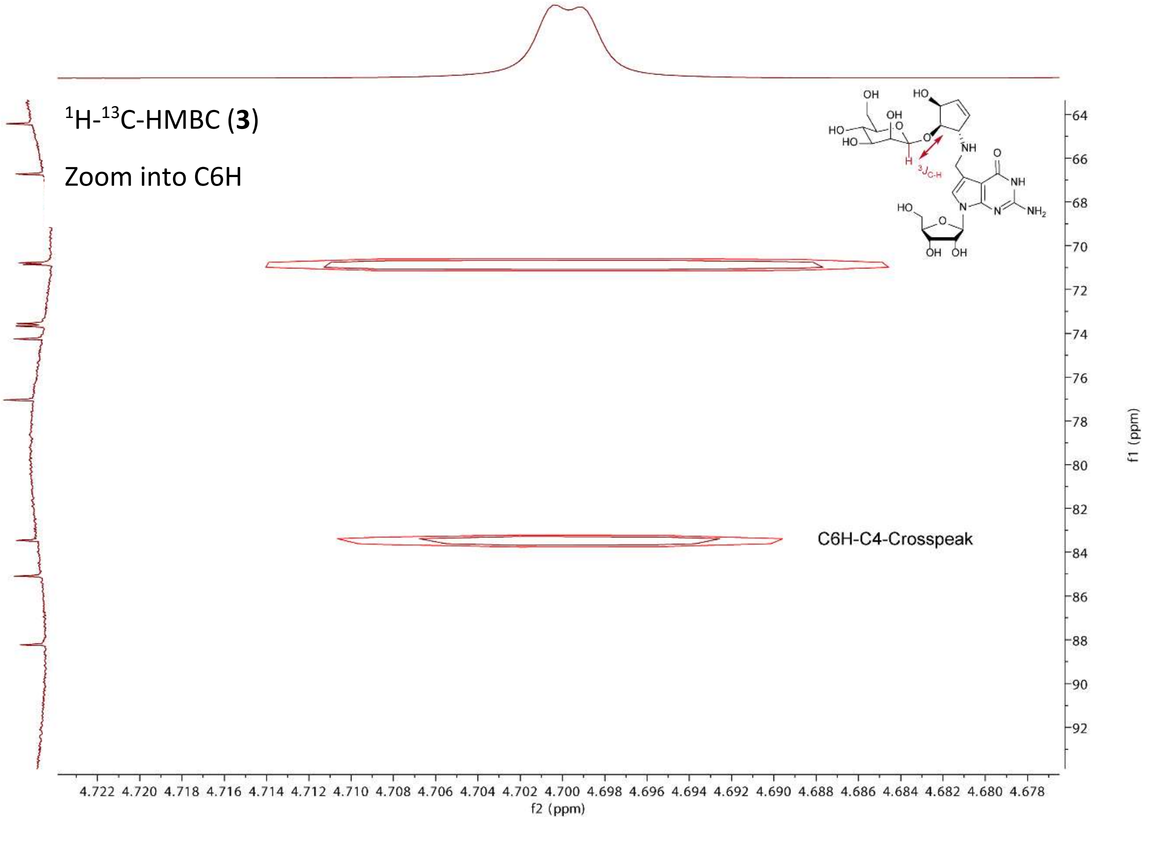
^{1}H-^{13}C-HMBC (**3**)
Zoom into C6H
C6H-C4-Crosspeak
f1 (ppm)
f2 (ppm)

^{1}H-NMR (**29**) – $CDCl_3$

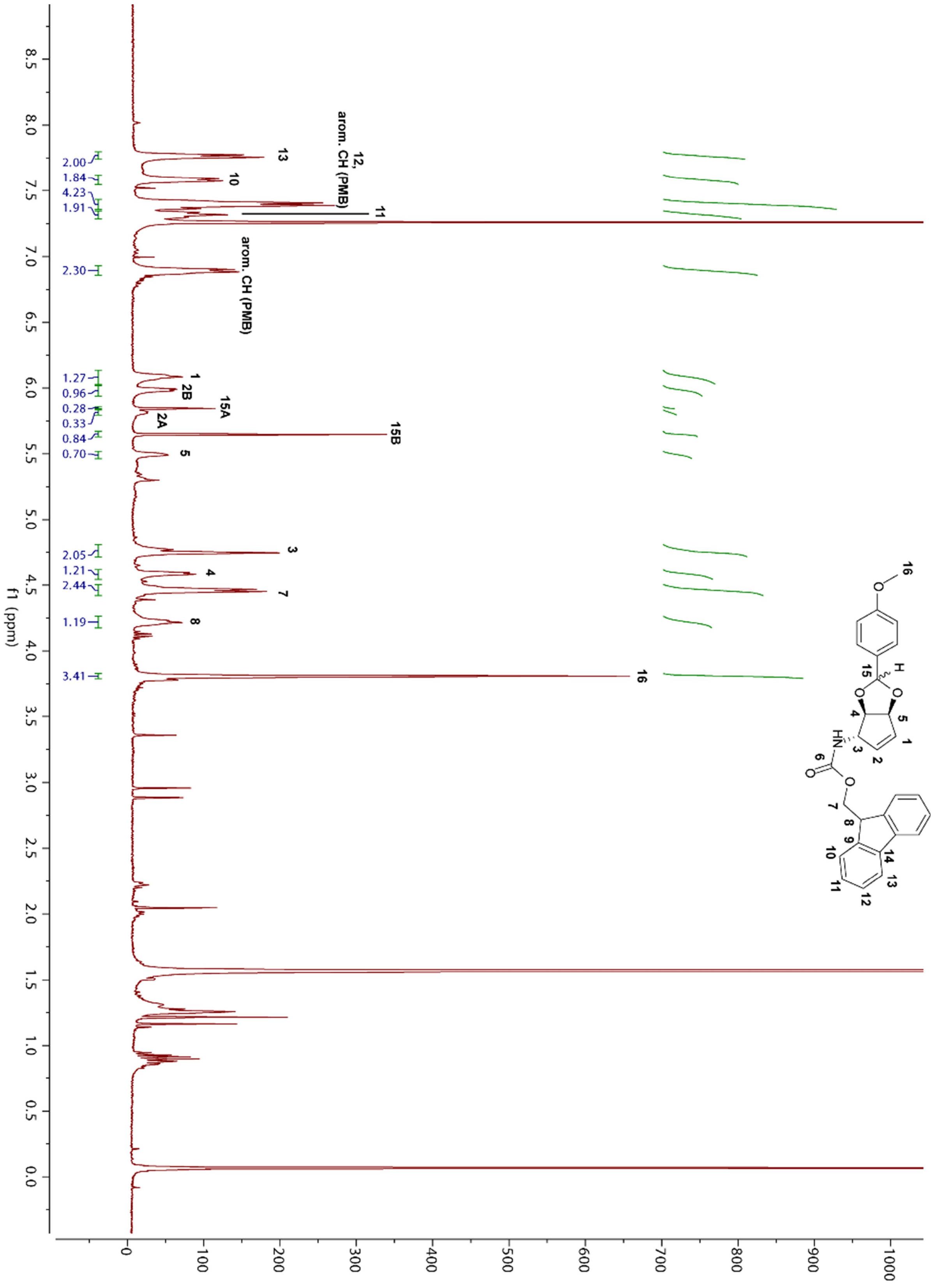

^{13}C-NMR (**29**) – $CDCl_3$

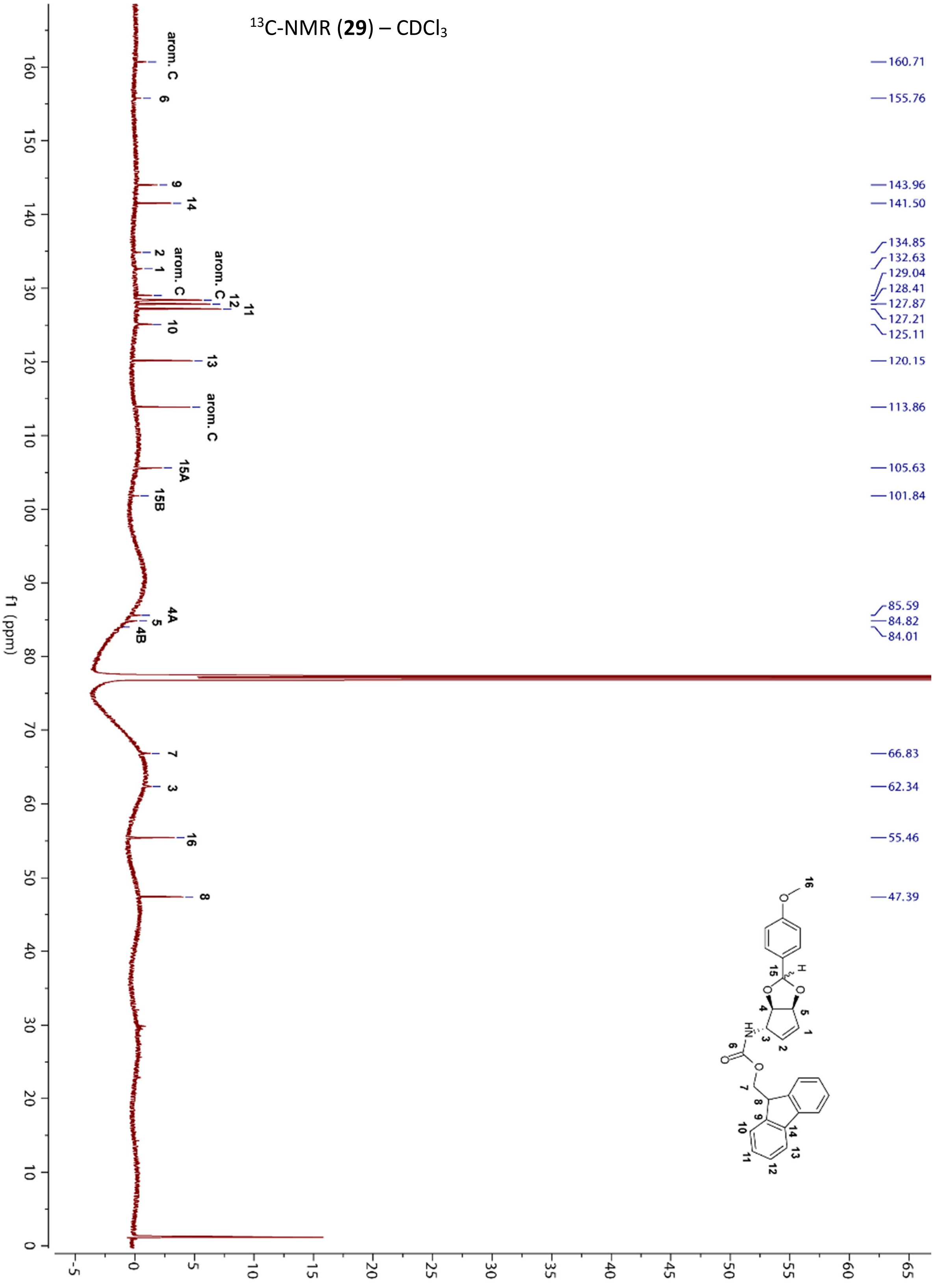

^{1}H-NMR (**13**) – $CDCl_3$

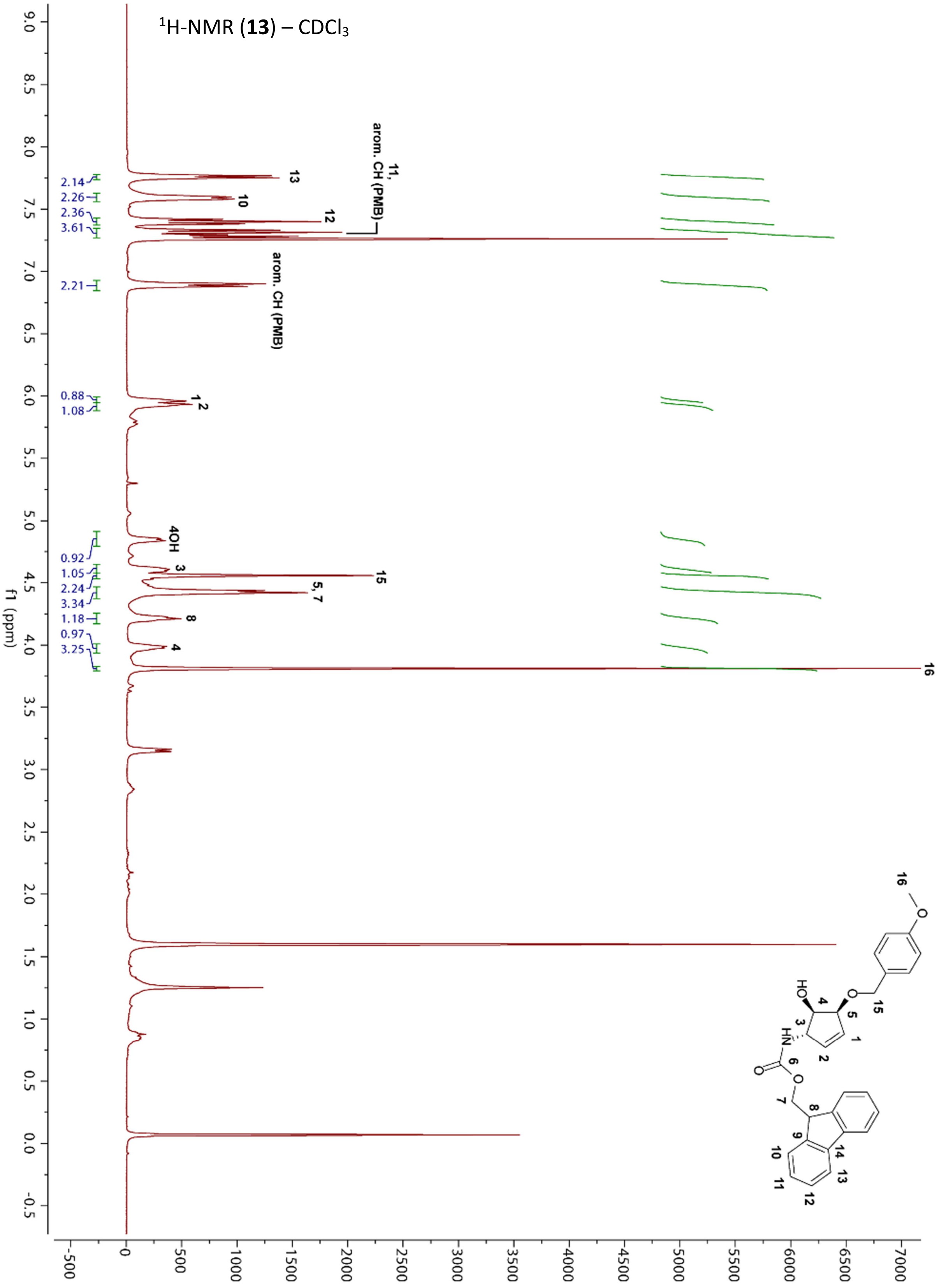

^{13}C-NMR (**13**) – CDCl$_3$

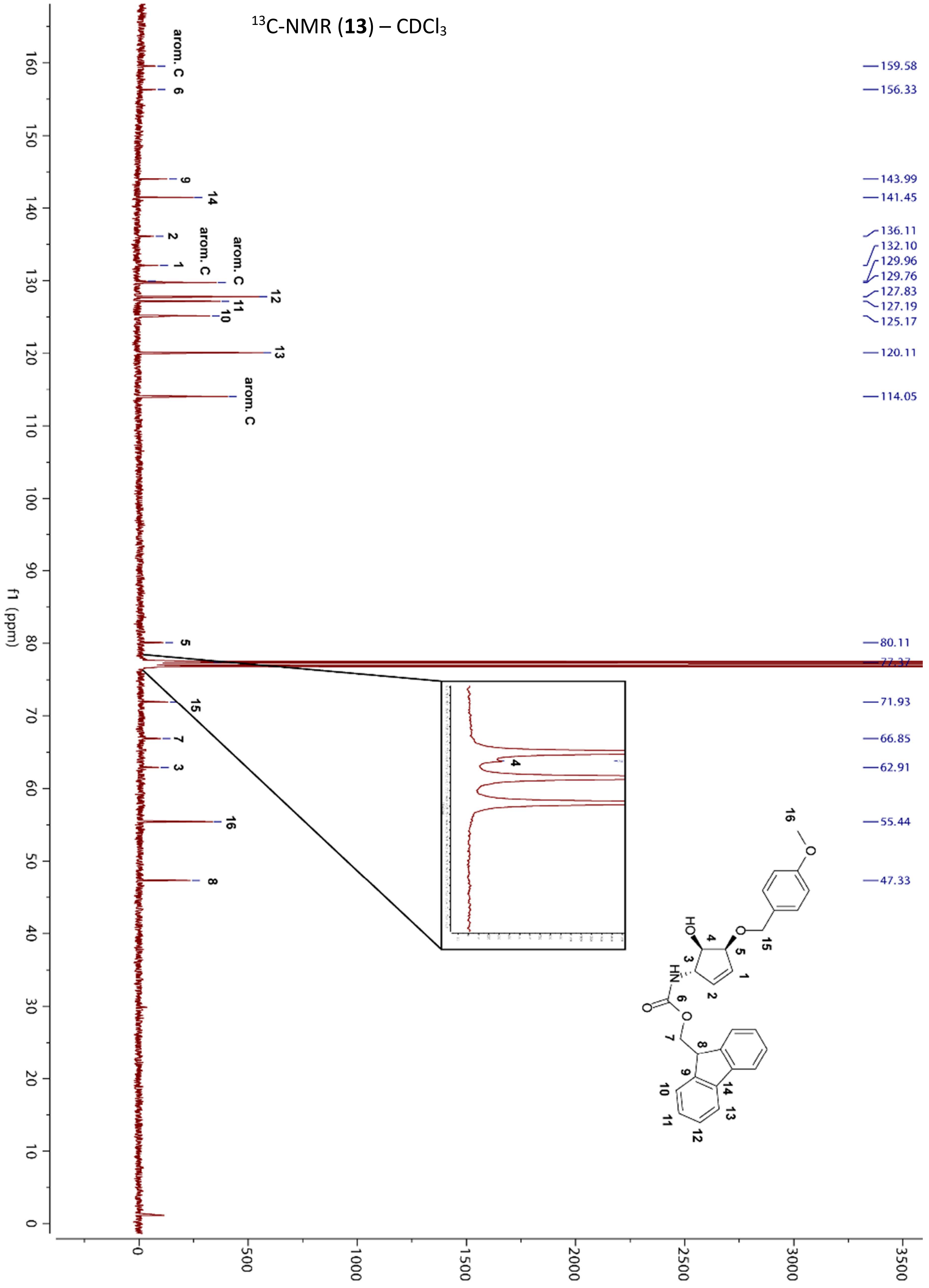

^{1}H-NMR (**30**) – $CDCl_3$

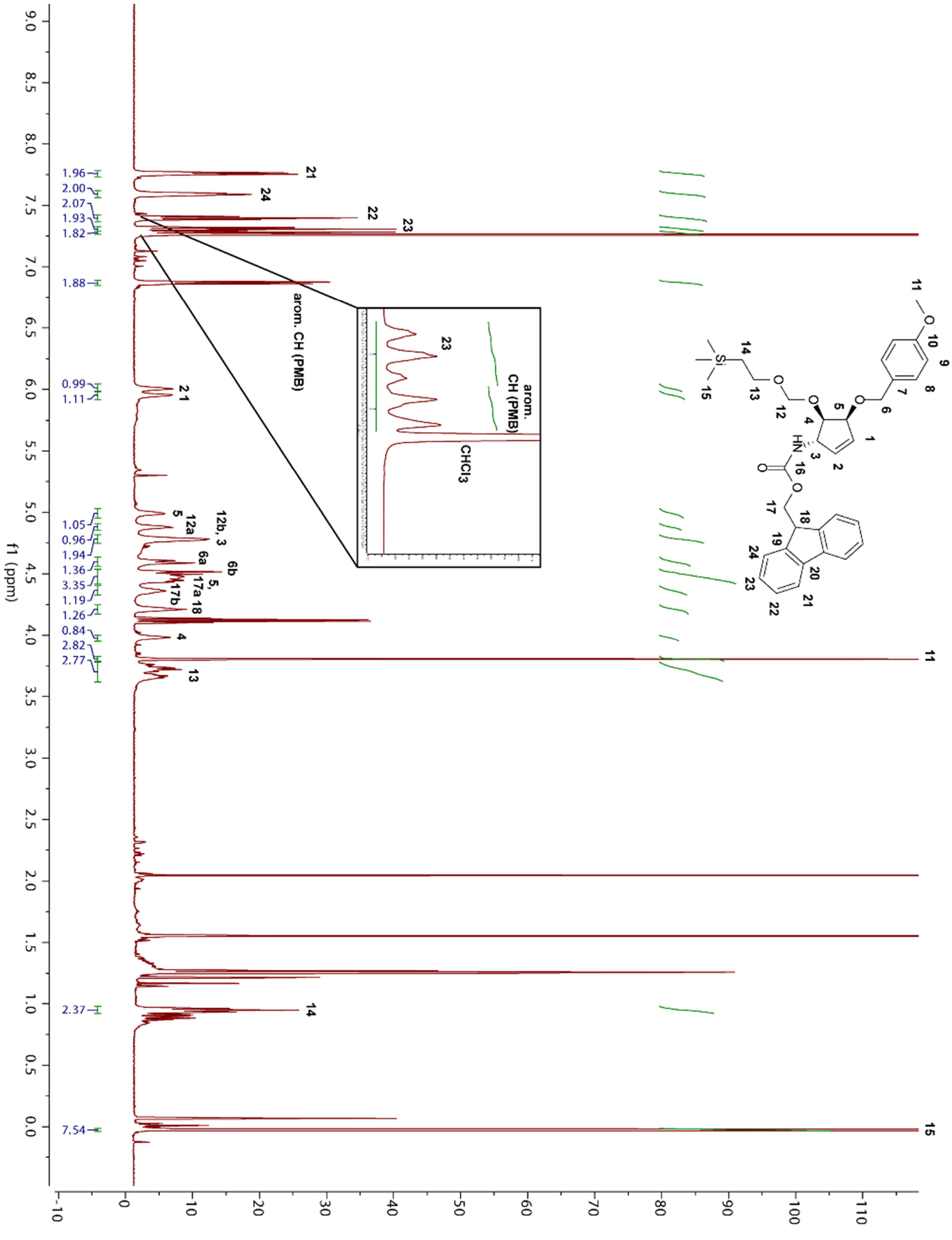

^{13}C-NMR (**30**) – $CDCl_3$

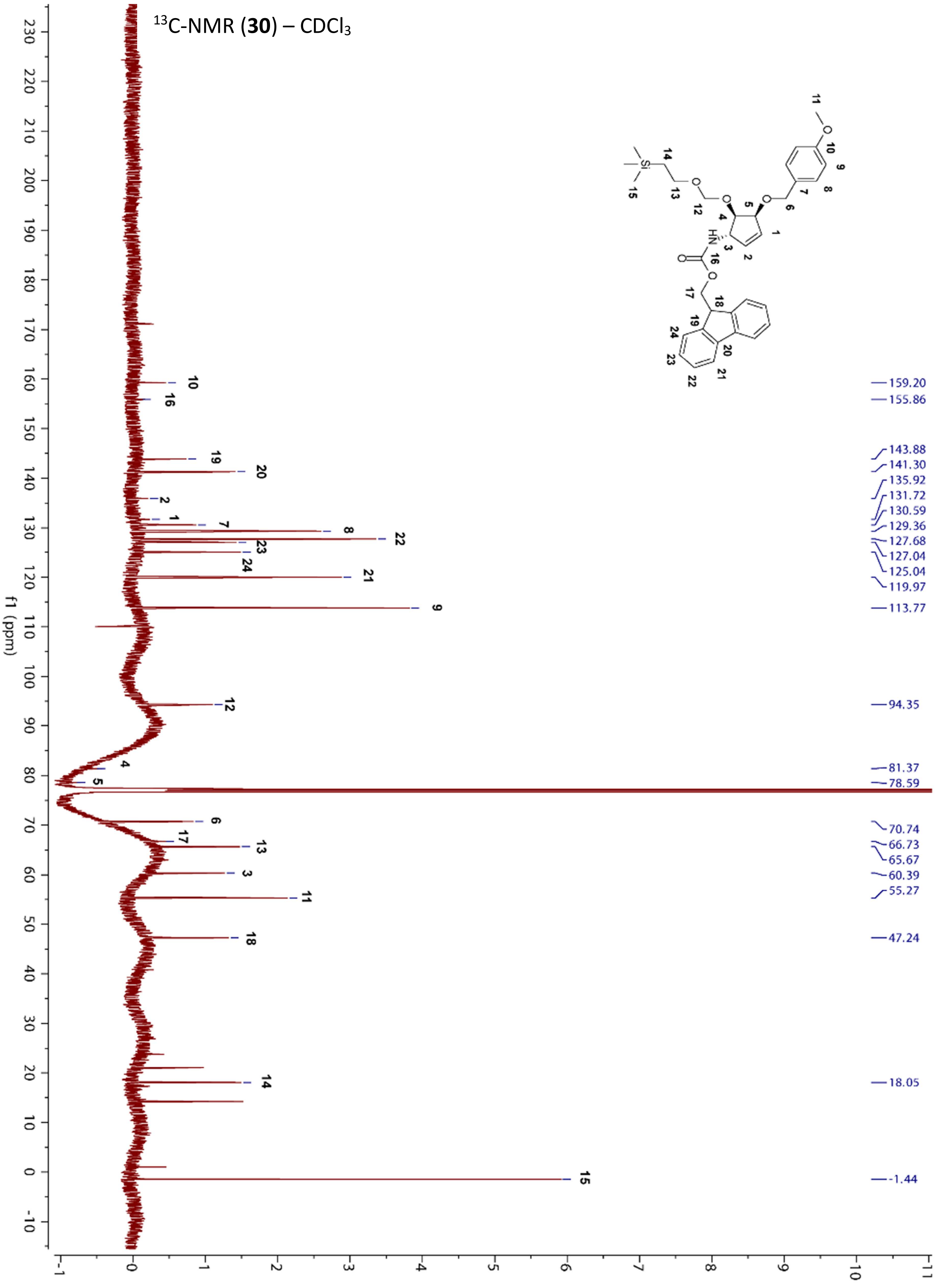

^{1}H-NMR (**14**) – $CDCl_3$

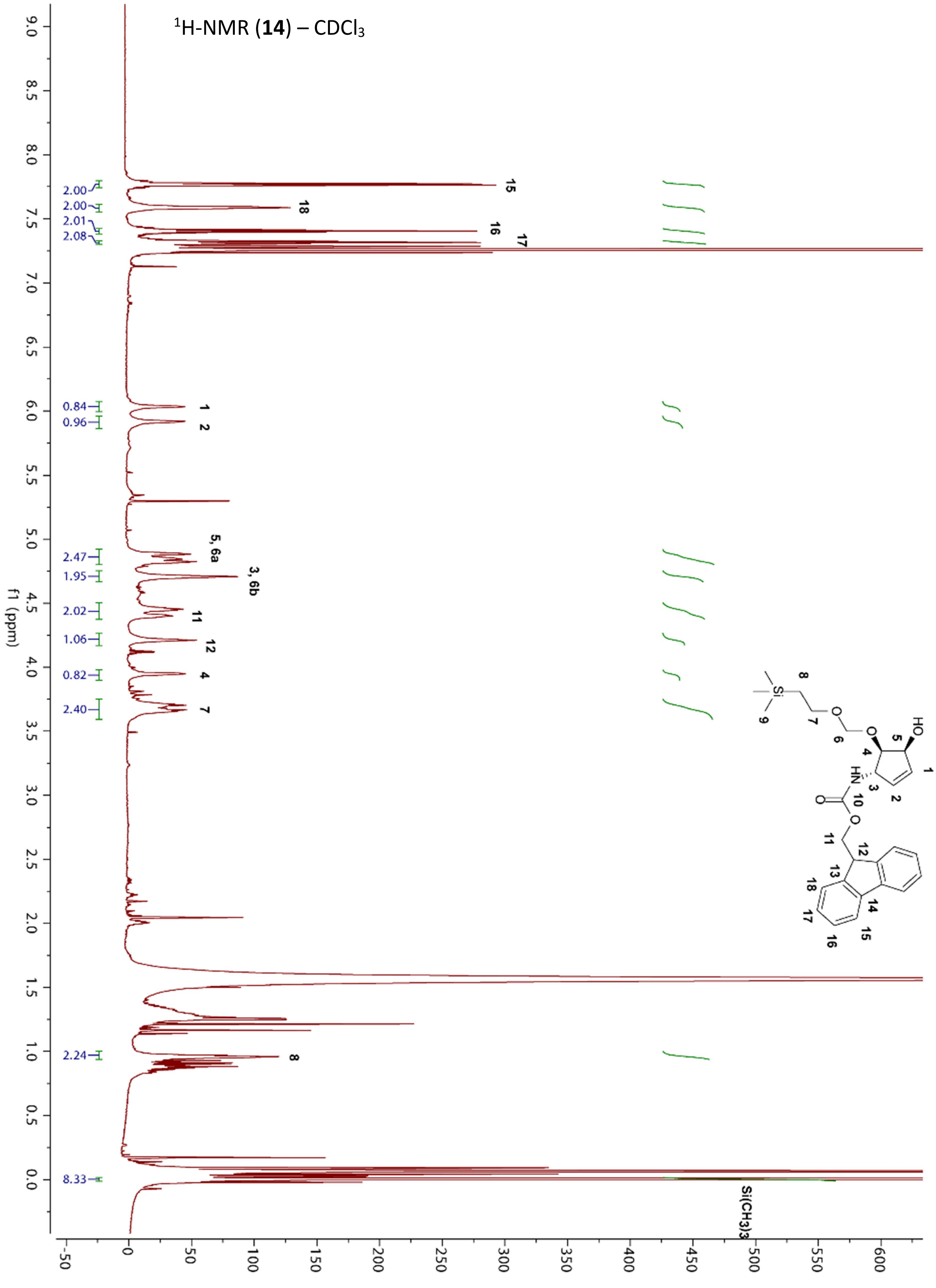

^{13}C-NMR (**14**) – $CDCl_3$

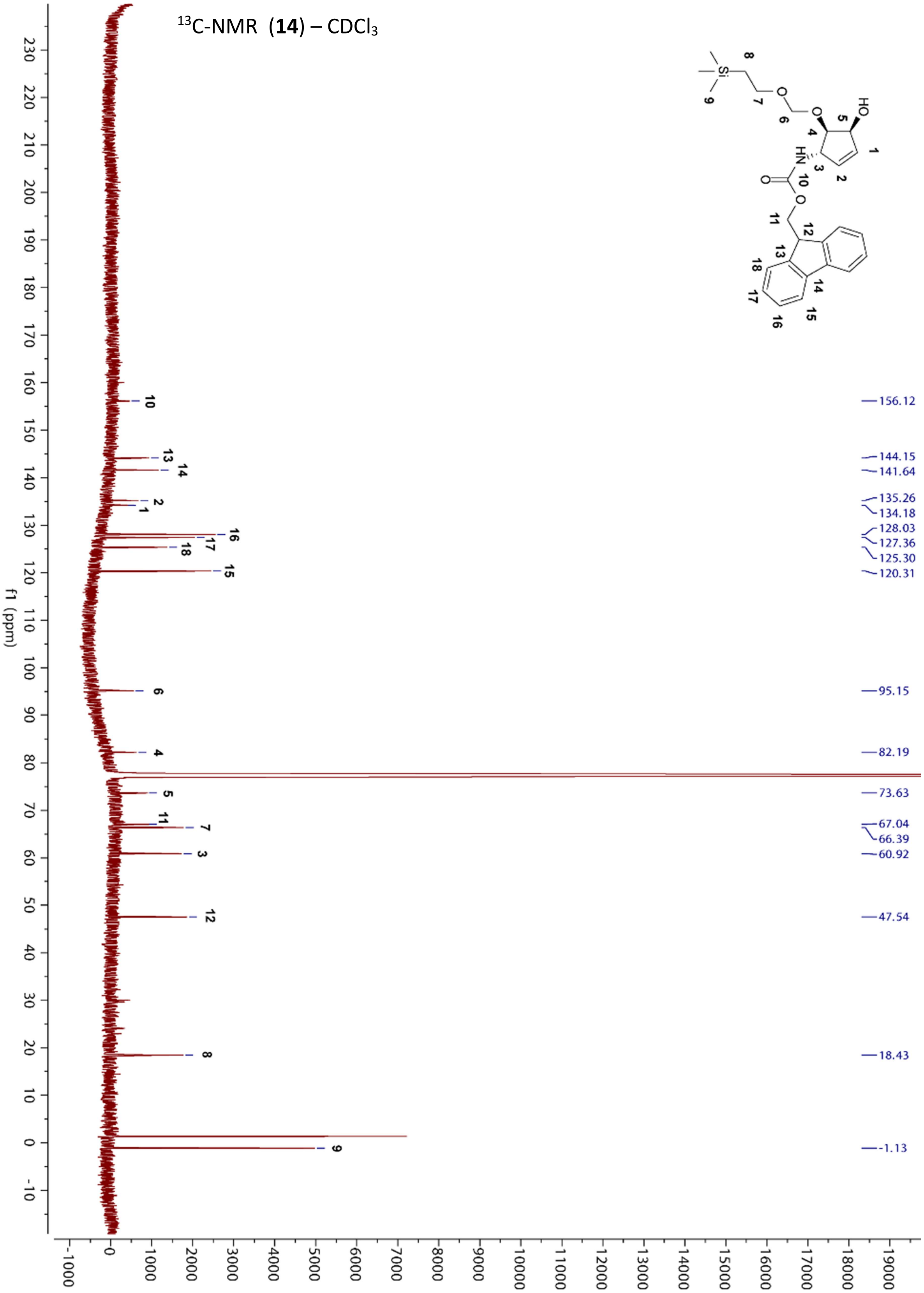

^{1}H-NMR (**15**) – $CDCl_3$

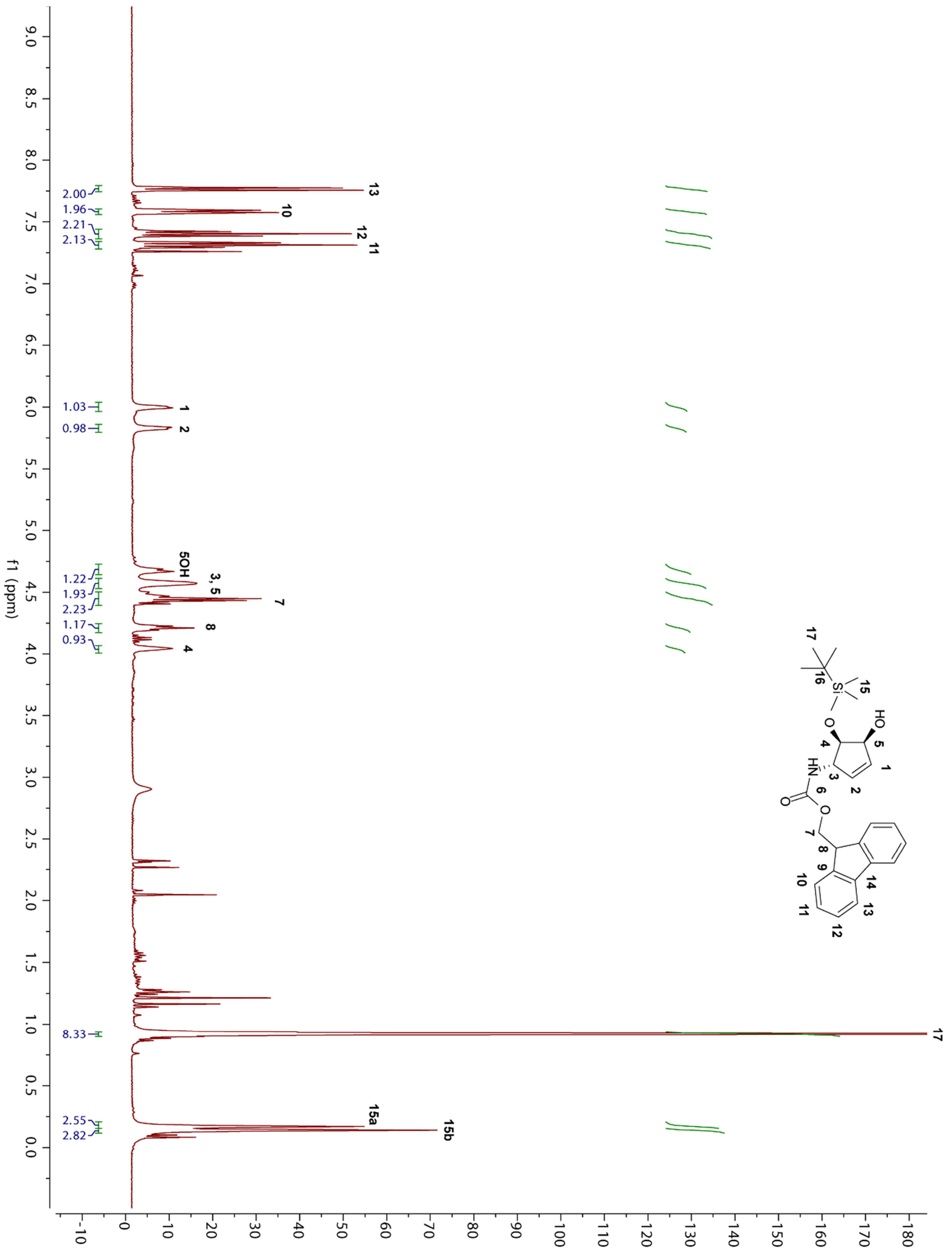

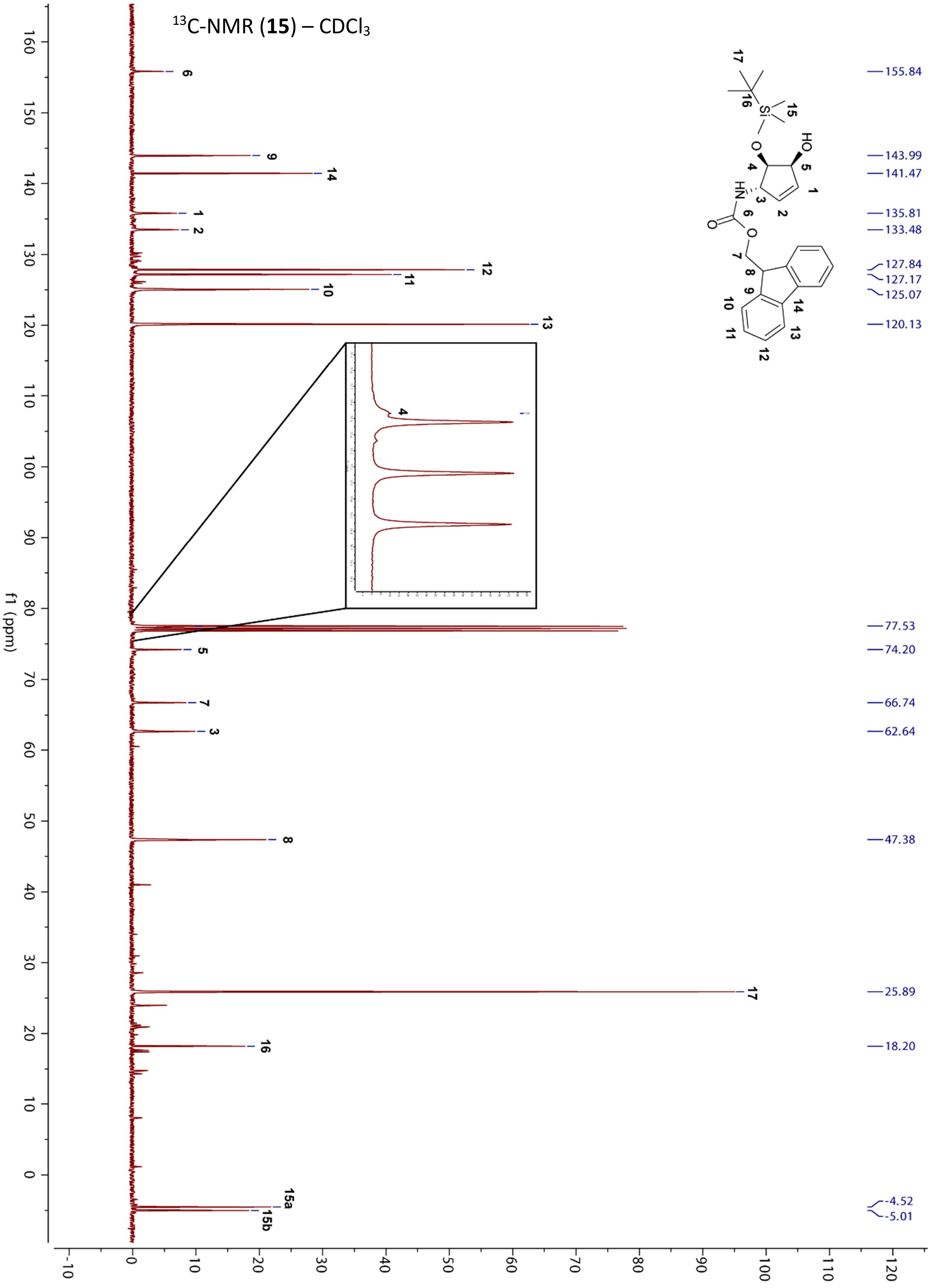
^{13}C-NMR (**15**) – $CDCl_3$
155.84
143.99
141.47
135.81
133.48
127.84
127.17
125.07
120.13
77.53
74.20
66.74
62.64
47.38
25.89
18.20
-4.52
-5.01
f1 (ppm)

^{29}Si-NMR (**15**) – $CDCl_3$

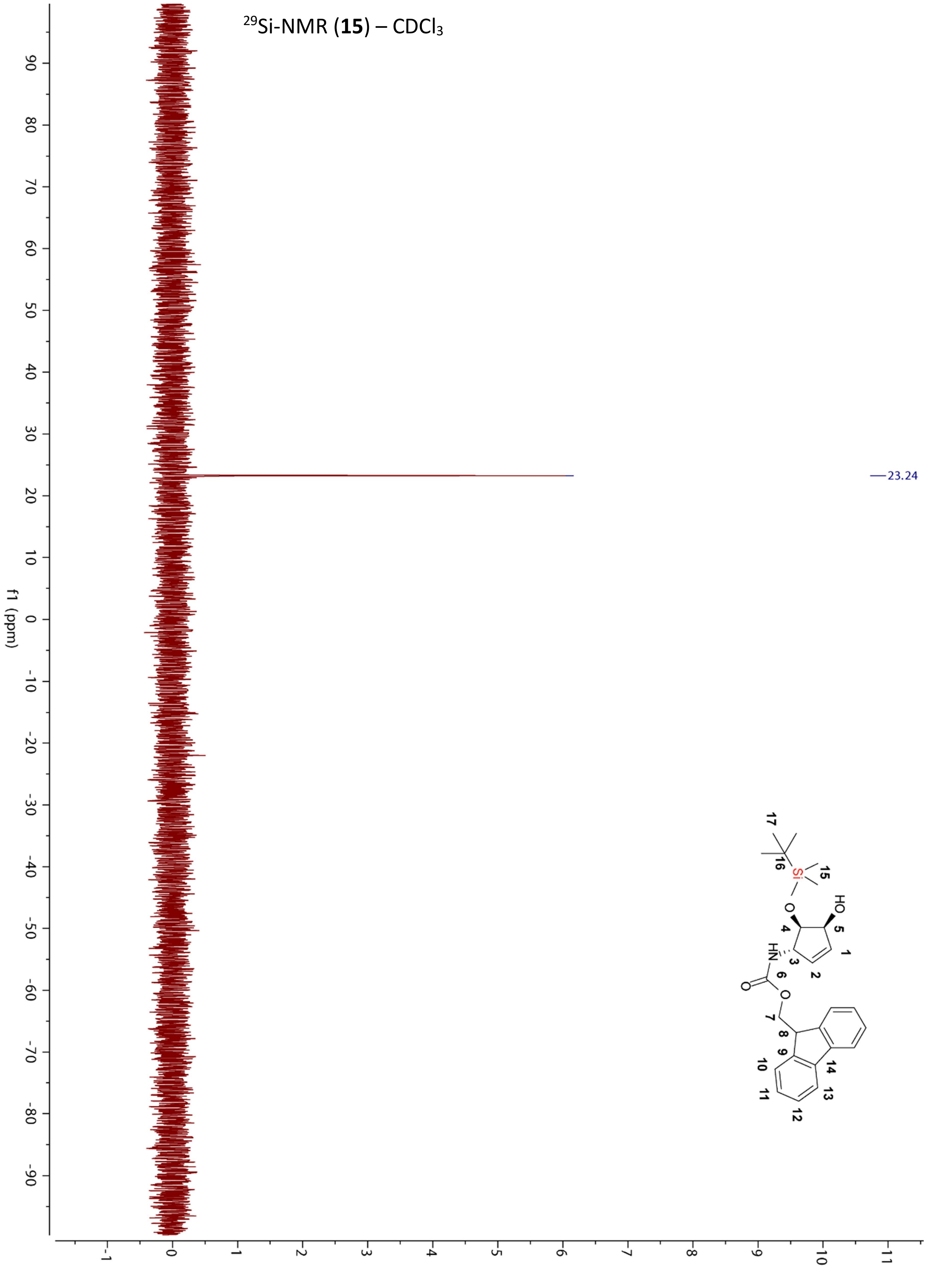

^{1}H-NMR (**17**) – $CDCl_3$

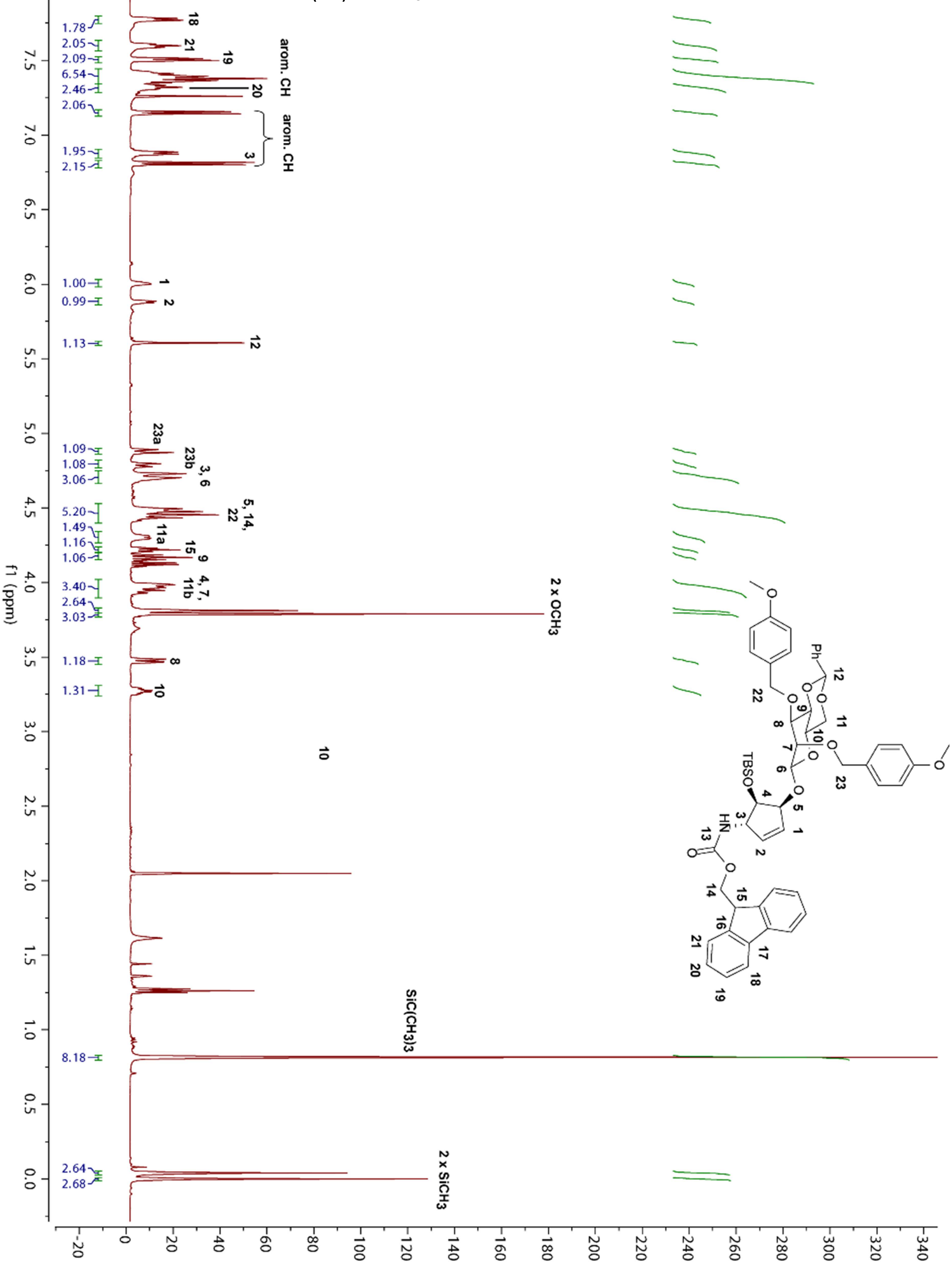

^{13}C-NMR (**17**) – $CDCl_3$

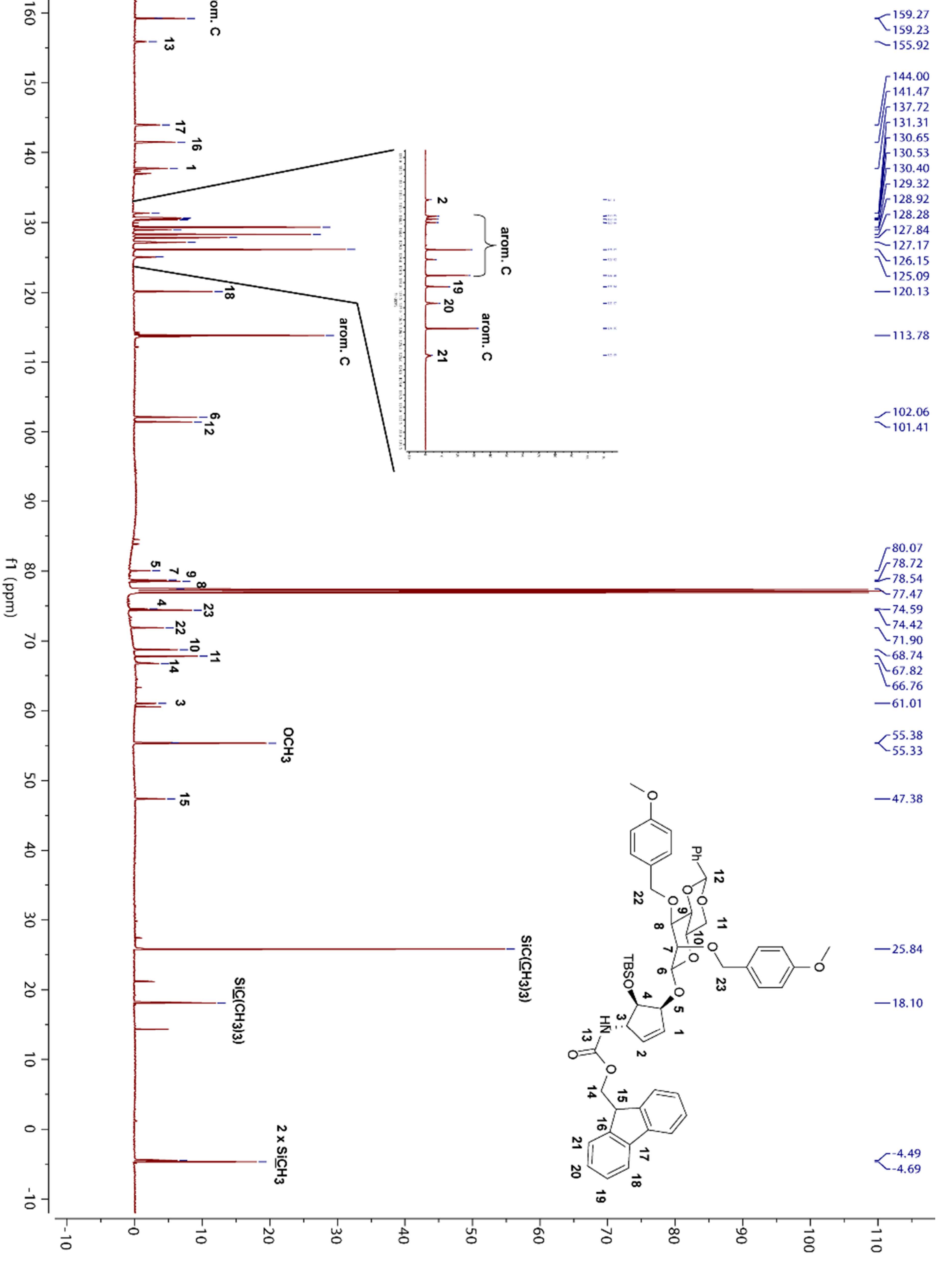

^{1}H-NMR (**18**) – $CDCl_3$

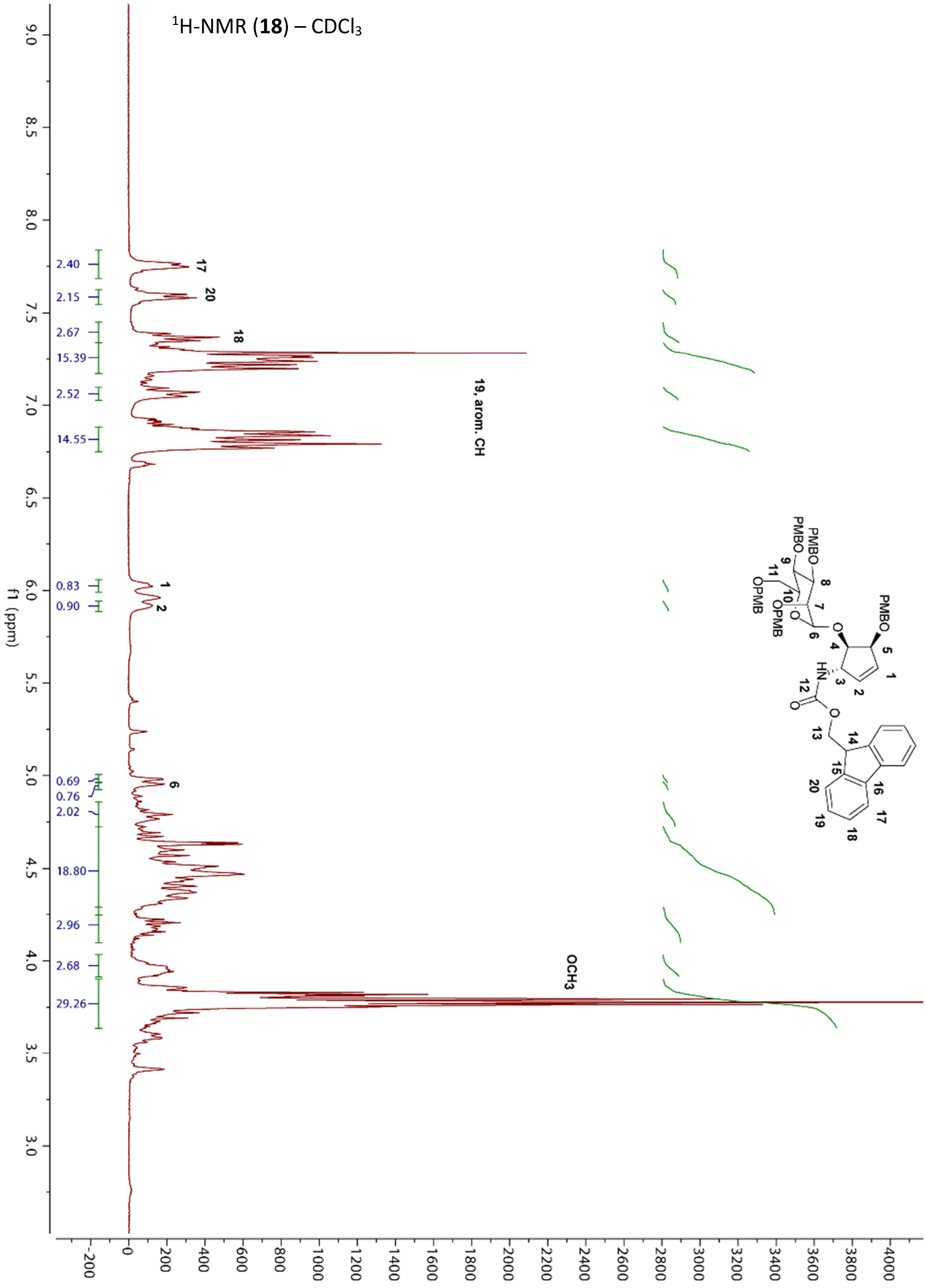

^{13}C-NMR (**18**) – $CDCl_3$

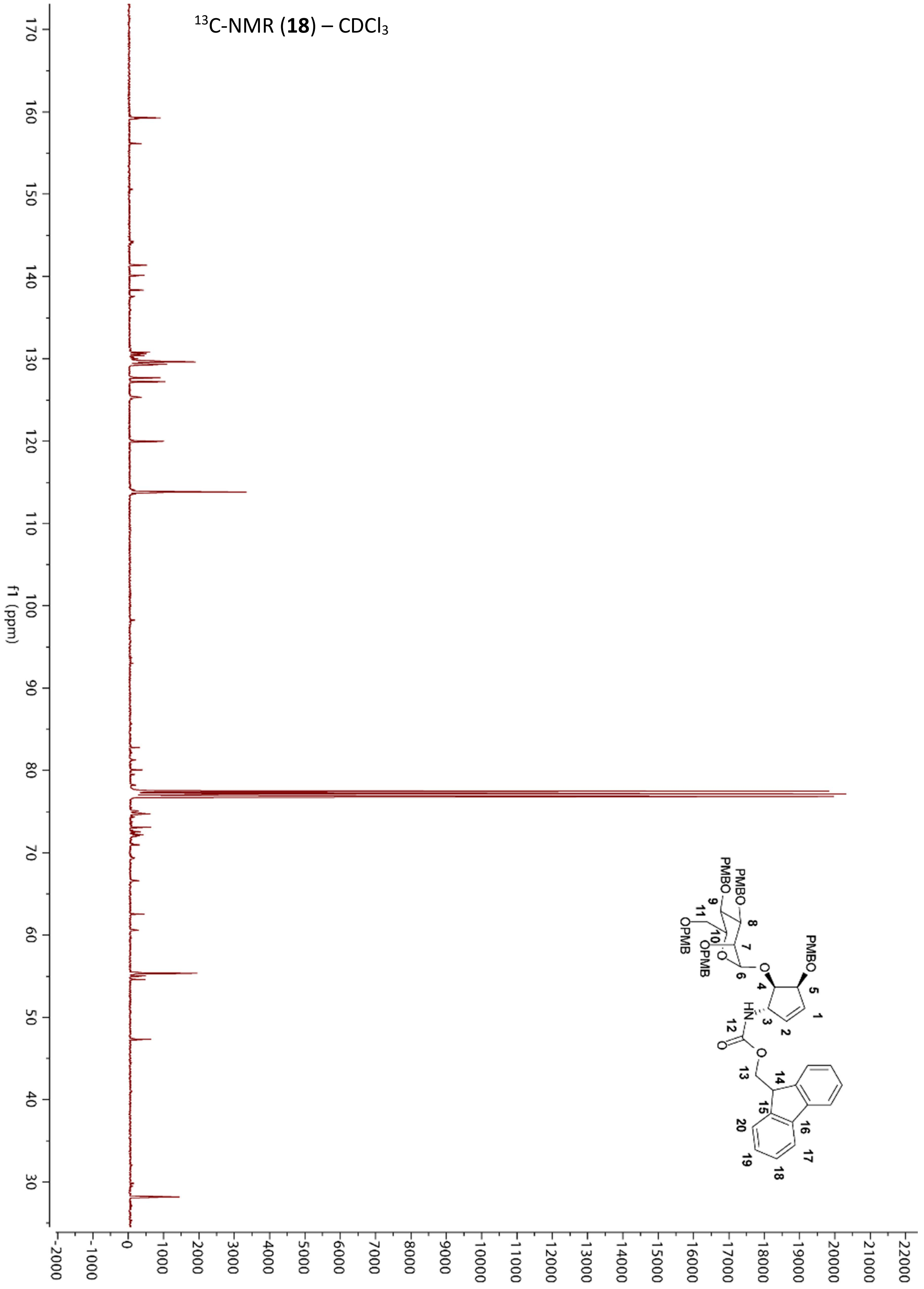

^{1}H-NMR (**19**) – $CDCl_3$

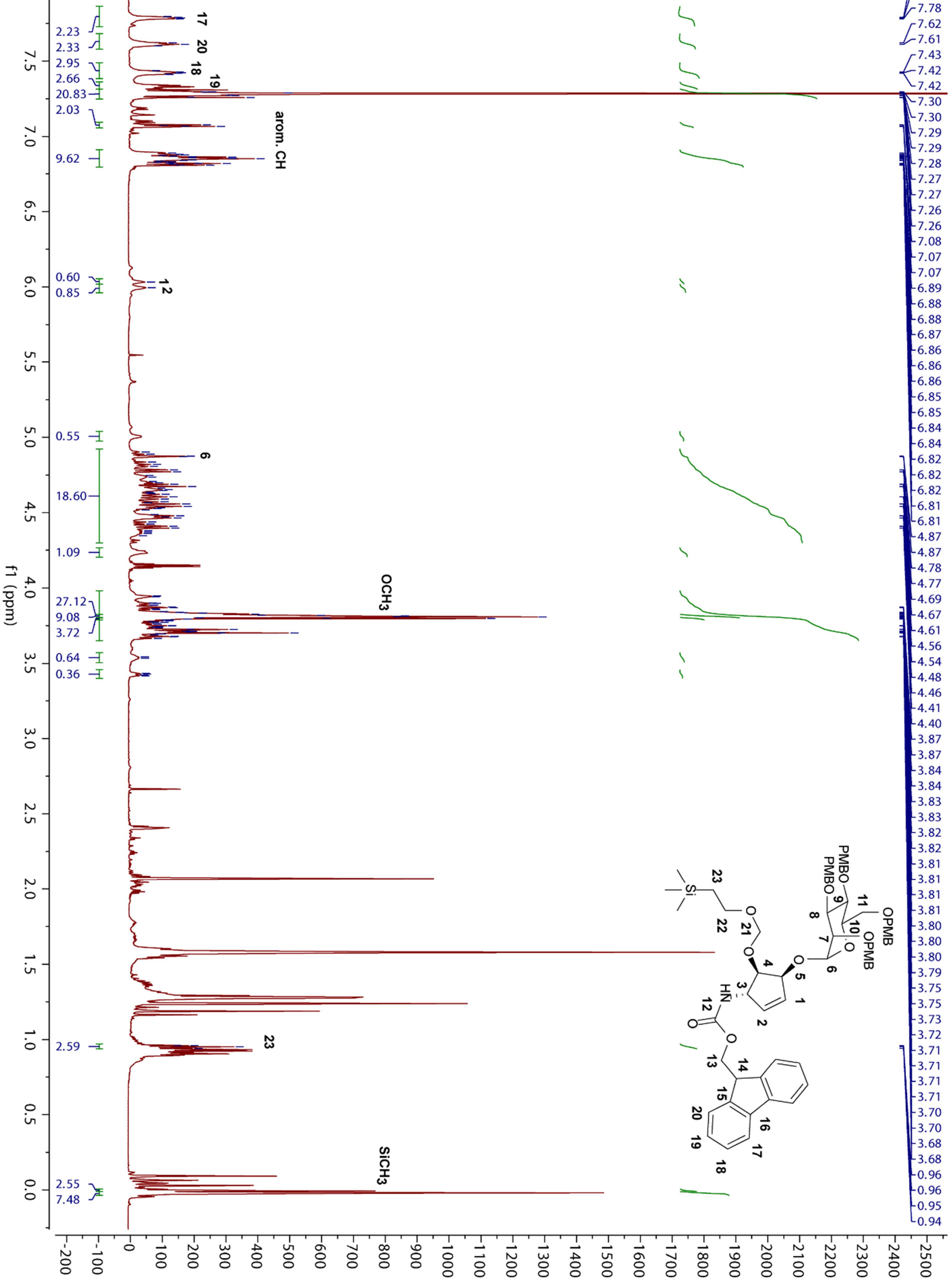

^{13}C-NMR (**19**) – $CDCl_3$

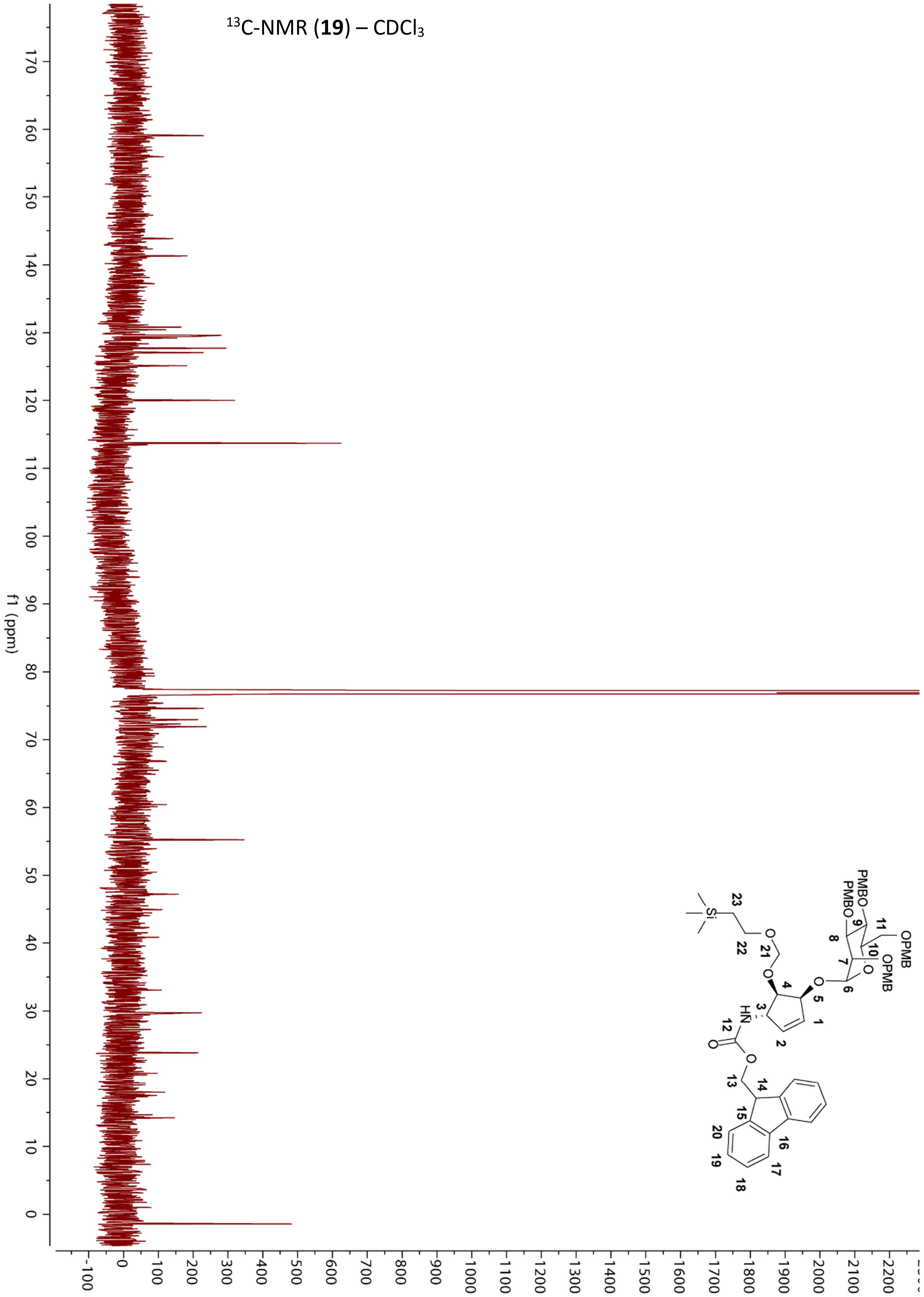

^{1}H-NMR (**10**) – D_2O

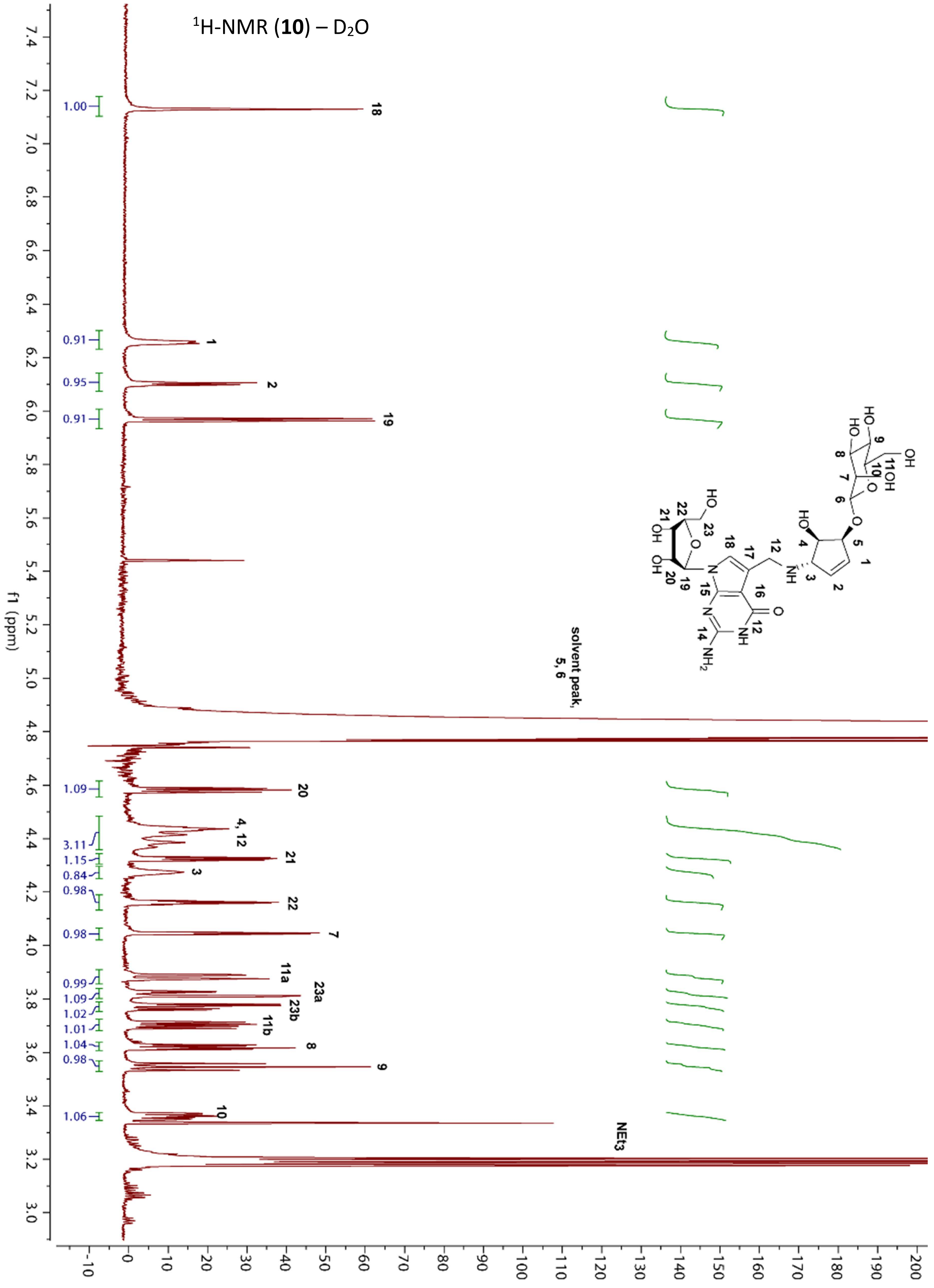

^{1}H-^{13}C-HSQC (**10**) – D_2O

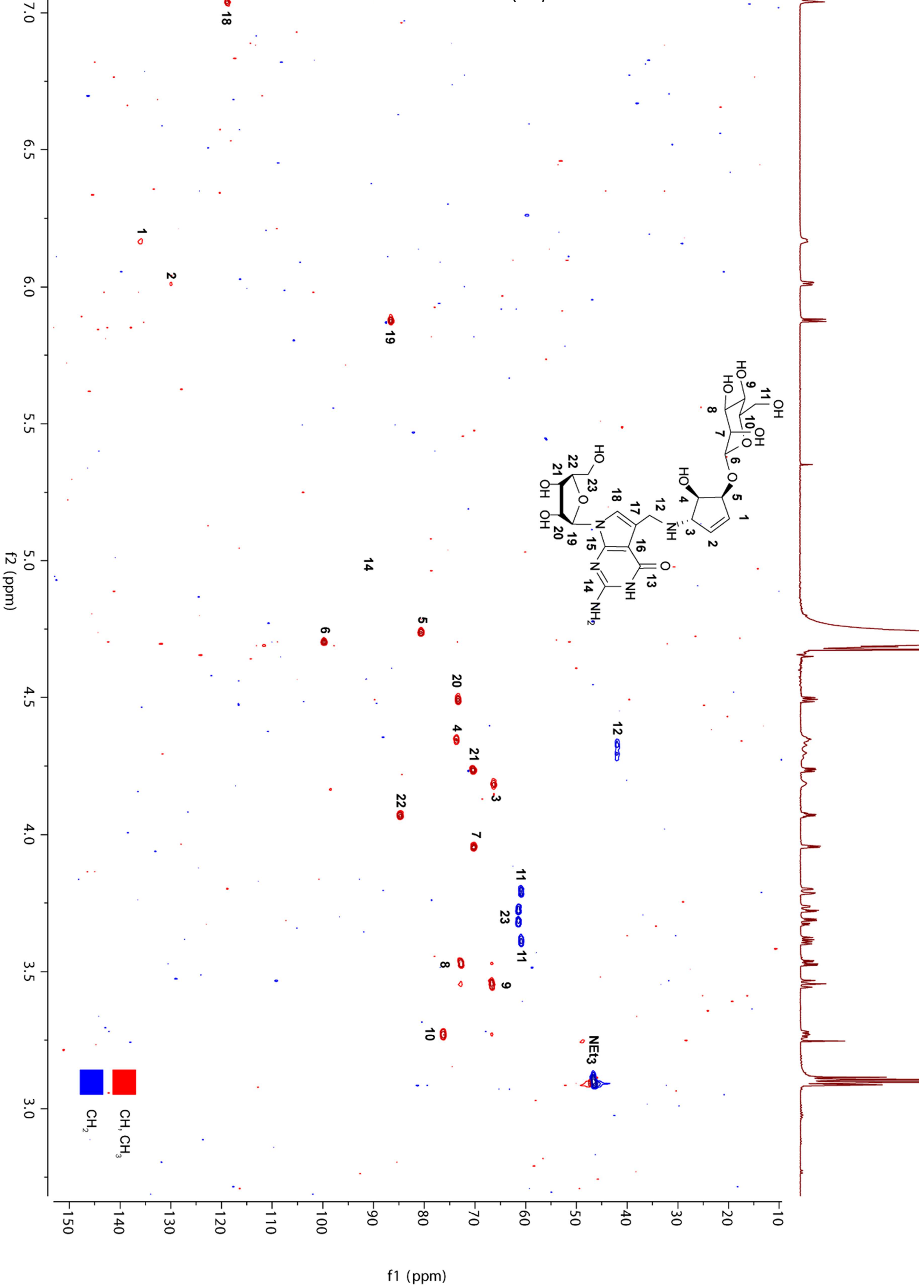

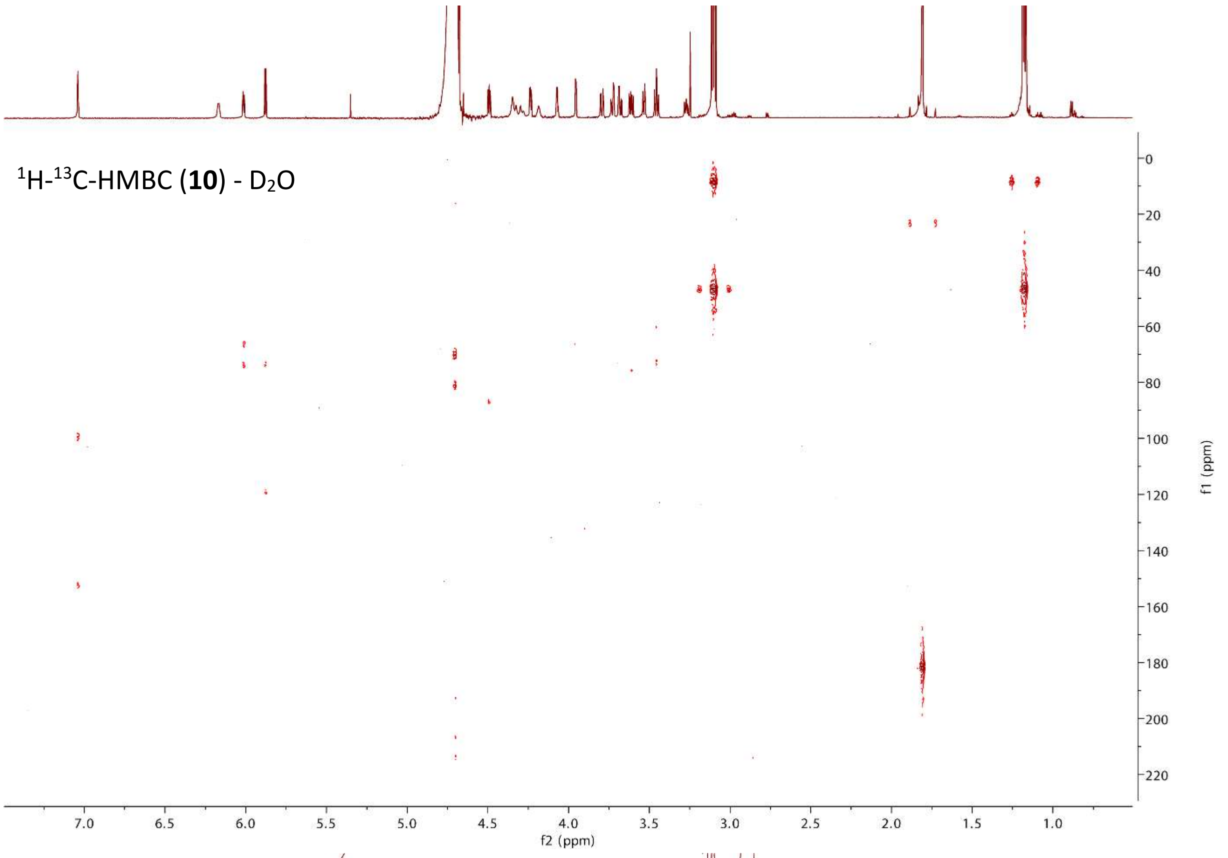

¹H-¹³C-HMBC (10) - D2O
f2 (ppm)
f1 (ppm)

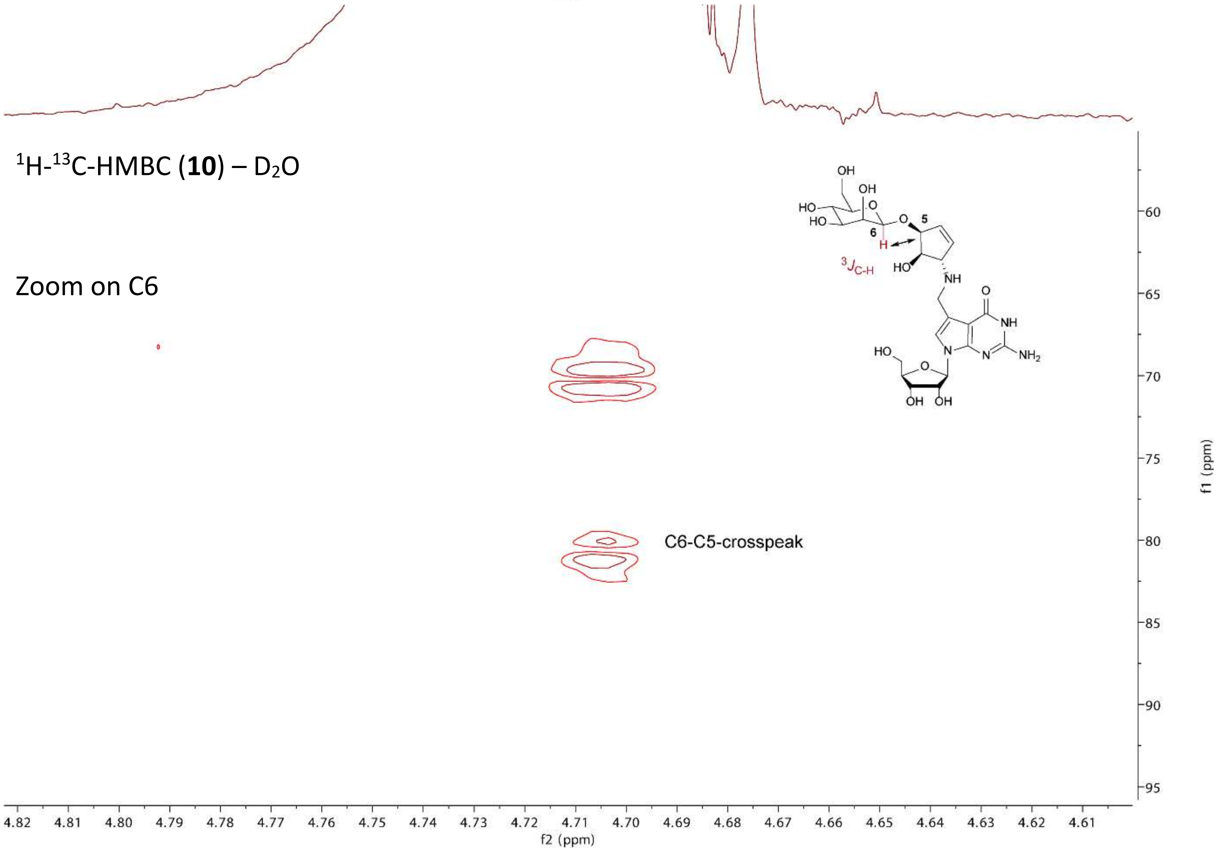

¹H-¹³C-HMBC (10) – D2O
Zoom on C6
C6-C5-crosspeak
f2 (ppm)
f1 (ppm)

^{1}H-^{13}C-HMBC (**10**) – D_2O

Zoom on C18

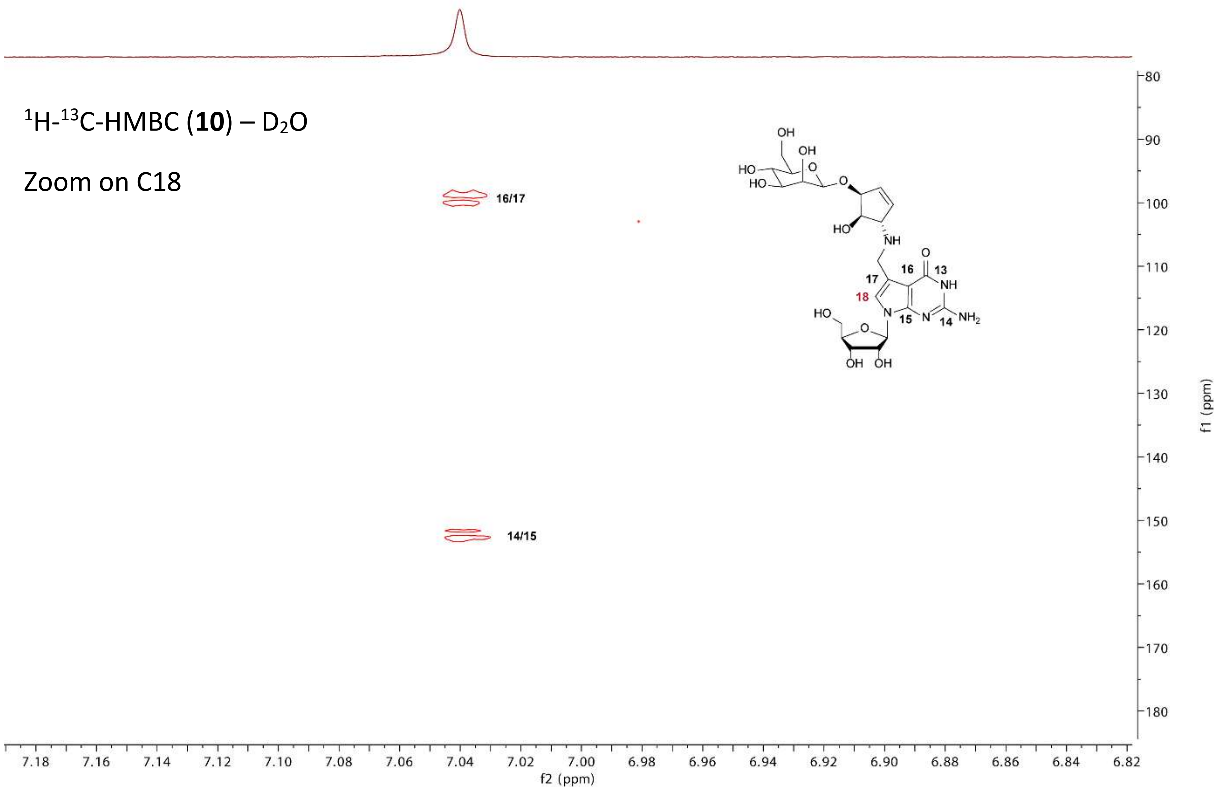

^{1}H-NMR (**11**) – methanol-D$_4$

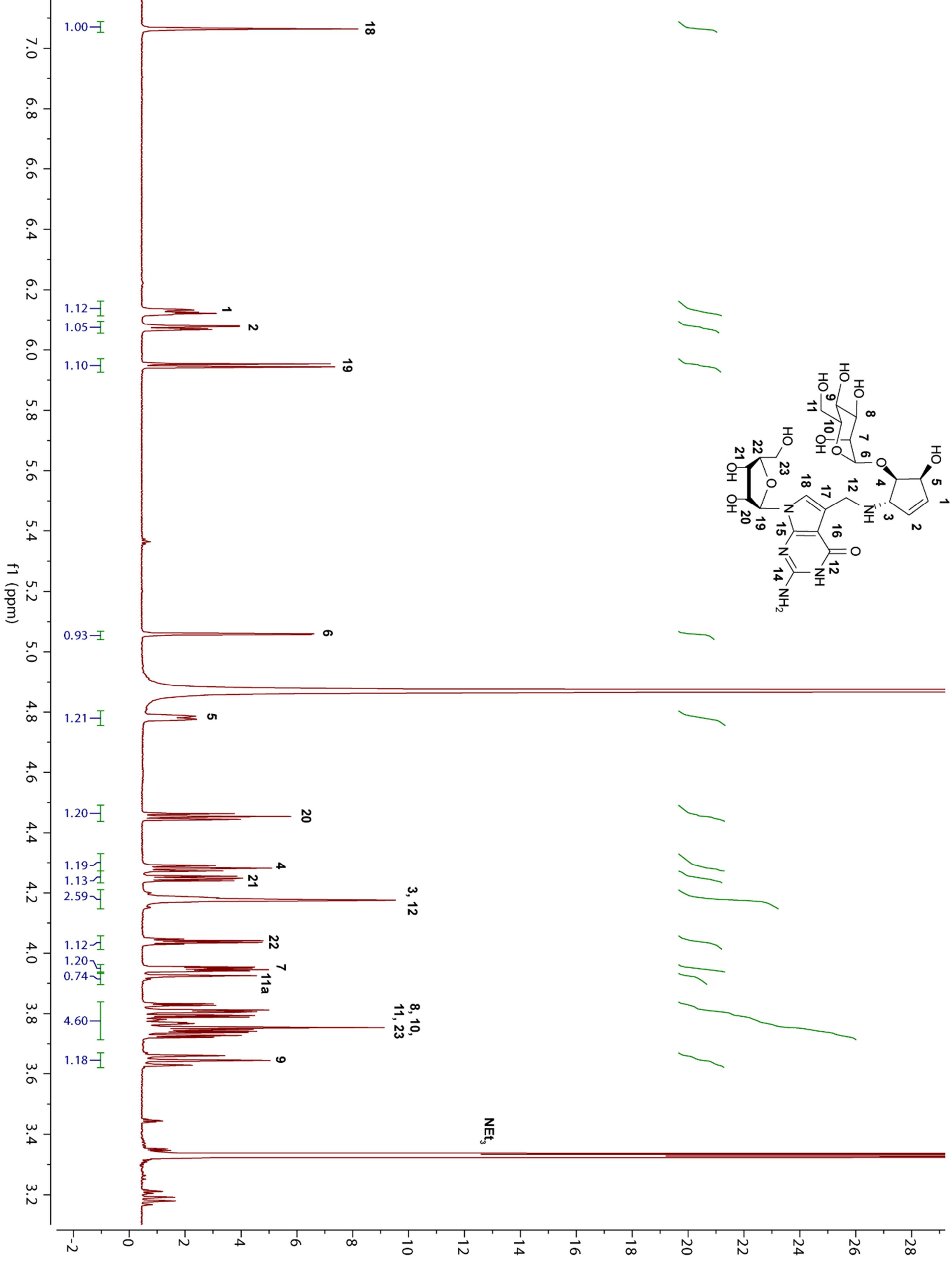

^{13}C-NMR (**11**) - methanol-D$_4$

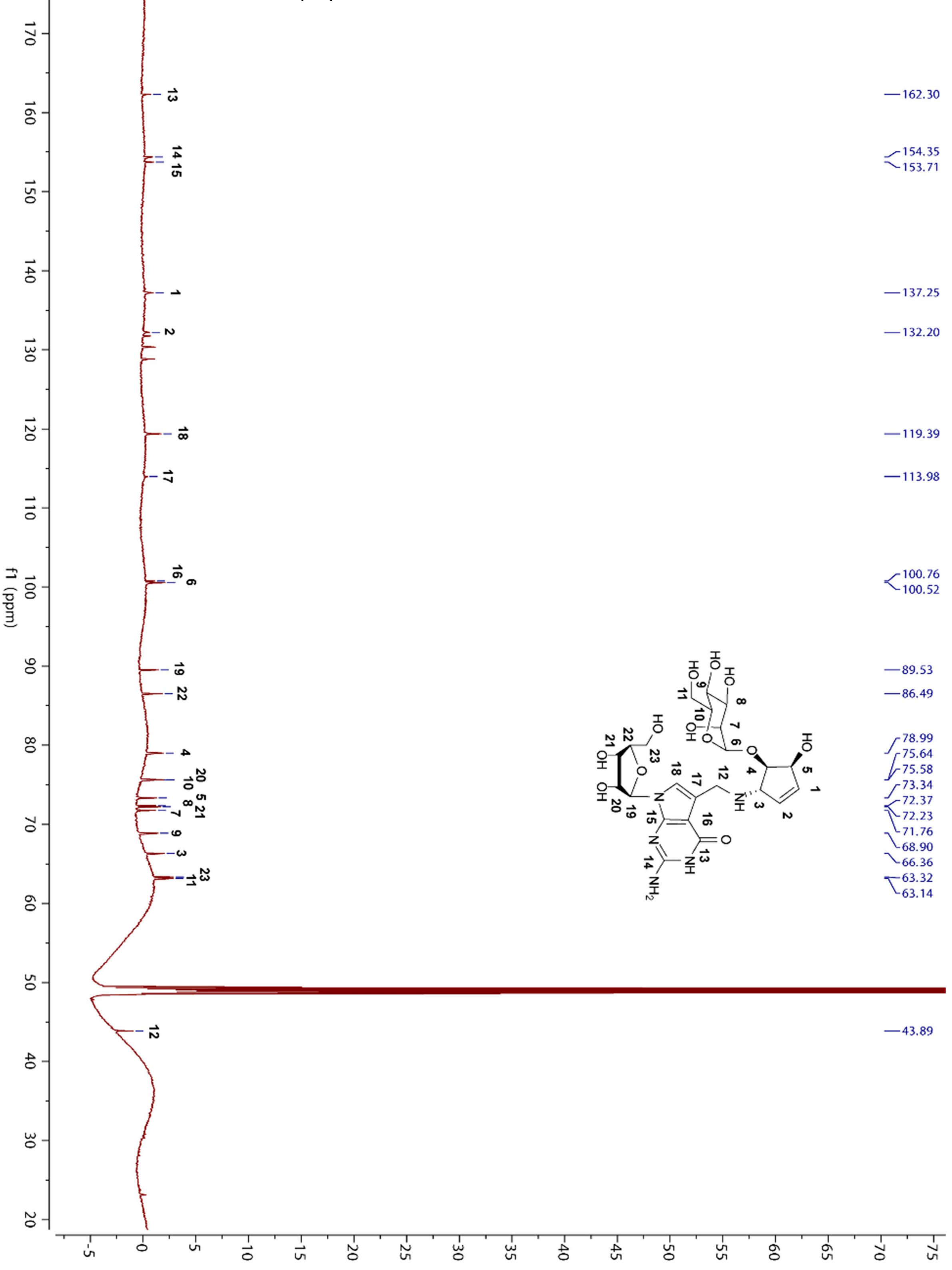

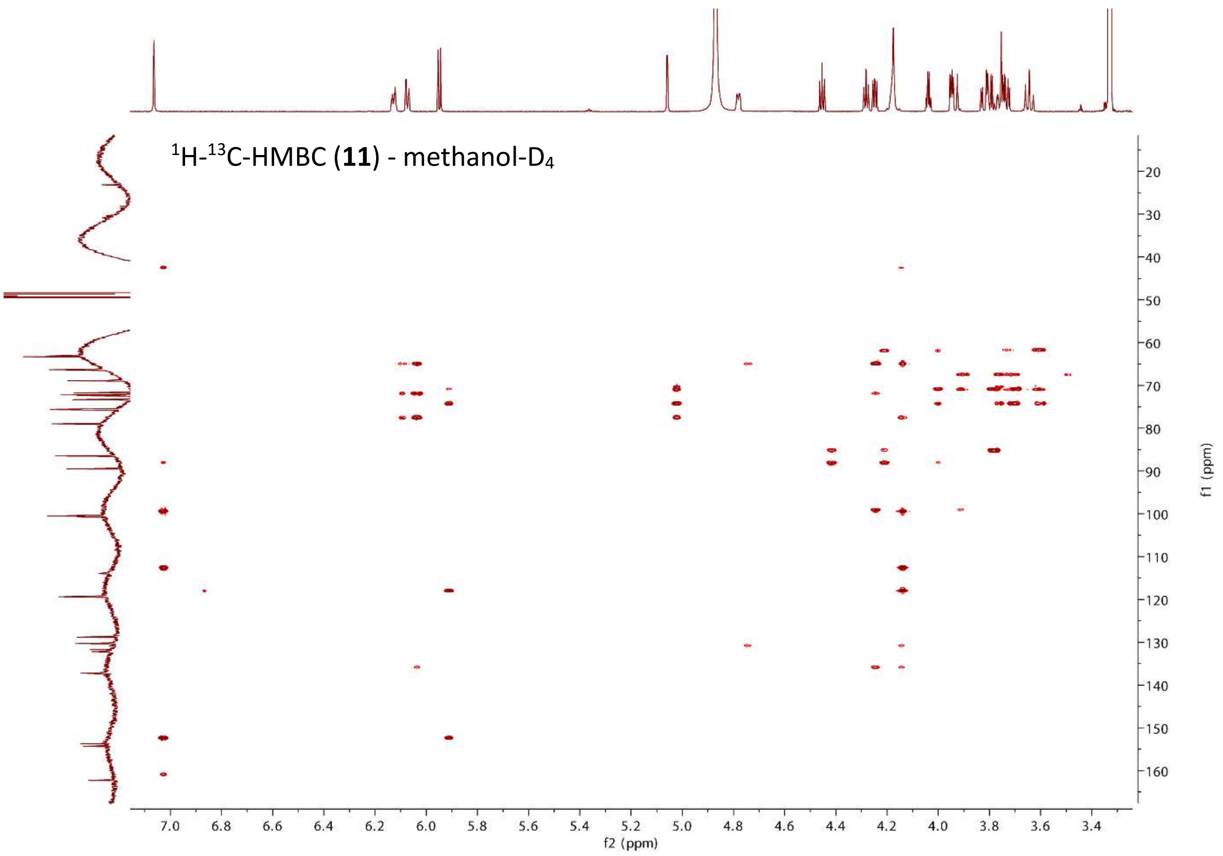

^{1}H-^{13}C-HMBC (**11**) - methanol-D4
f1 (ppm)
f2 (ppm)

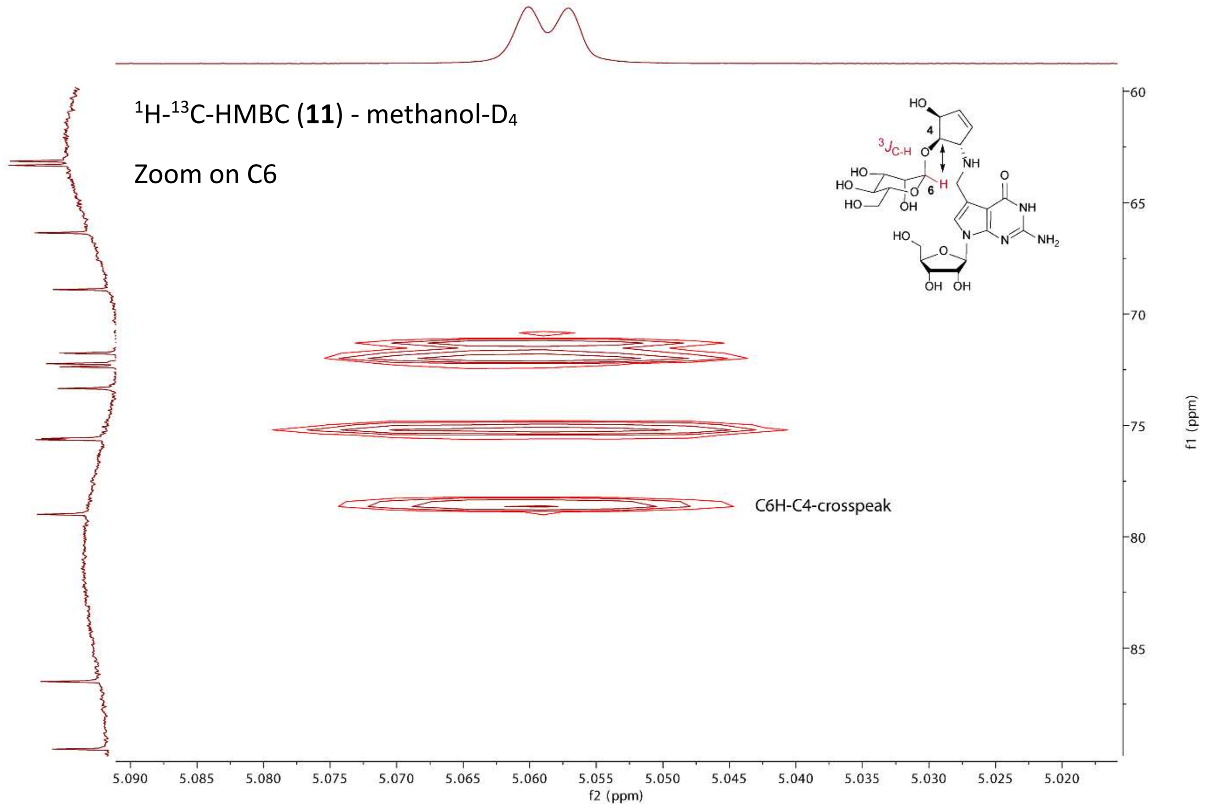

^{1}H-^{13}C-HMBC (**11**) - methanol-D4
Zoom on C6
C6H-C4-crosspeak
f1 (ppm)
f2 (ppm)

^{1}H-NMR (**4**) – D_2O

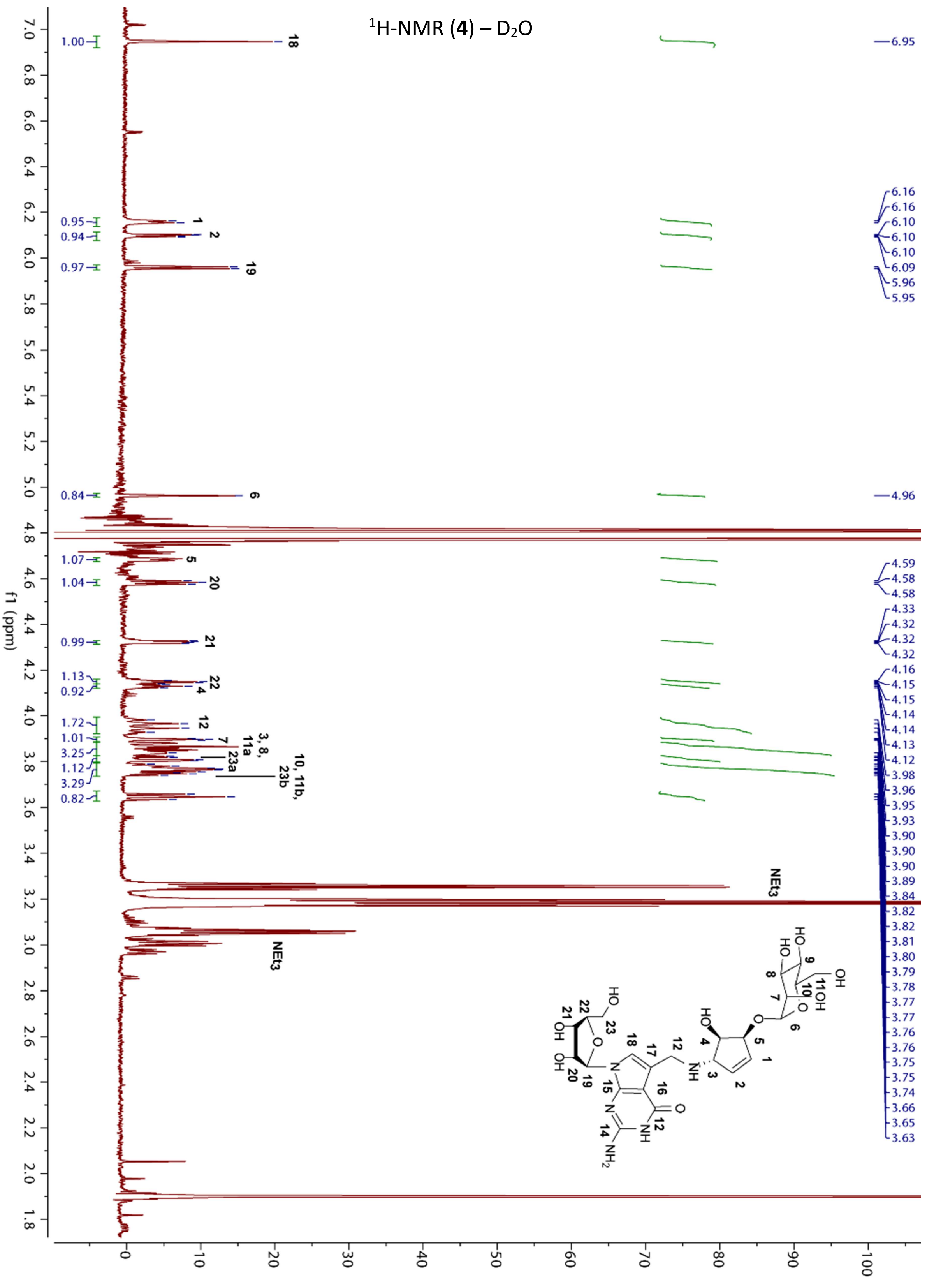

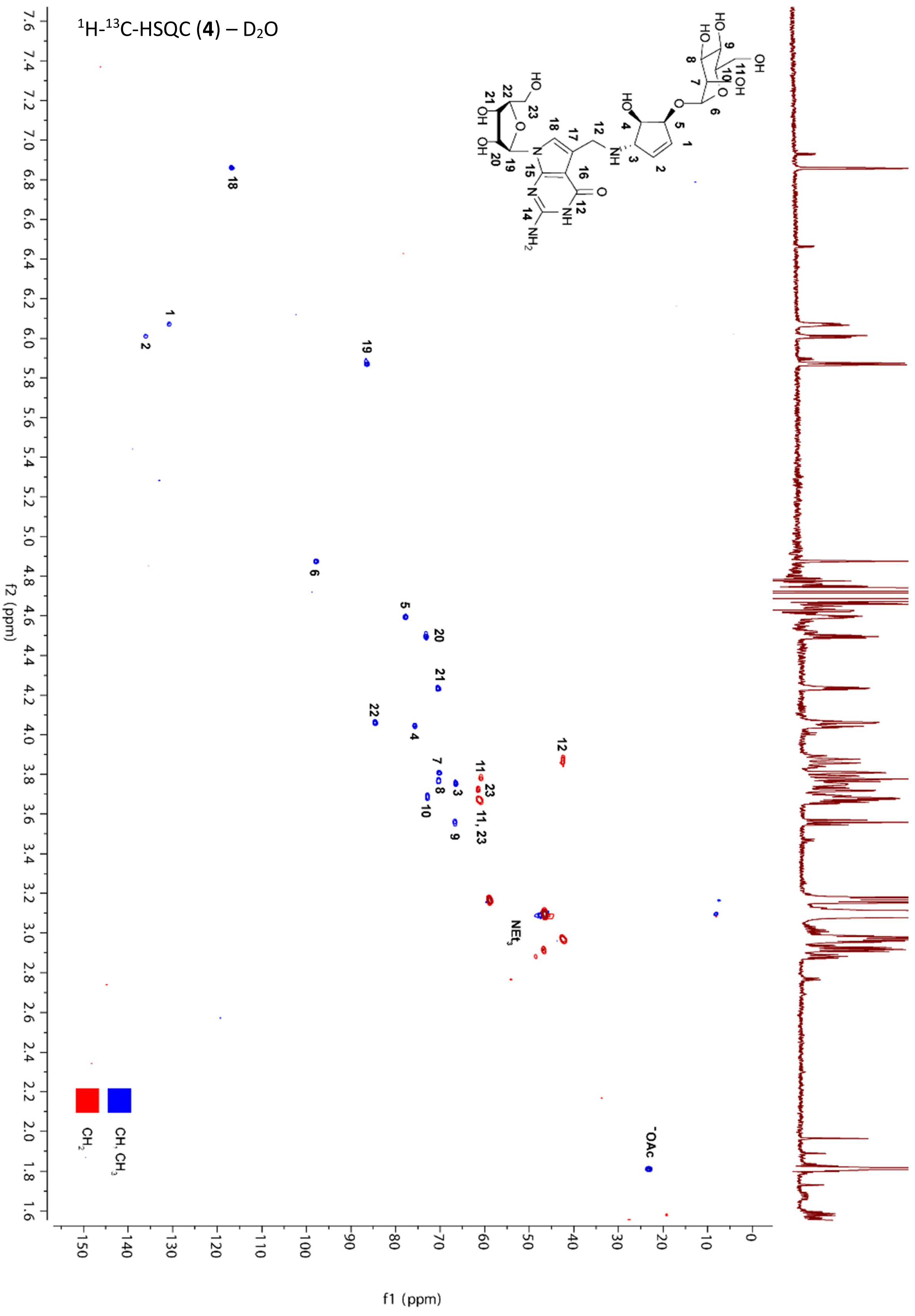

^{1}H-^{13}C-HSQC (**4**) – D_2O
f1 (ppm)
f2 (ppm)
CH, CH_3
CH_2
NEt_3
^{-}OAc

^1H-^{13}C-HMBC (**4**) – D_2O

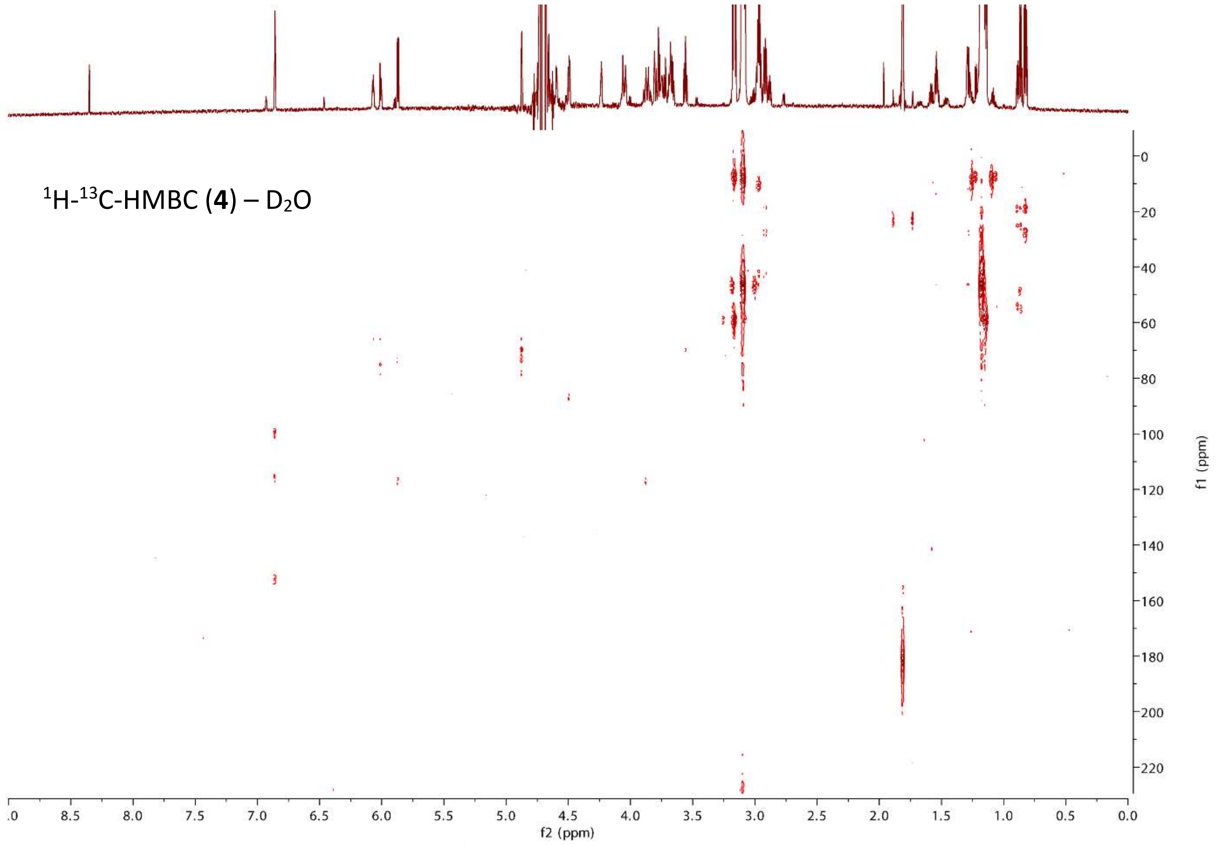

^1H-^{13}C-HMBC (**4**) – D2O

Zoom on C18

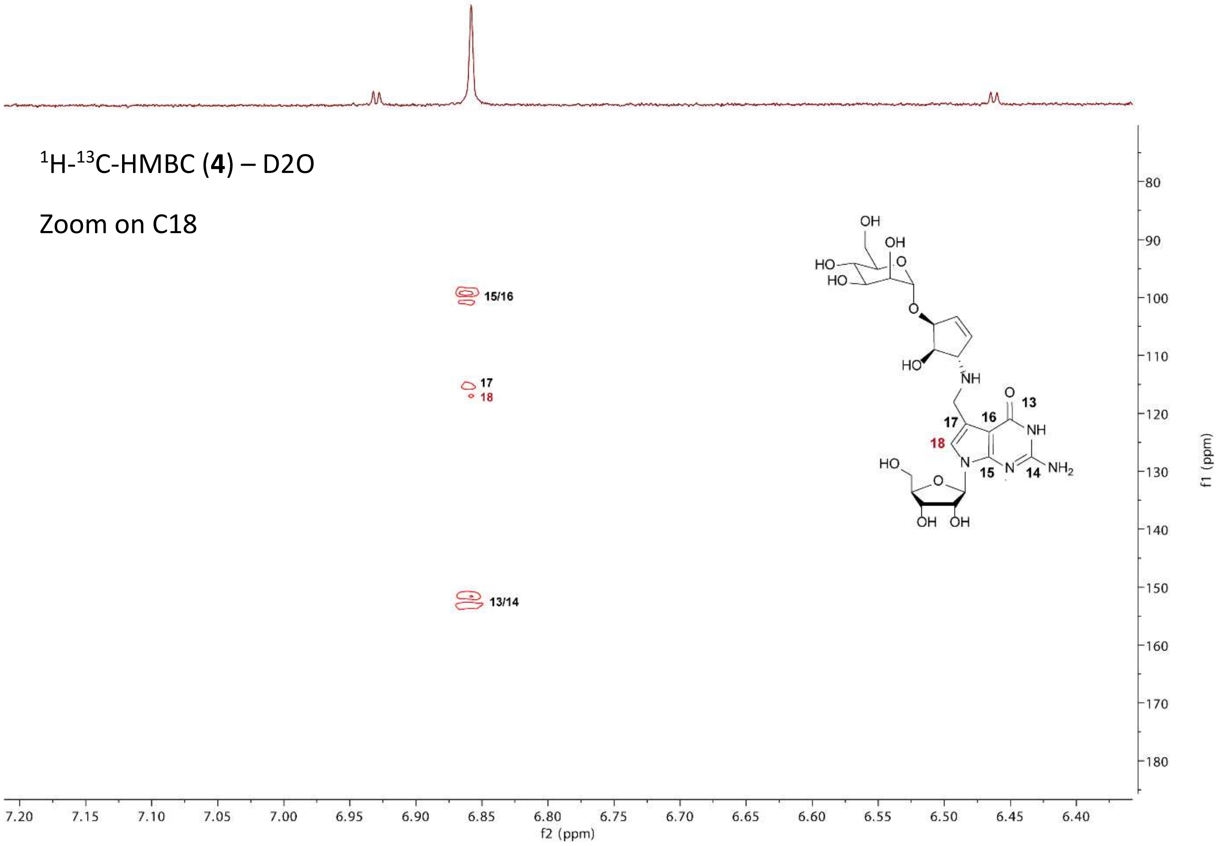

^1H-^{13}C-HMBC (**4**) – D_2O

Zoom on C6

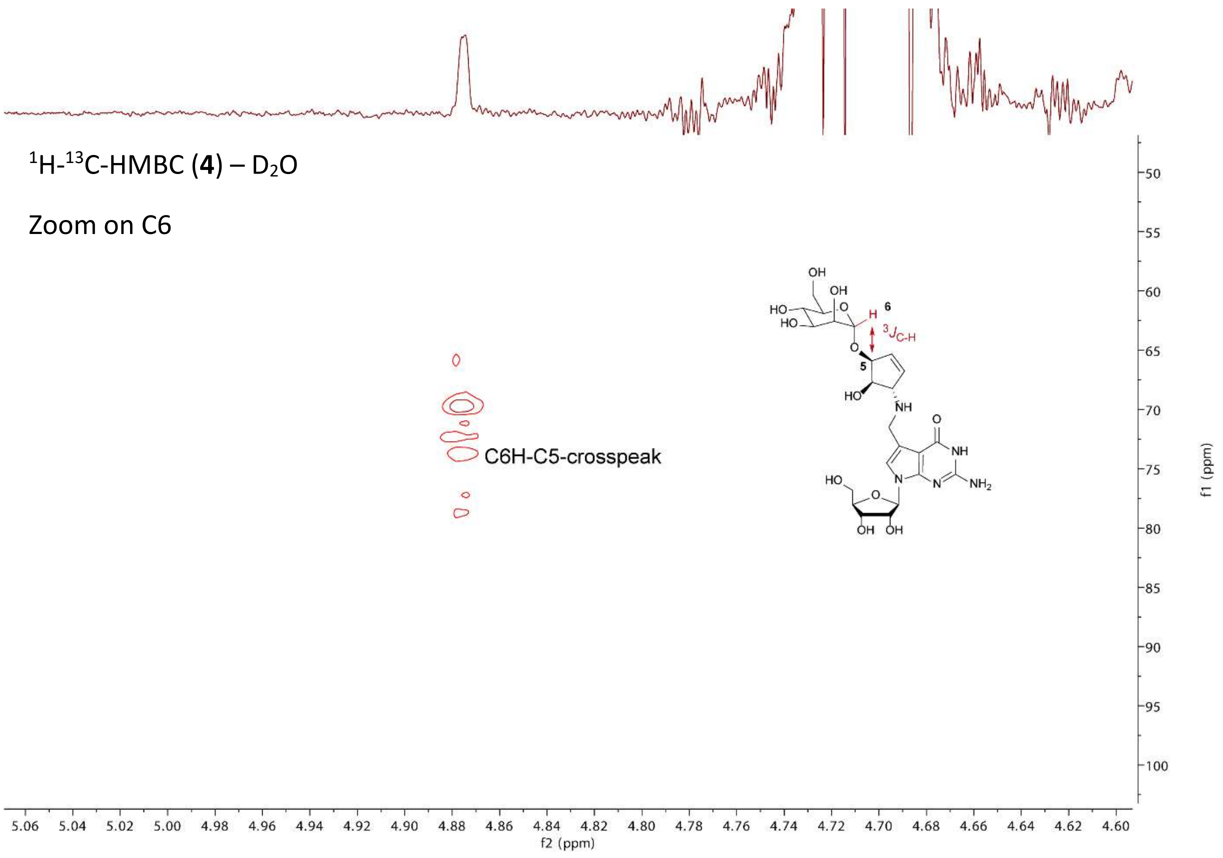

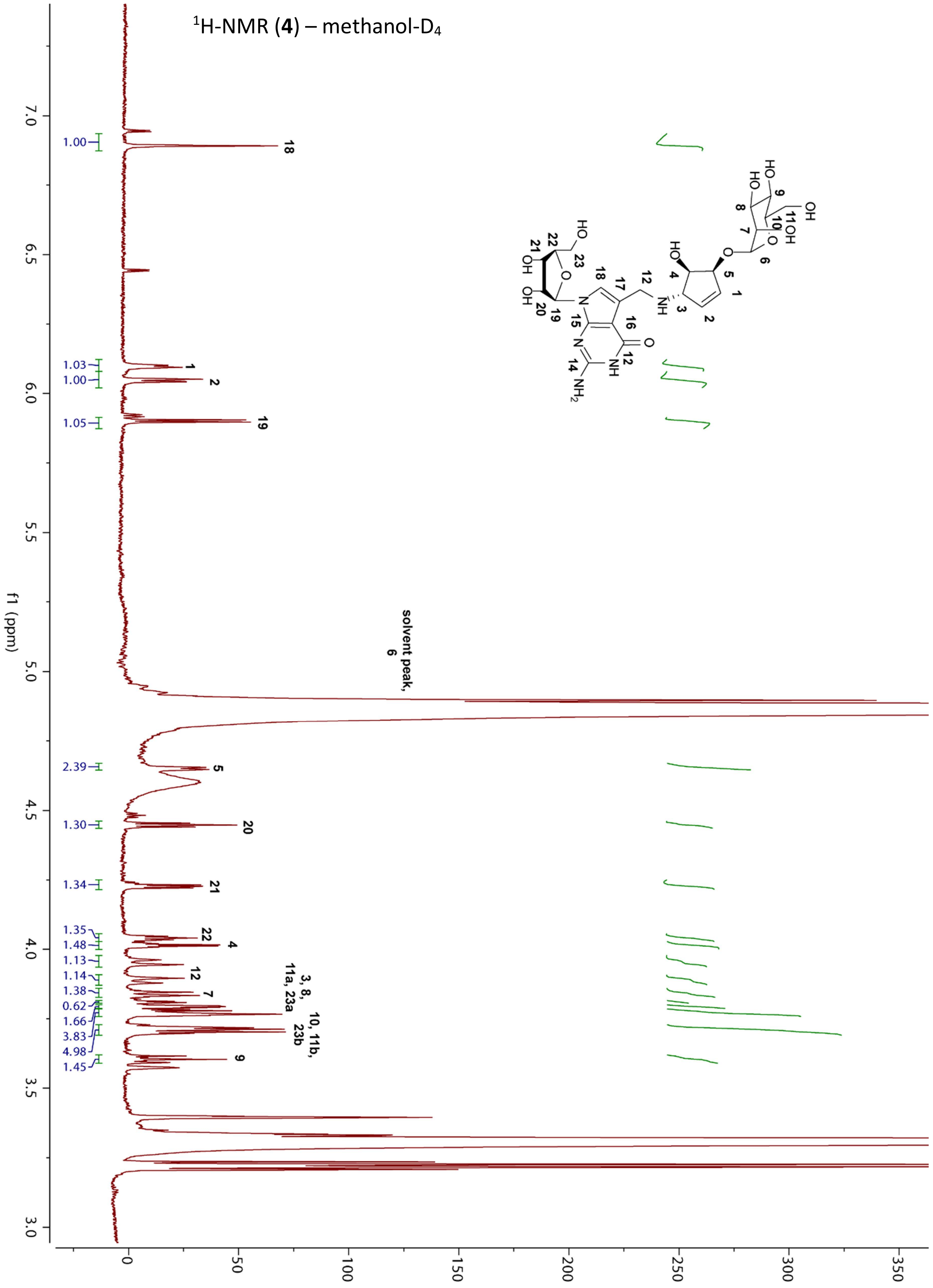

^{1}H-NMR (4) – methanol-D4
f1 (ppm)
solvent peak, 6
3, 8, 11a, 23a
10, 11b, 23b

5. UHPLC-MS/MS chromatograms of co-injection experiments

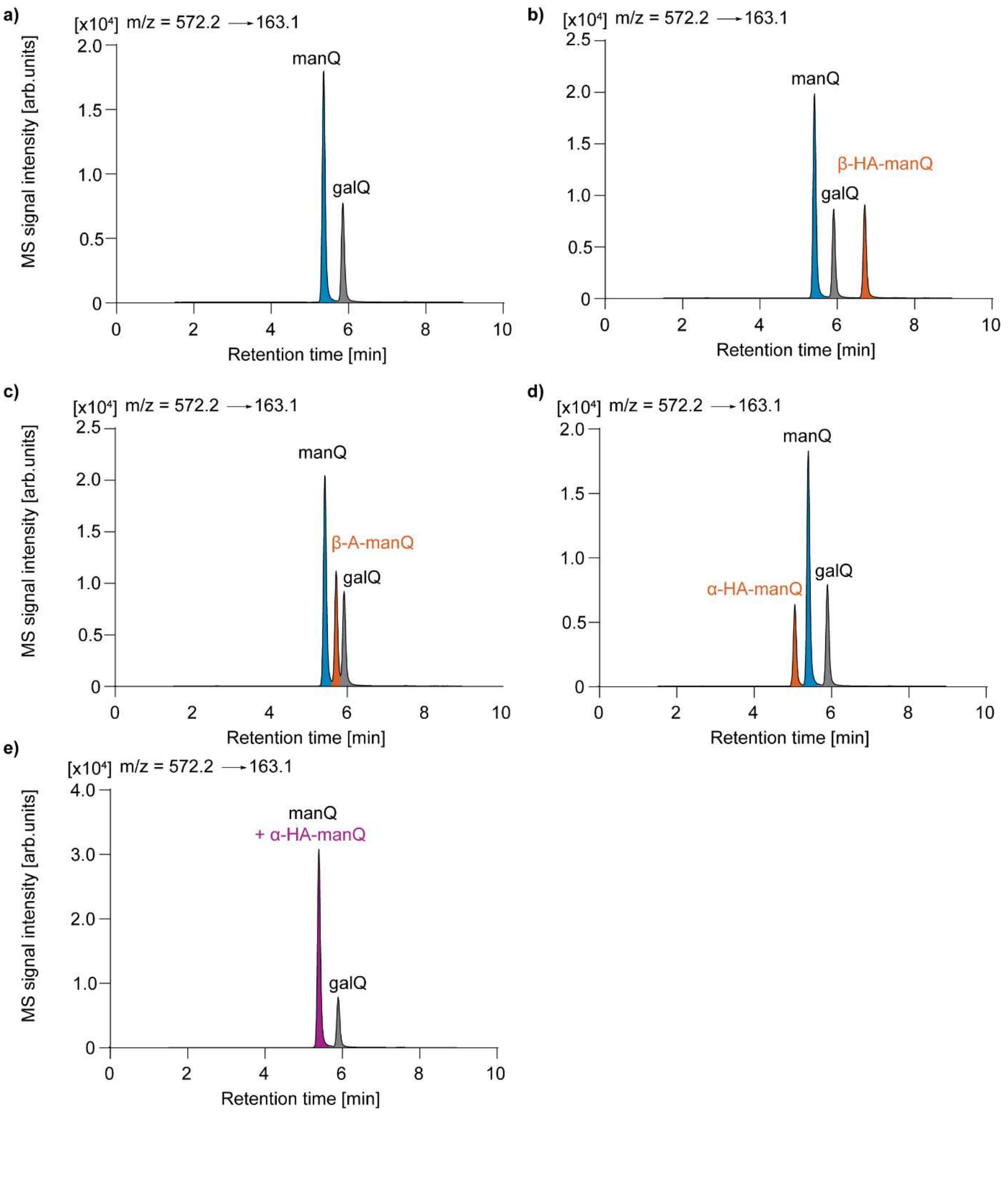

Supplementary Figure 10. Full HPLC-MS/MS-chromatograms of the coinjection experiments performed in this study (corresponding to the chromatograms shown in **Fig. 2** and **5** of the main text). **a**, digested mouse total RNA only; **b**, digested mouse total RNA + β-*homoallyl*-manQ **3**; **c**, digested mouse total RNA + β-*allyl*-manQ **10**; **d**, digested total mouse RNA + α-*homoallyl*-manQ **11**; **e**, digested mouse total RNA + α-allyl-manQ **4**.

6. Supplementary References

1 Thumbs, P. *et al.* Synthesis of Galactosyl-Queuosine and Distribution of Hypermodified Q-Nucleosides in Mouse Tissues. *Angew. Chem. Int. Ed.* **59**, 12352-12356, (2020).

2 Kang, B. *et al.* Carbohydrate-Based Nanocarriers Exhibiting Specific Cell Targeting with Minimum Influence from the Protein Corona. *Angew. Chem. Int. Ed.* **54**, 7436-7440, (2015).

3 Krumb, M., Lucas, T. & Opatz, T. Visible Light Enables Aerobic Iodine Catalyzed Glycosylation. *Eur. J. Org. Chem.* **2019**, 4517-4521, (2019).

4 Chevalier, R. *et al.* Synthetic yeast oligomannosides as biological probes: α-d-Manp (1→3) α-d-Manp (1→2) α-d-Manp and α-d-Manp (1→3) α-d-Manp (1→2) α-d-Manp (1→2) α-d-Manp as Crohn's disease markers. *Tetrahedron* **61**, 7669-7677, (2005).

5 Crich, D. & Li, H. Synthesis of the Salmonella Type E1Core Trisaccharide as a Probe for the Generality of 1-(Benzenesulfinyl)piperidine/Triflic Anhydride Combination for Glycosidic Bond Formation from Thioglycosides. *J. Am. Chem. Soc.* **67**, 4640-4646, (2002).

6 Montenegro, J., Reina, J. & Rioboo, A. Glycosyl Aldehydes: New Scaffolds for the Synthesis of Neoglycoconjugates via Bioorthogonal Oxime Bond Formation. *Synthesis* **50**, 831-845, (2018).

7 Burgula, S., Swarts, B. M. & Guo, Z. Total Synthesis of a Glycosylphosphatidylinositol Anchor of the Human Lymphocyte CD52 Antigen. *Chem. Eur. J.* **18**, 1194-1201, (2012).
