# Supplementary material for: Synthesis and structure elucidation of the human tRNA nucleoside mannosyl-queuosine
Source: Nat Commun. 2021 Dec 8;12:7123. doi: 10.1038/s41467-021-27371-9 (PMC8654956; doi:10.1038/s41467-021-27371-9)
Supplement: Supplementary file 2 — Reporting Summary [file 41467_2021_27371_MOESM2_ESM.pdf]

nature portfolio

Corresponding author(s): Thomas Carell

Last updated by author(s): Oct 18, 2021

Reporting Summary

Nature Portfolio wishes to improve the reproducibility of the work that we publish. This form provides structure for consistency and transparency in reporting. For further information on Nature Portfolio policies, see our Editorial Policies and the Editorial Policy Checklist.

Statistics

For all statistical analyses, confirm that the following items are present in the figure legend, table legend, main text, or Methods section.

n/a	Confirmed	
☐	☒	The exact sample size (n) for each experimental group/condition, given as a discrete number and unit of measurement
☐	☒	A statement on whether measurements were taken from distinct samples or whether the same sample was measured repeatedly
☒	☐	The statistical test(s) used AND whether they are one- or two-sided<br>*Only common tests should be described solely by name; describe more complex techniques in the Methods section.*
☒	☐	A description of all covariates tested
☒	☐	A description of any assumptions or corrections, such as tests of normality and adjustment for multiple comparisons
☐	☒	A full description of the statistical parameters including central tendency (e.g. means) or other basic estimates (e.g. regression coefficient) AND variation (e.g. standard deviation) or associated estimates of uncertainty (e.g. confidence intervals)
☒	☐	For null hypothesis testing, the test statistic (e.g. F, t, r) with confidence intervals, effect sizes, degrees of freedom and P value noted<br>*Give P values as exact values whenever suitable.*
☒	☐	For Bayesian analysis, information on the choice of priors and Markov chain Monte Carlo settings
☒	☐	For hierarchical and complex designs, identification of the appropriate level for tests and full reporting of outcomes
☒	☐	Estimates of effect sizes (e.g. Cohen's d, Pearson's r), indicating how they were calculated

Our web collection on statistics for biologists contains articles on many of the points above.

Software and code

Policy information about availability of computer code

Data collection	Data collection with the TripleQuad LC-MS/MS system: MassHunter Workstation Software Version B.07.01 / Build 7.1.524.0; Data collection with the Orbitrap LC-MS-system: Xcalibur Version 2.1.0 SP1.1160; Chromeleon Version 6.80 SR13 Build 3818 (226197); LTQ Tune Plus Version 2.5.5 SP2.
Data analysis	Analysis of raw data from the TripleQuad LC-MS/MS system: MassHunter Workstation Software Version B.07.01 / Build 7.1.524.0; Analysis of raw data from the Orbitrap LC-MS-system: Xcalibur Qual Browser Version 2.1.0 SP1.1160; General data analysis: Origin 2018 64 bit version.

For manuscripts utilizing custom algorithms or software that are central to the research but not yet described in published literature, software must be made available to editors and reviewers. We strongly encourage code deposition in a community repository (e.g. GitHub). See the Nature Portfolio guidelines for submitting code & software for further information.

Data

Policy information about availability of data

All manuscripts must include a data availability statement. This statement should provide the following information, where applicable:

- Accession codes, unique identifiers, or web links for publicly available datasets
- A description of any restrictions on data availability
- For clinical datasets or third party data, please ensure that the statement adheres to our policy

All data generated and analyzed during this study is included in this article and its Supplementary Information file. Chromatograms of co-injection experiments are provided in the article figures and Methods section. Synthesis protocols for and NMR-spectra of all relevant compounds, proving their chemical identity and bulk purity, are given in the Supplementary Information.

Field-specific reporting

Please select the one below that is the best fit for your research. If you are not sure, read the appropriate sections before making your selection.

- [x] Life sciences
- [ ] Behavioural & social sciences
- [ ] Ecological, evolutionary & environmental sciences

For a reference copy of the document with all sections, see nature.com/documents/nr-reporting-summary-flat.pdf

Life sciences study design

All studies must disclose on these points even when the disclosure is negative.

Sample size	Three independent experimental replicates in case of the metabolic isotope labelling experiments. No statistical methods were used to predetermine sample size. Sample size was chosen based on costs, experience and common sample sizes of the respective method.
Data exclusions	none.
Replication	Co-injection experiments performed for LC/MS-based structure elucidation of manQ isomers: Successful independent replication of results on two different LC-MS systems using distinct chromatography columns and solvent gradients.
Randomization	Not applicable, randomization is not required for our structural elucidation study.
Blinding	Not applicable, blinding was not implemented in this study as for all experiments, analyses were inherently objective.

Reporting for specific materials, systems and methods

We require information from authors about some types of materials, experimental systems and methods used in many studies. Here, indicate whether each material, system or method listed is relevant to your study. If you are not sure if a list item applies to your research, read the appropriate section before selecting a response.

Materials & experimental systems

n/a	Involved in the study	
☒	☐	Antibodies
☐	☒	Eukaryotic cell lines
☒	☐	Palaeontology and archaeology
☒	☐	Animals and other organisms
☒	☐	Human research participants
☒	☐	Clinical data
☒	☐	Dual use research of concern

Methods

n/a	Involved in the study	
☒	☐	ChIP-seq
☒	☐	Flow cytometry
☒	☐	MRI-based neuroimaging

Eukaryotic cell lines

Policy information about cell lines

Cell line source(s)	HEK293T cells provided by CLS Cell Lines Service GmbH, Eppelheim, Germany; catalog number 300192.
Authentication	Microscopic control of morphology.
Mycoplasma contamination	All cell lines were tested negative for Mycoplasma contamination in a PCR-based test (Jena Bioscience Mycoplasma Detection Kit PP-401L). Additionally, no RNA modifications indicative for mycoplasma contamination were detected in the course of our LC/MS-analyses.
Commonly misidentified lines (See ICLAC register)	HeLa cells
